# The British Association for Cancer Research, 23rd annual general meeting. Edinburgh 29--31 March 1982. Abstracts.

**DOI:** 10.1038/bjc.1982.227

**Published:** 1982-09

**Authors:** 


*Br. J. Cancer* (1982) **46**, 459

### THE BRITISH ASSOCIATION FOR CANCER RESEARCH
### 23RD ANNUAL GENERAL MEETING

### Held at Edinburgh 29–31 March 1982

The Association gratefully acknowledges financial support from the following:

The Imperial Cancer Research Fund
The Cancer Research Campaign
Farmitalia Carlo Erba Limited
Lederle Laboratories

**DEVELOPMENT OF METASTATIC HETEROGENEITY IN MALIGNANT TUMOURS.** I. R. Hart, *Cancer Metastasis and Treatment Laboratory, NCI-Frederick Cancer Research Facility, Frederick, Maryland* 21701 *U.S.A.*

The process of metastasis selects for metastatic subpopulations of cells that pre-exist within the primary tumour. Regardless of whether the parent neoplasm is unicellular or multicellular in origin, it can become heterogeneous with regard to the metastatic phenotype within a short time. Metastatic cells appear to be less stable genetically and phenotypically than benign cells; this may account for the ability of metastatic cells to survive under strong selection pressures, such as those imposed during tumour spread, and to emerge as progenitors of secondary growths. Tumour cell subpopulations of clonal origin are phenotypically less stable than polyclonal populations, perhaps because an equilibrium exists among these heterogeneous subpopulations; a situation which would tend to limit further diversification. The development of metastatic heterogeneity in malignant tumours and the selective nature of the metastatic process have profound implications for biological studies of tumour spread.

**BIOLOGICAL AND CLINICAL STUDIES OF CLONOGENIC HUMAN TUMOUR CELLS.** S. E. Salmon, *University of Arizona Cancer Centre, Tucson, Arizona* 85724, *U.S.A.*

Since 1975, our group has worked on the development of an *in vitro* clonogenic assay for fresh biopsy samples, apply the technique to biological diagnostic studies as well as chemosensitivity testing. We have explored host–tumour cell interactions *in vitro* and found that the endogenous adherent macrophages were responsible for most of the prostaglandin production as well as modulating tumour growth. Adherent phagocytic macrophages often appear to stimulate the clonal proliferation of the tumour cells in this culture system. Evidence that cells being tested *in vitro* have relevance to tumour stem cells *in vivo* has also been found. In a series of 4000 *in vitro* tests with standard cytotoxic agents, significant activity against TCFU was seen in 5–20% of tumours tested, with variation among tumour types. We have conducted a series of prospective clinical trials of the assay in relation to clinical response and survival. Overall, for a number of tumour types, the assay predicts drug sensitivity with 60–70% accuracy, and drug resistance with 95% accuracy. Accurate prediction of response in melanoma is somewhat less common, unless "mixed" responses are included. In both myeloma and ovarian cancer, survival is better in patients who are sensitive *in vitro* than in those who are resistant. The assay also has major applications to chemosensitivity testing with new agents for "*in vitro* Phase II trials", and can be used to target tumour types as well as to predict required plasma concentrations for new agents. The National Cancer Institute is also exploring the application of this assay system for pre-clinical use, in screening for entirely new types of anticancer drugs.

**GROWTH FACTORS FOR CULTURED MAMMARY AND FIBROBLASTIC CELLS.** P. S. Rudland & J. A. Smith, *Ludwig Institute, Royal Marsden Hospital, Sutton, Surrey*

The growth of the mammary gland is controlled by a series of interacting mammotrophic hormones. However these hormones are inactive compared with serum or novel, semi-purified growth factors (GF) in stimulating cell proliferation of cultured rat mammary-gland epithelial cells. These GFs are of 2 types, a pituitary GF exerting systemic control, and GFs from surrounding cells which may exert local control of mammary epithelial-cell proliferation. The effects of the GFs can be amplified by certain mammotrophic hormones (e.g. insulin (I) and hydrocortisone (HC)). As in the mammary gland, GFs such as FGF, EGF, $PGF_{2\alpha}$ can stimulate DNA synthesis in quiescent 3T3 fibroblasts, which can be modified with I and HC. To explain the kinetics obtained with $PGF_{2\alpha}$ and FGF, 2 operational signals are postulated, signal (1) initiates a lag phase and signal (2) determines the magnitude of the rate of cellular entry into the S phase of the cell cycle. Usually the rate of entry into S governs the rate of cell division, but when serum is completely removed, additional components (e.g. transferrin) are required.

During the lag phase the pathways generated by signal (1) and signal (2) have to interact, causing increases in hypothetical protein(s) inside the cell. One of the altered properties of virally transformed cells is their lack of response to GFs. In the 2 cases examined by other groups the viral transforming proteins directly act on the putative intracellular pathways, leading to increased cell-proliferation rates. This does not rule out the possibility of autocrine stimulation by GFs in other systems, but suggests that some of the intracellular steps are the more vulnerable to alterations which lead to uncontrolled proliferation of potentially neoplastic cells.

**MURINE T-LYMPHOCYTE CLONES: DERIVATION, CHARACTERIZATION AND APPLICATION TO THE STUDY OF ALLOGRAFT AND TUMOUR IMMUNITY.** H. R. MacDonald, H. D. Engers, R. K. Lees, A. L. Glasebrook, A. Kelso, B. Sordat, J.-C. Cerottini & K. T. Brunner, *Ludwig Institute for Cancer Research, Lausanne Branch, and Dept. Immunology, ISREC, Epalinges, Switzerland*

Clones of murine T-lymphocytes specific for a variety of soluble and cell-bound antigens can now be established and grown in the presence of T cell growth factor (TCGF). We have used this cloning technology to study the immune response to alloantigens and to murine leukaemia virus (MoLV)-associated antigens. T-lymphocyte clones in both antigenic systems were found to have various functional phenotypes, as defined by cytolytic activity, and the production of lymphokines such as TCGF, interferon, macrophage-activating factor (MAF), and granulocyte/macrophage colony stimulating factor (GM-CSF).

Two approaches were used to investigate the possible role of T-lymphocyte clones in allograft and tumour immunity *in vivo*. In one experimental model, cloned cytolytic T-lymphocytes (CTL) were injected i.p. together with dissociated suspensions of $^{131}$IdU-labelled tumour target cells bearing relevant alloantigens or MoLV-associated antigens. In both cases, efficient tumour-cell destruction was obtained; however, when cloned CTL were injected i.v., only certain clones were capable of recirculating and destroying tumour cells in the peritoneal cavity. In a second model better simulating the complexity associated with solid tumours *in vivo*, multicellular spheroids of mammary tumour cells were implanted i.p. and exposed to cloned CTL. Again a destruction of tumour spheroids by some (but not all) CTL clones was noted. Taken together, these data indicate that cloned T-cells warrant serious consideration as an immunologically specific therapeutic tool in allograft and tumour immunity.

**NATURAL CELL-MEDIATED CYTOTOXICITY AS A POSSIBLE ANTI-TUMOUR SURVEILLANCE MECHANISM.** O. Stutman, *Cellular Immunology Section, Memorial Sloan-Kettering Cancer Center, New York, N.Y., U.S.A.*

Natural cell-mediated cytotoxicity (NCMC) is detected in normal animals as the capacity of their lymphoid cells to lyse a variety of tumour cells *in vitro*. Based on surface-antigen characteristics, genetic control as well as other properties, 2 major types of effector cells have been described: natural killer (NK) and natural cytotoxic (NC) cells. Both types do not appear to belong to any conventional lymphoid or haemopoietic cell lineage. One of the original differences, i.e. the preference of the former for lymphoid tumours and of the latter for solid tumours, is not that general, since using either resting or boosted (with poly-IC) effector cells, almost all the possible permutations can be detected if enough targets are studied (i.e. solid tumours lysed exclusively by resting or boosted NK cells, solid tumours lysed by resting NC and boosted NK, etc.) This is not surprising since "NK susceptible" and "NC susceptible" targets share "antigenic" determinants based on either cold-target inhibition studies or by the capacity of monosaccharides to block NCMC *in vitro*. The fact that NCMC exists at relatively high levels in normal animals and does not need time-consuming activation like the more conventional immune responses, that the system can deal preferentially with small numbers of tumour cells and that it is independent of conventional "tumour-associated" antigens, make NCMC a likely candidate for exerting immunosurveillance *in vivo*, in the sense defined by Burnet. Unfortunately, examples which support and negate such interpretation are available.

### COMPARISON OF CLONOGENIC ASSAY METHODS ON PLASTIC FOR MEASURING DRUG SENSITIVITY *IN VITRO*.

P. J. Hepburn, J. R. W. Masters & B. T. Hill, *Institute of Urology and Imperial Cancer Research Fund, London*

Although clonogenic assays provide an established method for measuring *in vitro* drug sensitivity, little is known concerning the intercomparability of the various methods. The purpose of this study was to investigate some of the variables, including (a) comparison of transferring cells immediately (CFE) or 25 h after drug treatment (CFE/24) with leaving colonies to develop *in situ* (CFA), (b) effect of feeder cells (3T3) and (c) differences between cells in logarithmic (Exp) and "Pre-Exp" phase growth. The effect of these variables on sensitivity to a class II (methotrexate) and a class III (adriamycin) drug were compared using a continuous cell line from a human bladder cancer, RT112.

Classic *in vitro* patterns of sensitivity were found using exponentially growing cells transferred immediately on to plastic following drug exposure. However, using other methods, the concentration required to reduce survival by 50% ($ID_{50}$—see Table) extended over a 150- and 5-fold range, respectively, for methotrexate and adriamycin. In conclusion we confirmed that significant differences in the pattern of drug sensitivity *in vitro* can result from variations in clonogenic assay procedure.

| | $ID_{50}$ (mg/ml) | | $ID_{50}$ (mg/ml) | |
|---|---|---|---|---|
| | Pre-Exp MTX | Exp | Pre-Exp ADR | Exp |
| CFE | 0·3 | 11·4 | 11·0 | 7·4 |
| CFE/24 | 40·9 | 15·9 | 4·4 | 7·3 |
| CFA | 45·4 | | 17·4 | |
| CFE-3T3 | 4·1 | 18·2 | 14·5 | 5·8 |
| CWE/24 | 40·9 | 25·0 | 5·8 | 17·4 |
| CFA-3T3 | 15·9 | | 1·8 | |

### CLONING OF HUMAN TUMOUR STEM CELLS. SUCCESS OF DRUG SENSITIVITY TESTING.

A. P. Simmonds & E. C. McDonald, *Biochemistry Department, Royal Maternity Hospital, Glasgow*

The *in vitro* stem-cell assay system (Hamburger & Salmon, 1977 *Science*, **197**, 461) has been used to grow ovarian tumour material and effusions from breast and gastric cancer, also osteosarcoma, melanoma and mesothelioma. Predictive testing for drug sensitivities (Salmon *et al.*, 1978, *N. Engl. J. Med.*, **298**, 1321) has been done using cis-platin, adriamycin, methotrexate and vindesine. 88% of samples in the ovarian group, comprising 60 patients and 88 samples, were cultured successfully, and significant results for cisplatin response achieved for 37 patients. Five of these were also tested against ADR. Only 12 patients received drugs of test, of whom 6/7 at present evaluated clinically show good correlation between test and outcome. Effusions from other malignancies (8 breast, 2 mesothelioma and 1 each of gastric, osteosarcoma and melanoma) were cultured successfully, and correlation between test and outcome found in 6/7 cases evaluated so far. Failure to obtain drug results is due to (a) paucity of sample, or (b) low plating efficiency in culture. Clearly this method needs further evaluation as part of a clinical trial, where the drugs of chemotherapy are known at the time of assay. Although 13 of the 60 ovarian patients received no chemotherapy, it should be possible to test 3 drugs which a patient might receive.

### *IN VITRO* DRUG SENSITIVITY OF HUMAN OVARIAN TUMOUR CELLS TO *CIS*-DICHLORODIAMMINE PLATINUM (CDDP).

A. P. Wilson C. E. Newman, C. H. J. Ford & A. Howell, *Dept. of Obstetrics & Gynaecology, Clinical Research Block, Withington Hospital, Manchester*

28 human ovarian tumours have been assessed for their sensitivity to CDDP in a monolayer assay using depression of $^3H$-leucine incorporation as an index of cell death. At 1 $\mu$g/ml of CDDP tumours could be divided into 3 categories (i) sensitive, $<35\%$ of control, (ii) intermediate, 34–45% of control, (iii) resistant, $>45\%$ of control. Clinical data are available for 4 previously untreated patients and 7 receiving 5 courses of 100 mg/m$^2$ CDDP. In the untreated group, 2 patients with sensitive tumours and one patient with an intermediate tumour had complete responses to CDDP treatment, whilst one patient with an intermediate tumour had progressive disease after 2 courses of CDDP. In the treated group one tumour was sensitive and 3 were intermediate, suggesting that full resistance to CDDP had not developed. Comparison between sensitivity to CDDP and other drugs in the same assay system revealed a marked correlation between CDDP and Cyclophosphamide (CY) sensitivity. 93% (12/13) of tumours resistant to CDDP were also resistant to CY whilst 53% (8/15) which were sensitive to CY. Similar trends were observed with melphalan, bleomycin, chlorambucil and vinblastine, but not with 5-fluorouracil or adriamycin. The unique sensitivity of some tumours to CDDP was a notable finding.

### CLONOGENICITY OF HUMAN GLIA IN SUSPENSION.

R. I. FRESHNEV & E. HART, *Department of Oncology, University of Glasgow*

Macpherson and Montagnier (*Virology* 1964, **23**, 291) demonstrated that polyoma virus-induced malignant transformation of hamster fibroblasts was accompanied by a substantial increase of cloning efficiency in soft agar. Unfortunately, short-term cell cultures derived from human tumours do not clone in suspension with the high efficiency of transformed rodent fibroblasts, making this a less satisfactory criterion of malignancy. We have shown, moreover, that the capacity to form colonies in suspension may be found in some normal solid tissue cells also.

Cells from a number of short-term cell lines were plated between $5 \times 10^4$ and $5 \times 10^5$ per 35 mm dish or $10^3$–$10^4$ per well in microtitration plates. A methocel upper layer and agar lower layer was used at varying concentrations, and optimal cloning was obtained in microtitration dishes with a 0·5% agar underlay and 0·8% methocel overlay. Successful cloning was achieved with several lines of normal glia and fibroblasts as well as with glioma and melanoma using a lower limit of 32 cells per colony. Optimization of the conditions did not discriminate between cultures derived from normal or malignant tissue.

We conclude that for certain types of cell, at least, the formation of colonies in methocel/agar does not correlate with their malignancy.

### COLONY GROWTH AND CHEMOSENSITIVITY OF HUMAN MELANOMAS IN A SOFT-AGAR ASSAY.

K. M. TVEIT, S. VAAGE, S. GUNDERSEN & A. PIHL, *Norsk Hydro Institute for Cancer Research and The Norwegian Radium Hospital, Oslo, Norway*

The ability of human melanoma cells from patients' metastases to form colonies in soft agar was examined using the method of Courtenay & Mills (*Br. J. Cancer*, 1978, **37**, 261). Relatively high plating efficiencies (PEs) were obtained. Thus, in a study of 150 metastases, 27% of the tumours gave PE 1%, 44% gave PEs of 0·1–0·9%, and 11% gave 0·01–0·09%. In 17% of the cases, colony formation was not observed. The PEs were not correlated with the degree of pigmentation or with the clinical course. The reason for the high PEs was shown to be the presence of rat erythrocytes and the low $O_2$ used. The data confirm that the soft-agar method provides good culture conditions for human melanoma cells.

The usefulness of the Courtenay colony-forming assay in predicting individual clinical responses to chemotherapy was examined. Evaluable chemosensitivity data *in vitro* were obtained on 104 metastases from 83 melanoma patients. For all 6 drugs studied the observed sensitivity *in vitro* ($1/ID_{50}$) was converted into the same *in vivo* unit, *viz.* the expected growth delay (EGD) by means of the calibration curves obtained on melanoma xenografts. A clear correlation was found between the *in vitro* chemosensitivity and the clinical response to chemotherapy. Thus, tumours from patients with partial response, mixed response or stable disease after prior progression, all had rather high *in vitro* sensitivity to the drug used (EGD > 2·0); whereas patients with progression had lower sensitivity. In a few cases, chemotherapy regimen was chosen on the basis of *in vitro* sensitivity, and objective clinical responses were obtained. The *in vitro* soft-agar assay here used seems promising in aiding clinicians in tailoring chemotherapy to individual patients.

**DIFFERENTIAL RESPONSES OF MURINE AND HUMAN-TUMOUR CELL LINES TO ANTI-TUMOUR DRUGS BEFORE AND AFTER EXPOSURE TO RADIATION.** A. S. Belammy & B. T. Hill, *Laboratory of Cellular Chemotherapy, Imperial Cancer Research Fund, London WC2A 3PX.*

The influence of several fractions of X-irradiation (DXR) on subsequent response to chemotherapeutic agents has been investigated in mammalian tumour-cell lines *in vitro*. Details of DXR-pretreatment of murine L5178Y lymphoma cells and a human line, HN-1, derived from a head and neck squamous-cell carcinoma have been described previously, together with a characterization of these sub-lines (Bellamy & Hill, 1981, *Br. J. Cancer*, in press). Survival of parent and DXR-treated tumour cells after 24h drug exposures was assayed by colony formation in soft agarose.

Results with L5178Y cells indicate greater sensitivity of DXR-pretreated cells to bleomycin, cis-platinum and dibromodulcitol than untreated cells: $IC_{10}$ values being decreased 4-, 2- and 2-fold respectively. Responses to VP-16-213 and vincristine were considerably reduced by DXR-pretreatment, with survival plateauing at 50 and 95% respectively. However, the cytotoxicity of adriamycin and 5-fluorouracil appears to be unchanged.

Preliminary results with the human HN-1 lines, indicate that DXR-treatment also causes changes in sensitivity to cis-platinum, bleomycin and VP-16-213. In contrast to results with the murine cells, 5-fluorouracil shows increased cytotoxicity on DXR-pretreated cells over untreated parent cells.

It is hoped that this information may be of value in designing more effective drug therapies for human tumours recurring after DXR, since several types, including head and neck tumours (Price & Hill, 1980, *J. Laryngol. Otol.*, **94,** 89) show a significantly reduced response to subsequent chemotherapy.

**THE SURVIVAL OF HUMAN MELANOMA CELLS TAKEN DIRECTLY FROM PATIENTS AND TREATED WITH LOW DOSES OF γ-RADIATION.** V. D. Courtenay & J. Mills, *Institute of Cancer Research, Sutton, Surrey*

Cell survival data from established cell lines of human melanoma irradiated *in vitro* (Barranco *et al.*, 1971, *Cancer Res.*, **31,** 830; Guichard *et al.*, 1977, *J. Natl Cancer Inst.*, **18,** 1967) have tended to show relatively large shoulders to the survival curves. Such results have provided a theoretical basis for the introduction of larger dose fractions in radiotherapy regimes for the treatment of melanomas.

Our objective in the present studies was to take melanoma cells directly from patients and to measure their response to radiation at doses in the shoulder region of the dose/response curve. The dose range up to 7 Gy covered the range of dose likely to be given as a single fraction in fractionated radiotherapy. Single-cell suspensions from the disaggregated tumours were exposed *in vitro* to the γ-radiation from a $^{60}Co$ source under oxic conditions. Cell survival was assayed in a replenishable soft-agar colony technique (Courtenay & Mills, 1978, *Br. J. Cancer*, **37,** 261). Colonies of more than 50 cells were counted and PEs of 2–10% were obtained. Survival curves showed little difference between 1 tumour and another and exhibited a characteristic shape continuously bending from an initial slope. The curve was best fitted by the linear quadratic equation $\ln f = -(\alpha D + \beta D^2)$. These melanomas were also maintained as xenografts in CBA mice or cultured for several weeks as monolayers. Over the same dose range, cells from xenografts or cultures gave curves indistinguishable from those from the original tumours.

These results suggest that a wide shoulder to the survival curve is a common and stable characteristic of human melanoma cells.

**PROLIFERATIVE RESPONSES OF HUMAN PROSTATIC NEOPLASIA AND RAT PROSTATE IN ORGAN CULTURE.** A. C. Riches, D. Mistry, L. Buchanan, G. Dattani & J. P. A. Weaver, *Department of Anatomy and Experimental Pathology, University of St Andrews, and Department of Urology, Dundee Royal Infirmary*

In an attempt to provide a model for investigating hormone responsiveness and sensitivity to chemotherapy of prostatic neoplasia, the proliferative responses of human and rat prostate have been followed in organ culture using $^{125}$IdU uptake to monitor DNA synthesis. In serum free cultures, testosterone induced a marked increase in DNA synthesis in prostates from 4–6-month-old rats, reaching maximal levels after 4 days in culture, after which the uptake decreases to control unstimulated levels. This proliferative response is dose dependent and is maximal at testosterone concentrations of $4 \times 10^{-9}$ M to $4 \times 10^{-6}$M, whereas in prostates from 12-month-old rats the response was less marked. Human benign prostatic hyperplasia showed a minimal response to testosterone, a similar pattern to that seen in the older rats. The proliferative response of human benign prostatic hyperplasia increases up to Day 3–4 in culture, and then declines in both control and hormone-treated groups, and may represent repair processes which appear to be hormone dependent. Explants from prostatic carcinoma were well maintained in organ culture, but had to be carefully selected using frozen sections to obtain cellular areas in the transurethrally resected tissue samples. No marked changes in the histological appearance of the prostatic-carcinoma explants was found in the presence of testosterone, Estracyt or oestradiol. Testosterone exhibited some stimulatory effects in $^{125}$IdU uptake, whereas both Estracyt (oestramusine phosphate) and estradiol were weakly inhibitory. Thus, explants of prostatic neoplasia are well maintained in organ culture and provide a model for investigating the effects of therapy.

**HUMAN BLADDER TUMOURS IN SHORT-TERM ORGAN CULTURE.** A. H. Lawson, A. C. Riches & J. P. A. Weaver, *Department of Anatomy and Experimental Pathology, University of St Andrews and Division of Urology, Dundee Royal Infirmary*

Transitional cell tumours of the urinary bladder present a problem in management because of their tendency to recur. The tumours may be completely cured after resection, may recur at the same grade and stage or may recur at a worsening grade and stage. There is no way of predicting behaviour on histological appearance alone. Treatment may be by resection, radiotherapy, total cystectomy, intravesical chemotherapy or systemic chemotherapy. Diffuse papillary carcinomas are particularly difficult to treat by resection, but total cystectomy or radiotherapy carries a high morbidity for a low-grade tumour. These may be treated by instilling anti-cancer agents into the bladder. Human bladder tumours have been maintained in organ culture and their kinetic properties and responses to anti-cancer agents *in vitro* have been studied. Proliferative behaviour is studied using $^{125}$IdU uptake in combination with histology, vincristine metaphase arrest and autoradiography with [$^3$H]dT. Drug treatment is mimicked by immersing the explants for 1 h, washing and then studying recovery. Different behaviour is seen in different grades of tumour, and different tumours respond differently to different drugs. Correlation between *in vitro* and *in vivo* behaviour remains to be established. If a method of identifying tumours liable to rapidly progress could be established, it would enable urologists to embark on much more radical treatment at an early stage of the disease. Similarly a method of testing tumours for drug sensitivity would enable one to tailor treatment for each individual patient.

**GROWTH OF HUMAN BLADDER-CANCER BIOPSIES IN IMMUNE-DEPRIVED MICE.** J. H. Hay*‡, A. Busuttil†, C. M. Steel‡ & W. Duncan*, **University Department of Clinical Oncology, ‡Department of Pathology, and ‡MCR Clinical and Population Cytogenetics Unit, Western General Hospital, Edinburgh*

A series of serially transplantable xenografts has been successfully established from biopsies of transitional-cell carcinoma of the bladder. These are now being used for chemotherapeutic and radiobiologial studies.

CBA/Lac mice were prepared by thymectomy and whole-body irradiation after pretreatment with 200 mg/kg of Arg-C (Millar *et al.*, 1978, *Cell Tissue Kinet*, **11**, 543). The mice received 7·5 Gy of 250 kV X-rays, which is the $LD_{50}$ of untreated mice from our colony. All biopsy material has been collected from primary bladder tumours; no metastatic tumour has been used.

To date, a total of 4/33 transitional-cell carcinoma biopsies have taken and successfully passaged. 0/8 well differentiated (G1), 2/12 moderately differentiated (G2) and 2/13

poorly differentiated (G3) tumours have taken. One squamous carcinoma and one mixed transitional-cell and adenocarcinoma have failed to grow. 3/4 successful takes have been from tumours recurring after surgical resection, the 4th was from a previously untreated patient. 3/4 of the xenografts grow subcutaneously as spherical tumour nodules, the 4th maintains the sessile pattern commonly seen in the bladder. The histological appearances of all the xenografts are similar to those of their parent tumours. No successful takes have occurred from biopsies taken when the bladder was irrigated with water or from biopsies taken with the resectoscope.

### CORRELATION OF ANTITUMOUR ACTIVITY OF HEXAMETHYLMELAMINE ANALOGUES WITH THEIR *IN VITRO* CYTOTOXICITY AND METABOLISM.

S. P. Langdon, D. Ross, A. Gescher, J. A. Hickman & M. F. G. Stevens. *C.R.C. Experimental Chemotherapy Group, Department of Pharmacy, University of Aston, Birmingham B4 7ET*

The degree of antitumour activity of HMM and its analogues in mice correlates with the extent of their *in vitro* biotansformation to formaldehyde (F) and formaldehyde precursors (FP) (Rutty, C. J. *et al.*, 1977, *Biochem. Pharmacol.*, **26**, 2385). In an attempt to explain the exceptions to this correlation (e.g. the chloro analogue of HMM, IV) we investigated the *in vitro* cytotoxicity and metabolism of 3 melamine analogues (I, II, III) which have shown activity and three compounds (IV, V, VI) devoid of activity.

These results suggest that *in vitro* cytotoxicity correlates with *in vitro* biotransformation, whilst the antitumour activity of these analogues correlates with plasma levels of FP generated by metabolism.

### METABOLISM OF MONOMETHYLTRIAZENES.

J. K. Horton, P. Farina*, A. Gescher, J. A. Hickman & M. F. G. Stevens. *CRC Experimental Chemotherapy Group, University of Aston, Birmingham B4 7ET, and *Istituto Mario Negri, Milan, Italy*

(I) R = Me, $R^1$ = Ac
(II) R = H, $R^1$ = Ac
(III) R = H, $R^1$ = CN
(IV) R = H, $R^1$ = $NO_2$

Antitumour 1-aryl-3,3-dimethyltriazenes (DMT) show cytotoxic activity *in vitro* to TLX5 lymphoma cells only after metabolic activation. Products of their demethylation (the monomethyltriazenes MMT) have been posulated to be the active antitumour species by a number of workers, but we have questioned this because MMTs were found to be equitoxic *in vitro* to TLX5 cells sensitive or resistant to DMTs *in vivo* (Gescher *et al.*, 1981, *Biochem. Pharmacol.*, **30**, 89). Under conditions where DMTs are known to be activated *in vitro* (*ibid*) the DMTs (I) (500 μg/ml) produced < 10 μg/ml of (II) as measured by HPLC. This was below the level necessary to account for the cytotoxicity and it was considered possible that either MMTs are activated further, or they are not relevant to cytotoxicity. Under these activation conditions (9000 g fraction of mouse liver homogenate and cofactors) 90% of the MMT (II)

| R | $R^1$ | Peak FP levels in plasma (μM)* | Cytotoxicity *in vitro* (% inhibn.†) − | + | Biotransformation *in vitro*‡ |
|---|---|---|---|---|---|
| I $NMe_2$ | $NMe_2$ | 93 | 0 | 97 | 100 |
| II NHMe | $NMe_2$ | 243 | 0 | 99 | 98 |
| III $NH_2$ | $NMe_2$ | 101 | 0 | 60 | 38 |
| IV Cl | $NMe_2$ | 16 | 63 | 96 | 113 |
| V $NHNH_2$ | $NMe_2$ | 33 | 24 | 11 | 13 |
| VI NHMe | NHMe | 51 | 0 | 3 | 6 |

* After i.p. administration of 0·48 mmol/kg drug. Method of Sawicki *et al.*, 1961, *Anal. Chem.*, **33**, 93), adapted to distinguish between F and FP.

† 2h incubation of $10^6$ PC6A cells with 5 mM drug in the absence (−) or presence (+) of a liver microsomal activating system (LMAS).

‡ Metabolism on incubation with LMAS as measured by the appearance of F and FP according to Nash, 1953, *Biochem. J.*, **55**, 416); % relative to HMM.

(4 μg/ml) was shown by HPLC to have disappeared in 30 min, whereas <30% disappeared in controls (e.g. boiled liver). Similar results were obtained when the MMT (III) and (IV) were used. An *in vitro* assay of cytotoxicity of the MMT (II) ± liver fraction and cofactors, suggested that metabolism of this MMT is a deactivating process and thus that some other metabolite of DMTs is likely to be responsible for their antitumour effect.

**PHARMACOKINETIC STUDIES OF N-METHYLFORMAMIDE (NSC 3051) IN MICE.** C. Brindley, A. Gescher, E. S. Harpur & J. A. Slack, *CRC Experimental Cancer Chemotherapy Group, University of Aston, Birmingham B4 7ET.*

N-Methylformamide (NMF) is an agent with antitumour activity against a number of murine tumours, and is currently undergoing Phase I clinical evaluation. As part of a preclinical investigation of its pharmacological properties, its pharmacokinetics in mice were studied. 400 or 80 mg/kg NMF was administered i.v., i.p. or orally to male CBA CA mice (20–25 g). When these doses of NMF were given repeatedly to mice tumour growth was inhibited (Langdon *et al.*, 1981, *Br. J. Cancer*, **44**, 277). Blood samples (20 μl) were taken at intervals from the tip of the tail for 24 h after drug administration. The plasma concentration of unchanged NMF was measured by gas–liquid chromatography. After i.v. administration, the plasma concentration *vs* time curves (AUC) were 5325 ± 1270 μg.h/ml (n = 5) for the high dose and 1399 ± 276 μg.h/ml (n = 4) for the low dose. The corresponding AUCs for oral administration were 5360 ± 1117 μg.h/ml (n = 8) and 1058 ± 201 μg.h/ml (n = 3). After i.p. administration, the route used in the antitumour tests, 400 mg/kg NMF gave a AUC of 5452 ± 783 μg.h/ml (n = 5). These AUCs show that the systemic availability of NMF in mice after i.p. or oral administration was equivalent to that after i.v. administration.

The disappearance of NMF from plasma, at least at high concentrations, appeared to follow zero-order kinetics. Our current investigations of the disposition of NMF may provide an explanation for its apparently non-linear pharmacokinetic behaviour.

**THE PHARMACOKINETICS OF ORAL AND IV TREOSULFAN.** J. Welsh*, J. F. B. Stuart*‡, M. Soukop†, D. Cunningham†, R. Blackie*, G. Sangster*, & K. C. Calman, **Department of Clinical Oncology, University of Glasgow*, *†Department of Medicine, Royal Infirmary, Glasgow*, *‡Department of Pharmacy, University of Strathclyde, Glasgow*

Treosulfan (L-threitol 1,4-bismethanesulphonate) is itself inactive. Its conversion to the active epoxides is not enzymatically dependent, the transformation being dependent on temperature and pH; this has been shown *in vitro* (Feit, 1970, *J. Med. Chem.*, **13**, 1173). Treosulfan is conventionally prescribed at low doses e.g. 500 mg b.d. for 2–4 weeks followed by a rest period of 2–4 weeks dependent on haematological parameters. The chief role of treosulfan is in the treatment of ovarian cancer. Treosulfan has been shown to have a short half-life in animal studies. This alkylating agent may thus be suitable for high dose regimens involving autologous marrow replacement. This study was designed to determine the pharmacokinetics and haematological toxicity of moderate dose treosulfan (200 mg/kg). Seven patients were studied, 4 of whom received the drug i.v. and orally, the other 3 receiving the drug i.v. only. Samples of plasma, tears, saliva, urine and in one case bile, were obtained and immediately frozen at −20°C until analysis. The analytical technique was a gas–liquid chromatographic method. The bioavailability of oral treosulfan approaches that of i.v. treosulfan, and tear and saliva measurements closely follow plasma levels. Treosulfan was also detected in bile and urine. Peak plasma levels of 0·7 mg/ml were obtained and the $t\frac{1}{2}$ for oral treosulfan was ~1·8 h, compared to a $t\frac{1}{2}$ of 1·5 h for i.v. drug. In conclusion, it would appear that treosulfan in the dose of 200 mg/kg is reasonably well tolerated, and that its kinetics suggest that it would be suitable for use in high dose in association with autologous marrow replacement.

**PHARMACOKINETIC AND TOXICITY STUDIES WITH CB 3717.** D. R. Newell, Z. H. Siddik, K. G. McGhee, Ann L. Jackman, A. H. Clavert & K. R. Harrap. *Dept Biochem. Pharmacol., Inst. Cancer Res., Sutton, Surrey*

CB 3717 (N - (4 - (N - ((2 - amino - 4 - hydroxy-6 - quinazolinyl)methyl)prop - 2 - ynylamino)-benzoyl)-L-glutamic acid) is a folate-based inhibitor of thymidylate synthetase (TS) currently undergoing clinical trial. Pharmacokinetic studies have been made in mice at 100 mg/kg i.p., a dose which is curative against the L1210 leukaemia on a daily $\times 5$ schedule. This dose produced peak plasma levels ($71 \pm 8$ $\mu$g/ml) of CB 3717, achieved within 2 h. This concentration was maintained for a further 2 h after which levels decayed with a half-life of $81 \pm 8$ min. Within 24 h $55 \pm 11\%$ of the administered dose was present in the faeces and $14 \pm 1\%$ excreted in the urine. No CB 3717 metabolites have been detected significant activity against TS. In tissue distribution studies, $6{\cdot}5 \pm 1{\cdot}2\%$ of the administered dose was present as the unchanged drug in the kidney 24 h later. With repeated daily administration this is associated with an increase in kidney wet weight, whilst at lethal doses (300 mg/kg/day) animals died from renal failure. Co-administration of $NaHCO_3$ (2·1 g/kg/day) prevents the precipitation of CB 3717 in the kidney and the associated increase in kidney wet weight. The presence of CB 3717 in the faeces is suggestive of biliary excretion. This was confirmed in the rat, where at low doses (1–10 mg/kg i.v.) $>70\%$ of the dose was excreted unchanged in the bile within 4 h. At a higher dose (40 mg/kg i.v.) CB 3717 precipitation in the bile duct and a reduced bile flow was observed. This effect consistently occurred when plasma CB 3717 levels exceeded 10 $\mu$g/ml. These studies indicate that renal and hepatic toxicities may be anticipated in man. However, with appropriate scheduling, these side-effects can be avoided.

**EARLY CLINICAL STUDIES WITH THE QUINAZOLINE CB 3717.** DAWN L. ALISON, D. R. NEWELL, A. H. CALVERT & K. R. HARRAP, *Dept Biochem. Pharmacol., Inst. Cancer Res. and Royal Marsden Hosp., Sutton, Surrey*

CB 3717 is a cytotoxic antifolate currently undergoing early clinical trials. Two dose schedules are being used involving 1h infusions (single dose or daily $\times 5$) repeated at 3-week intervals. 24 patients have undergone treatment so far. Renal toxicity, which might have been anticipated from the preclinical studies, has not been seen. Several patients, however, have displayed abnormalities of hepatic function, with raised transaminase levels. This followed plasma levels of CB 3717 which would be expected to cause biliary precipitation of the drug in rats. It has been possible to overcome the liver disturbance in 2 patients by infusing the same dose of drug over 12–24 h. A variety of rashes has been seen in 6 of the patients, one displaying a diffuse erythematous reaction at the sites of previous irradiation. Marrow suppression has occurred in 4 patients, with neutropoenia and thrombocytopoenia developing 10–12 days after starting the 5-day treatment. Pharmacokinetic studies have shown that the renal excretion of CB 3717 varies 10–80%. Results suggest that the maximum tolerated dose will be in the range 140–200 mg/m$^2$/day for the 5-day schedule, achieving plasma levels above 10 $\mu$g/ml is the cytotoxic level *in vitro*. With the single-dose schedule 330 mg/m$^2$ has been tolerated in 3 patients so far, one of whom has attained peak plasma levels of 54 $\mu$g/ml and has also had an objective response to treatment.

**THE PHARMACOKINETICS OF S.C. INFUSIONS OF AraC.** M. L. SLEVIN, E. M. PIALL†, G. W. AHERNE†, A. JOHNSTON‡ & T. A. LISTER*, **ICRF Dept of Medical Oncology, St Bartholomew's Hospital, London, Dept of Biochemistry. University of Surrey, Guildford, ‡Dept of Clinical Pharmacology, St Bartholomew's Hospital, London*

The therapeutic advantages of continuous infusion of AraC have to be balanced against the disadvantages of hospitalization and the medical and nursing supervision required to establish and maintain i.v. therapy. S.c. bolus AraC has been used as an alternative to i.v. infusion. However, s.c. bolus AraC is rapidly absorbed and then declines with a half life similar to that of i.v. AraC, and within a few hours plasma AraC levels are $\pm 10\%$ of steady state infusion levels (Slevin *et al.*, 1981, *BJCP*, **12**, 507). This within-patient study compared the pharmacokinetics of s.c. infusions of AraC (100 mg/m$^2$ over 12 h) with the same dose given by continuous i.v. infusion in 6 patients with acute myelogenous leukaemia. The mean plasma concentration of AraC reached a plateau within 2 h, and the plasma concentrations and the area under the curve (AUC) were similar for both methods of administration. The mean AUC was $1147 \pm 230$ for the i.v. infusions, and $1017 \pm 238$ ng/h/ml for the s.c. infusions. There was a relatively large inter-patient difference in the plasma levels during the infusions. Furthermore, there was also a >2-fold variation in the plasma concentrations of AraC within individual patients after the plateau had apparently been reached. The variable plasma concentrations during the so-called "steady state" need to be taken into account in any attempt to correlate steady-state AraC levels with therapeutic outcome. This study demonstrates that s.c. infusion of AraC is a feasible alternative, and comparable to i.v. infusion. It may allow the patient the benefit of out-patient therapy while preventing unnecessary thrombophlebitis.

### THE COMBINATION OF CYTOTOXIC DRUGS WITH CLINICALLY RELEVANT DOSE REGIMES OF MISONIDAZOLE.

P. R. Twentyman & P. Workman, *MRC Clinical Oncology and Radiotherapeutics Unit, Cambridge*

A number of groups, including our own, have demonstrated that the effectiveness of various cytotoxic drugs against mouse tumours may be enhanced by the addition of the nitroimidazole radiosensitizer, MISO to the drug treatment. In some, but not all, of these studies a therapeutic gain is claimed, in that enhancement of tumour response is greater than the increase in normal tissue toxicity. Almost all of these investigations have used large single doses of MISO in the range 2·5–5 mmol/kg, which produce plasma levels 5–10 × those seen after the maximum clinical dose of the drug (3 g/m$^2$). The half-life of MISO is, however, much longer in man than in the mouse, and this factor may compensate for the reduced peak levels.

We have now carried out a series of experiments in mice in which repeated injections of MISO at 30 min intervals have maintained plasma levels at 100 μg/ml for either 7 or 16 h. In C3H mice bearing the KHT sarcoma, the tumour response to the nitrosourea CCNU was enhanced only nominally when the CCNU was administered at 4 h into the 7h regime. The enhancement was greatest at lower doses of CCNU, and was less than that for a large single dose of MISO. A similar regime of repeated MISO administeration produced little if any increase in the response of the RIF-1 sarcoma to cyclophosphamide (CY) and chlorambucil, in melphalan. Data for the 16h regime of MISO administration indicate greater enhancement of CY response than seen for the 7h regime.

### STUDIES ON THE FORMATION OF AN AFLATOXIN $B_1$-8,9-OXIDE CONJUGATE WITH GLUTATHIONE $AFB_1$-GSH *IN VITRO* AND *IN VIVO*.

R. C. Garner, P. J. Hertzog & J. R. Lindsay-Smith, *Cancer Research Unit, University of York, Heslington, York YO1 5DD*

Incubation of aflatoxin $B_1$ ($AFB_1$) and ($^{14}C$)-labelled glutathione with either a rat or mouse liver post-mitochondrial supernatant (PMS) and subsequent reversed-phase HPLC of the metabolites revealed the formation of an $AFB_1$-GSH conjugate. This metabolite eluted prior to other AFB: metabolites such as $AFM_1$ and $AFP_1$. Mouse PMS produced more of this metabolite than rat PMS. UV spectra of $AFB_1$-GSH indicated that linkage of GSH to $AFB_1$ was through a sulphur rather than a nitrogen group. Acid hydrolysis (N HCl at 100°C for 60 min) of $AFB_1$-GSH released the glutamic residue, to leave an $AFB_1$ conjugate that was not $AFB_1$-8, 9-diol. A metabolite with an identical retention time on reversed-phase chromatography to $AFB_1$-GSH was also found in the bile of cannulated rats administered $AFB_1$. Despite the formation of this GSH conjugate *in vitro* and *in vivo*, addition of GSH to the top-agar of a Salmonella/microsome assay of $AFB_1$ does not inhibit mutagenicity.

### MONOCLONAL ANTIBODIES TO AFLATOXIN-DNA ADDUCTS.

P. J. Hertzog, J. Lindsay-Smith & R. C. Garner, *Cancer Research Unit, University of York, Heslington, York*

Aflatoxin $B_1$ is a probable human liver carcinogen found in high levels in certain countries. The potential of antibodies to aflatoxin $B_1$ DNA ($AFB_1$-DNA) adducts is important since[5] they would reflect not only exposure, but actual levels of damage, and permit, to some extent, the much needed study of carcinogenesis in humans.

We have raised such antibodies using the guanine open-ring (ro) form of $AFB_1$-reacted DNA (ro $AFB_1$-DNA) coupled to methylated keyhole-limpet haemocyanin as immunogen. Spleen cells from immunized mice were fused with myeloma cells by conventional methods and hybridomas selected in HAT medium. Specific antibody-secreting hybridomas were detected by enzyme-linked immunosorbent assay (ELISA) using microtitre plates with wells coated with ro $AFB_1$-DNA. Positive hybridomas were cloned twice by limiting dilution, then grown in ascites, for the production of high-titre ($1:10^5$) ascites fluid for use in immunoassays.

In standard competition assays, dilute ascites fluid was preincubated with or without an inhibitor (e.g. DNA, $AFB_1$-DNA), followed by incubation on the plate. The monoclonal antibodies produced reacted specifically with AFB:-DNA, rather than DNA, and could detect adducts at the levels found in liver DNA from animals dosed with aflatoxin $B_1$.

### USE OF MONOCLONAL ANTIBODIES TO INVESTIGATE THE CELLULAR EVENTS ASSOCIATED WITH HEPATOCARCINOGENESIS.

C. H. Holmes, B. Gunn, E. B. Austin & M. J. Embleton, *Cancer Research Campaign Labs., University of Nottingham, Nottingham NG7 2RD*

Monoclonal antibodies to rat hepatocytes were prepared by conventional techniques, involving fusion of hepatocyte-immune spleen cells from BALB/c mice with the mouse myeloma, P3-NS1. One of these antibodies, RL23/36, showed preferential reactivity with adult rat hepatocytes, while a second antibody, RL24/72, was much less specific and showed cross-reactivity with a number of other cell types. Both monoclonal antibodies were specific for hepatocytes within the liver, and showed no reactivity with hepatic sinusoidal cells. In addition, a third monoclonal antibody, RL24/106, which was produced in the same way, showed reactivity with connective-tissue components in both liver and other tissues.

The expression of both hepatocyte-expressed antigens is profoundly affected during hepatocarcinogenesis, the RL23/36-defined antigen especially being reduced in a number of dimethylamino-azobenzene (DAB)-induced hepatomas.

The cellular composition of a range of lesions induced in the liver by feeding dietary DAB was examined immunohistochemically, using these 3 monoclonal antibodies in conjunction with several commercially available monoclonal antibodies directed against rat lymphocytes. Cells within these lesions exhibited considerable heterogeneity with respect to both expression of antigens (identified by RL23/36 and RL24/72) and also the degree and type of infiltrating cells observed. Overall, there was a trend towards a loss of liver-associated antigens in hepatocyte-derived cells and, in some cases, an increase in the number of infiltrating host cells.

### THE ULTRASTRUCTURE OF DMBA-INDUCED DYSKERATOSIS.

F. H. White, R. M. Codd, N. J. Smith & K. Gohari*, *Departments of Human Biology and Anatomy and *Oral Pathology, University of Sheffield*

The development of malignancy in keratinized stratified squamous epithelia in both humans and experimental animals is accompanied by the development of a variety of histological changes known collectively as epithelial dysplasia. One of these features, dyskeratosis, is characterized by the production of keratin in abnormal sites, i.e. in areas other than on the surface of the tissue. The present report describes the ultrastructural features of dyskeratotic regions from lesions produced in the hamster cheek pouch epithelium by the chemical carcinogen DMBA. Following DMBA application to cheek pouches of male Syrian golden hamsters for a minimum of 7 weeks, tissue samples were removed and processed for electron microscopy. Many individual dyskeratotic cells within the spinous layer were seen. They possessed a central, often pyknotic, nucleus around which whorls of tonofibrils were present; between the tonofibrils, mitochondria in various stages of degeneration, vacuoles and lipid droplets were seen, and these were often densely packed around the nucleus. Keratin pearls consisted of a central whorl of either ortho- or parakeratinized cells around which granular cells were present; these often contained small round keratohyaline granules and abundant, often reticular, tonofibril networks. Our observations suggest that dyskeratosis begins with the keratinization of an individual cell or group of cells. Adjacent cells subsequently undergo flattening and whorling around this nidus until a keratin pearl is formed.

**BLOOD-VESSEL FREQUENCY DURING EXPERIMENTAL EPIDERMAL CARCINOGENESIS.** F. H. WHITE & B. AL-AZZAWI, *Department of Human Biology and Anatomy, University of Sheffield, Sheffield S10 2TN*

The vascularization of tumours seems to be essential for their continued growth and development, but there is relatively little information on the way the vessels develop in the vicinity of neoplasms. Using morphometric methods, we have previously reported progressive increases in the volume of blood vessels during experimental skin carcinogenesis. These methods enable the generation of objective data from histological sections. Estimates of volumetric alterations are limited, in that they do not provide a complete description of the changes. In the present report, we have estimated blood vessel frequency (number per unit section area) which will enable us to determine whether the changes are a result of blood-vessel dilatation or of new blood-vessel production.

Male Syrian golden hamsters were treated 3 times weekly with DMBA in liquid paraffin. Two groups of animals were used as controls, one of which was untreated and the other receiving topical applications of liquid paraffin for 10 weeks. Histological sections were prepared and, using an image analyser, the number of vessels per unit section area of dermis and hypodermis was quantified. There were progressive and significant increases in the frequency of blood vessels during skin carcinogenesis. In combination with our previous findings, the results indicate that in this experimental system, vascular alterations are produced by the generation of new blood vessels as well as by dilatation of existing ones.

**THE REGIONAL LYMPH NODE (RLN) RESPONSE TO CHEMICAL CARCINOGENESIS IN THE HAMSTER CHEEK POUCH (HCP).** G. T. CRAIG, *University of Sheffield, Department of Oral Pathology, Sheffield S10 2TA*

The prognostic significance attributable to morphological changes in RLN draining sites of human primary cancer is somewhat variable and often contradictory. Data obtained from a variety of experimental models are of questionable value, given the predilection for the use of transplanted tumours in heterotopic sites and the short durations studied. Few reports have documented the *in situ* RLN response to the development of induced primary carcinomas with proven metastatic potential. The present study used stereological (Craig, 1977, *J. Dent. Res.*, **56**, 116) and histochemical (Craig, 1980, *J. Dent. Res.*, **59**, 70) techniques to monitor the morphological response of RLN to DMBA carcinogenesis in the HCP over a 24 week period. The early response (0–2 wk), in both node and pouch, is consistent with the induction of contact hypersensitivity to DMBA; the associated and marked paracortical (PC) expansion persisted throughout the stages of carcinogenesis (i.e. hyperplasia, dysplasia, carcinoma). Coincident with the emergence of exophytic tumours (8–12 wk) the RLN showed prominent follicular cortical (FC) activity and plasma cell production. This pattern of FC proliferation was superimposed on the notably expanded but relatively inactive PC compartment during the period from 12–24 wk) The results suggest that the RLN response to DMBA carcinogenesis in the HCP is characterized by a sustained T-cell expansion, attributable initially to contact sensitization and subsequently to cell-mediated immunity against presumptively immunogenic exophytic tumours, and a later B-cell proliferation attributable either to non-specific ulceration of tumours or humoral immunity to shed tumour antigens.

### HISTOGENESIS OF BILE-DUCT CARCINOMA ARISING IN *OPISTHORCHIS VIVERRINI* INFECTED HAMSTERS GIVEN A SINGLE ORAL DOSE OF DIMETHYLNITROSAMINE.

D. J. Flavell, *Dept of Medical Helminthology, London School of Hygiene and Tropical Medicine, St Albans*

Intrahepatic bile-duct carcinoma appears to be associated with *Opisthorchis viverrini* infection in man (Sonakul *et al.*, 1978, *S.E. Asian J. Trop. Med. Pub. Hlth.*, **9**, 215). Long-term superinfections in hamsters for up to 20 months duration do not yield hepatic tumours (Favell, unpublished observations). Thamavit *et al.*, 1978, *Cancer Res.*, **38**, 4634) however, produced a 100% incidence of cholangiocarcinomas by administering 0·0025% DMN to *O. viverrini* infected hamsters. In this study, a dose of DMN expected to induce a low incidence of cholangiocarcinoma in hamsters was chosen. Control hamsters and hamsters receiving 50 *O. viverrini* metacercariae 40 days previously, were each given 1·6 mg DMN orally (Tomatis & Cefis, 1967, *Tumori*, **53**, 447). Results include observations on animals dying over 40 weeks after DMN treatment. Mortality was significantly higher in the group receiving both DMN and parasites. Intrahepatic cholangiocarcinomas were found only in animals receiving both parasites and DMN. All tumours were mucin secreting and goblet-cell metaplasia was a common feature. Tumours with an acinar type pattern contained an abundant connective-tissue stroma, and parasite egg granulomas were occasionally found in this stroma. Cholangiofibrosis and cystic cholangiomas were common in both groups, though cholangiofibrosis was more extensive in the group receiving both parasites and DMN. Cholangio-carcinomas appeared to arise either from hyperplastic processes in the bile-duct wall, or from areas of cholangiofibrosis distinct from major bile ducts. In both cases a range of transitional stages between hyperplasia and carcinoma were seen. Cholangiocarcinoma and cystic cholangioma were frequently seen in close proximity, and appeared to have their origin in adjacent areas of cholangiofibrosis.

### LIKELY MECHANISMS BY WHICH CARCINOGENS INDUCE ADRIAMYCIN RESISTANCE IN RAT HEPATOCYTES.

I. Carr & D. B. Langley, *Department of Medical Oncology, City of Hope National Medical Center, Duarte, California, 91910, U.S.A.*

Administration of the hepatocarcinogen 2-acetylaminofluorene (AAF) to rats induces a resistance in their hepatocytes to doses of adriamycin (Ad) that are cytocidal to normal hepatocytes after incubation *in vitro* in primary monolayer culture (Carr & Laishes 1981, *Br. J. Cancer*, **44**, 567). To investigate the mechanism of this carcinogen-induced resistance, sensitive (normal) and resistant (2-AAF altered) hepatocytes were incubated with Ad-$1{\cdot}8 \times 10^{-5}$M for periods up to 24 h. No differences were seen in the cellular uptake, efflux, DNA-binding, %DNA nicks, or % inactive Adraglycone metabolites between sensitive and resistant cells. However, only 50% of the fluorescing material was organic extractable. Examination of the aqueous non-extractable material revealed that this was 13% of the total cellular fluorescence in the sensitive cells and 34% in the resistant cells. It contained mainly highly polar metabolites, presumably water-soluble conjugates. Administration of Adr 5 mg/kg i.v. to normal or 2-AAF-treated rats produced in similar concentrations of Adr and % fluorescence in the non-toxic aglycone metabolites. No Adr OH was found. The differences in susceptibility to Adr induced toxicity presumably reside therefore in the generation of free radicals or in the ability to scavenge them, to produce excretable polar compounds.

### IS DYEING OF BAIT A CARCINOGENIC HAZARD FOR ANGLERS AND THEIR SUPPLIERS?

C. E. Searle & J. Teale, *Cancer Research Campaign Laboratories, Department of Cancer Studies, The Medical School, Birmingham B15 2TJ*

Chrysoidine (2,4-diaminoazobenzene), once used as a food dye, induced liver adenomas or carcinomas in most male and female C57BL mice when fed at 0·2% in the diet (Albert, 1956, *Arch. Immunol. Terap. Dosw.*, **4**, 189), and it was the most active of azo dyes tested for mutagenicity in *Salmonella typhimurium* TA 1538 (Garner & Nutman, 1977, *Mutat. Res.*, **44,** 9). Nevertheless it is currently in widespread use for dyeing maggots for use as bait by anglers, for which purpose the hydrochloride is commonly mixed with maggots by anglers' suppliers before sale. This practice causes considerable long-lasting staining of the hands of shop staff and of the very many anglers who use the bait, often from an early age.

A Midlands dealer, who has used 14 kg of chrysoidine HCl annually, became anxious about its safety and informed us of 3 angling acquaintances who had developed bladder tumours. The early ages of presentation (each was under 40) would suggest a possible "occupational" origin, but none had worked in the chemical or rubber industries.

Some other dyes, including the carcinogen auramine, have also been used, but apparently with lesser problems of contamination. There is a clear need for epidemiological studies to determine whether chrysoidine or other bait dyes might be hitherto unrecognized human carcinogenic hazards. Meanwhile it would appear prudent to discourage use of these suspect dyes under conditions causing contamination.

### FOLLICULAR CENTRE CELL HISTOLOGY IN CLASSICAL CHRONIC LYMPHATIC LEUKAEMIA.

D. I. Gozzard, A. Cadar, R. Cox, M. Light & M. J. Leyland, *Department of Clinical Haematology, East Birmingham Hospital, Bordesley, Birmingham B9.*

Lymph-node and/or marrow histology has been studied in 33 cases of CLL. The diagnostic criteria were those of the MRC CLL trial, and all had a monoclonal B-cell lymphocytosis and mouse red-cell rosetting. The mean presenting lymphocyte count was $111 \times 10^9$/l, with a range from $12–856 \times 10^9$/l and 2 $\mu$ plastic-embedded sections were used both for the marrow and lymph glands.

Of the 13 cases in which lymph gland biopsies were obtained, 4 had centrocytic follicular-centre cell histology, which was also demonstrable on the 2 $\mu$ marrow sections. These 4 cases had presenting white-cell counts $> 100 \times 10^9$/l, and in all other respects were classical CLL.

These data suggest that the initial white-cell count does not discriminate between classical CLL and follicular-centre cell non-Hodgkin's lymphoma with a monoclonal B-cell lymphocytosis. It is suggested that 2 $\mu$ histological sections of either marrow or lymph-node biopsies are necessary to determine the correct diagnosis.

This recognition of 2 distinct histopathological sub-groups with the same clinical syndrome may have prognostic significance.

### A STUDY OF BLOOD-GROUP ISOANTIGEN EXPRESSION ON NORMAL AND MALIGNANT GASTRIC EPITHELIUM, USING MONOCLONAL ANTIBODIES.

P. J. Finan*, S. H. Sacks†, E. S. Lennox† & N. M. Bleehen*, **MRC Clinical Oncology Unit and* †*MRC Laboratory of Molecular Biology, Cambridge*

It has been suggested that partial or complete loss of blood-group isoantigen (BGI) expression accompanies malignant change in gastric epithelium. Previous work has depended on conventional antisera, used in both immunofluorescent and specific red-cell-adherence techniques (Davidsohn *et al.*, 1966, *Arch. Pathol.*, **81,** 381; Sheahan *et al.*, 1971, *Am. J. Dig. Dis.*, **16,** 961). The availability of monoclonal antibodies (McAbs) to the blood groups A and B (Voak *et al.*, 1980, *Vox. Sang.*, **39,** 134; Sacks & Lennox, 1981, *Vox Sang.*, **40,** 99) has allowed a re-investigation of these findings, and an evaluation of mucosal BGI loss as a marker of malignant change in gastric epithelium.

Using an indirect immunoperoxidase technique on paraffin sections of normal gastric biopsies, the expected BGI was present in all 11 cases. In a series of 17 gastric cancers the BGI was lost in 6 (35%), though present in adjacent uninvolved mucosa. This loss was unrelated to histological grade, secretor status or blood subgrouping. In 6 cases, where lymph-node metastases were examined, the BGI status was the same as in the primary tumour.

It is concluded that McAbs to the blood group antigens A and B, with their specificity and homogeneity, may be usefully introduced into studies on BGI expression in malignant tissues. However, although loss of isoantigen can be demonstrated in some specimens of malignant gastric epithelium, this loss is unlikely to provide a reliable marker of malignant change.

**CYCLIC AMP-BINDING PROTEINS, OESTROGEN RECEPTORS AND PROGESTERONE RECEPTORS IN HUMAN BREAST CANCER.** D. M. A. WATSON, W. R. MILLER, R. A. HAWKINS, R. O. SENBANJO, J. TELFORD & A. P. M. FORREST, *Department of Clinical Surgery, University of Edinburgh*

Whilst human breast cancers with oestrogen receptor (ER) and progesterone receptor (PgR) activities are likely to be hormone responsive, many do not respond to endocrine therapy. In hormone-dependent rat mammary tumours the ratio of cyclic AMP-binding proteins to steroid receptors may better discriminate hormone-dependent from independent tumours than steroid receptors alone.

The inter-relationships between cyclic AMP-binding protein, ER and PR activities have therefore been determined in 100 human breast cancers. Cyclic AMP binding was detected in all tumours, ER in 70 and PR in 38. Mean cyclic AMP binding in $ER^-$ tumours was significantly higher than in the $ER^+$ group. ($P < 0{\cdot}025$ by $t$ testing). In tumours with both activities, there was no significant correlation between levels of binding ($P < 0{\cdot}1$ by Spearman rank regression analysis). Similar relationships were also found between cyclic AMP binding and PR. Expressing results as ratios of ER to cyclic AMP-binding proteins, neither divided the tumours into obvious subgroups nor discriminated between $PR^+$ and $PR^-$ tumours. The lack of strong correlations with either ER or PR means that it will require clinical follow-up to determine whether cyclic AMP-binding proteins may predict hormonal sensitivity in human breast cancer.

**BINDING OF WHEAT GERM-LECTIN TO RAT SARCOMAS OF DIFFERING. METASTATIC CAPACITY.** N. WILMOTT, S. A. SIMPSON & K. C. CALMAN, *Department of Oncology, University of Glasgow*

The reaction of WGA with the tumour cell surface (as expressed by amount bound, agglutination, resistance to toxic effects) has been reported to correlate with metastatic potential in a number of parisophanous tumour cells lines (i.e. tumours derived from a parental line). We are currently examining a range of rat sarcomas of independent origin to see whether there is any correlation between binding of WGA and metastatic capacity. The assay used to assess binding was based on the reaction of radiolabelled lectin to tumour cells in suspension, and results are expressed as a percentage of an internal standard. Inhibition studies showed that WGA bound to N-acetyl glucosamine and also N-acetyl neuraminic acid. Tumour-cell suspensions were prepared either by detachment of cells grown as monolayers *in vitro* or by mechanical disruption of a solid tumour grown *in vivo*, followed by removal of dead cells and debris by Ficoll-Paque, to avoid the use of enzymes. It was found that whilst *in vitro* cultured cells from tumours of different metastatic capacity showed no differences in lectin binding, differences were apparent using cells from mechanically disrupted solid tumours. It is concluded that transplantable animal tumours of independent origin may exhibit a correlation between metastatic capacity and binding of WGA, as do parisophanous tumour cell lines. However, we have yet to assess the influence of host-cell infiltrate in cell suspensions derived from solid tumours.

**THE LOSS OF CERTAIN WGA-BINDING PROTEINS IN RELATIONSHIP TO METASTASIS AND TO THE SITE** W-S. CHAN, A. W. JACKSON & G. A. TURNER *Department of Clinical Biochemical and Metabolic Medicine, Royal Victoria Infirmary, Newcastle upon Tyne NE1 4LP*

Recent studies (Guy *et al.*, 1980, *Br. J. Cancer*, **42,** 915; Reading *et al.*, 1980, *Proc. Natl Acad. Sci.*, **77,** 5943) have suggested that membrane protein glycosylation is important in relationship to metastasis. We have further investigated this possibility by determining the binding of $^{125}$I-WGA to electrophoretically-separated proteins in Triton X100 extracts from primary (1°) and secondary (2°) tumour cells of a metastasizing hamster lymphosarcoma. It was shown that WGA binds to a number of proteins in extracts from primary tumours growing in the subcutaneous site. However, in extracted 2° cells that were isolated from the liver and lymph nodes, the degree of WGA binding was consistently reduced. Control studies showed that the observed changes were not due to contamination, or proteolytic digestion of the WGA-binding proteins by host cells. The lost WGA-binding bands reappeared if 2° tumour cells, that had previously grown in the liver, were then grown in the s.c. site. Later studies, on tumour transplanted into a number of different 1° sites, indicated that the degree of WGA-binding varied according to the particular site investigated. These latter findings suggest that the observed changes are due to site-induced modulations rather than the selection of metastatic subpopulations.

**CORRELATION OF COLLAGENASE SECRETION WITH METASTATIC COLONIZATION POTENTIAL IN NATURALLY OCCURRING MURINE MAMMARY TUMOURS.** D. TARIN, B. J. HOYT, D. J. EVANS & R. C. YDENBERG, *Departments of Histopathology, University of Oxford, John Radcliffe Hospital, Headington, Oxford, and Royal Postgrad. Med. School, Hammersmith Hospital, London*

In this communication we report evidence of the secretion of a true mammalian collagenase active against Type I collagen by naturally-occurring mammary tumours of the mouse and show that tumours capable of heavily colonizing the lungs secrete significantly more of this enzyme than those with low pulmonary colonization potential or more non-neoplastic proliferating (e.g. lactating) mammary tissue. Plasminogen activator was secreted in greater quantity by tumours than by normal tissues but there was no significant difference in the amount produced by tumours with high or low pulmonary colonization potentials.

These findings correlate well with our earlier morphological observations of marked connective-tissue destruction in the vicinity of invading tumours and metastatic deposits, and indicate that protease release is implicated in the mechanism of tumour spread.

**METASTATIC PROPERTIES AND CELL-SURFACE BIOCHEMISTRY OF CLONES ISOLATED FROM AN HSV-2 TRANSFORMED HAMSTER CELL LINE.** J. R. WALKER, R. G. REES & C. W. POTTER, *Clinical Research Laboratory, Weston Park Hospital, Sheffield and Dept Virology, University of Sheffield*

After resection of s.c. tumours derived from the inoculation of the HSV-2-333-2-26 cell line into Syrian golden hamsters, a small proportion of animals developed metastatic lesions at secondary sites. Selection of variants by s.c. re-implantation of lung foci has produced 2 sublines, with a markedly greater metastatic potential than the parent line (Walker *et al.*, 1982, *Eur. J. Cancer* (in press)). In addition, numerous differences in cell surface glycoproteins labelled by the galactose oxidase-[$^3$H]sodium borohydride technique have been reported (Rees & Walker, 1981, *Br. J. Cancer*, **43**, 722).

The study of these cell lines has been extended by the repeated s.c. transplantation of lung and kidney foci, to produce lines of different organ propensity. The parent, poorly metastatic cell line has been cloned *in vitro*, to produce a range of clones of different malignancy. Preliminary studies have suggested that some cell-surface glycoprotein components also differ between the cell lines. Thus, it has been shown that cells of different metastatic potential exist in, and may be selected from, the HSV-2-333-2-26 cell line. The role of the surface glycoproteins in this heterogeneity is under investigation

**QUANTITATIVE ANALYSIS OF THE ARREST AND SUBSEQUENT FATE OF RIF-1 MOUSE SARCOMA SUBPOPULATIONS IN THE LININGS FOLLOWING INTRAVENOUS INJECTION.** J. G. REEVE & P. R. TWENTYMAN, *MRC Clinical Oncology and Radiotherapeutics Unit, Cambridge*

The RIF-1 tumour, an X-ray-induced sarcoma of the C3H/Km mouse, consist of subpopulations of cells which are heterogeneous with respect to a variety of malignant characteristics including lung-colony formation efficiency (LCFE). RIF-1 subpopulations of high LCFE have been isolated from the parent tumour by successive *in vivo* lung passaging and by *in vitro* cloning. To investigate cellular properties which affect metastatic ability we have examined the correlation between cell arrest and retention in the lungs and the formation of pulmonary lesions for 4 *in vivo* and 6 *in vitro* isolated RIF-1 subpopulations.

For lung arrest and retention studies $^{125}$IUdR-labelled cells were injected i.v. into syngeneic mice. The activity remaining in the lung 6 days post-injection was determined, and compared with the number of lung colonies visible 21 days post-injection. Although the LCFE of *in vivo* isolated subpopulations correlated well with cell retention by the lungs there was no clear correlation between the LCFEs of *in vitro* isolated RIF-1 clones and lung arrest and retention.

The fate post-injection of clone 16, which is retained well by the lungs but is a poor lung colonizer, was ascertained by using *in vitro* clonogenic capacity as a measure of cell viability in the lungs at various times post-i.v. injection of $10^5$ cells. Results show that clone 16 cells arrested in the lungs are viable for 26 days post-injection. We believe these cells to be in a state of dormancy, and are currently investigating the kinetic parameters of these apparently dormant cells.

### DIFFERENT RESULTS WITH TWO ASSAYS OF FIBRINOLYSIS AND THEIR CORRELATION WITH TUMORIGENICITY.

S. R. Fairbairn, R. Zammit-Pace & J. P. Roscoe, *School of Pathology, Middlesex Hospital Medical School, London*

We have studied plasminogen-dependent fibrinolysis in cells derived at different times after transplacental exposure to the carcinogen ethylnitrosourea (ENU) or buffer. The cells were tested for fibrinolytic activity by 2 main methods: (1) lysis in a fibrin-agarose overlay of colonies, or (2) release or radioactivity from a layer of $^3$H-fibrin seeded with whole cells. Cell lines which were tumorigenic immediately they were derived showed increased fibrinolytic activity in both assays when compared with control lines. However, some of these lines could lose their fibrinolytic activity as measured by the radioactive method, while remaining positive in the overlay assay, tumorigenic and able to form colonies in agar. Cell lines derived soon after ENU exposure also developed increased fibrinolytic activity (measured by the overlay method) and later could grow in agar or animals. However, latent period cultures showed low, slightly elevated or variable values of fibrinolysis in the radioactive assay. A comparison of the 2 assay methods suggested that differences in culture conditions, such as the time of medium change, may cause the difference in results. Fibrinolysis by some cells positive in the overlay assay and not in the radioactive assay, was reduced if the cells were incubated with fresh medium containing serum immediately before being overlaid. However, those cells which were positive in both assays were not affected by this treatment.

The difference in fibrinolytic activity of some cell lines in the 2 assays seems at least partly due to the difference in culture conditions. The development of tumorigenicity correlated well with increased fibrinolytic activity measured by the overlay assay, but less well with the radioactive assay.

### QUANTITATION OF MONOCLONAL ANTIBODY AND ANTIBODY-CONJUGATE BINDING TO TUMOUR CELLS USING FLOW CYTOFLUORIMETRY.

R. A. Robins, M. R. Price & R. W. Baldwin, *Cancer Research Campaign Laboratories, University of Nottingham, Nottingham NG7 2RD*

Monoclonal antibody directly conjugated with fluorescein isothiocyanate (FITC) has been used with a flow cytofluorimeter/sorter (Becton Dickinson FACS IV) to quantitate accurately antibody binding to tumour cells. Directly labelled 791T/36 monoclonal antibody (a mouse antibody against human osteogenic sarcoma cell line 791T) can be detected at a level of 100 fg per cell. Cells labelled with antibody can be analysed without washing, so that the kinetics of labelled antibody association and dissociation, and antibody bound at equilibrium, can be observed directly. The analysis of unlabelled antibody preparations, and antibody conjugates (e.g. drug-antibody, toxin-antibody) can also be performed rapidly and conveniently in competition assays; these methods will detect changes in affinity of conjugated antibodies. In assays using limiting quantities of labelled antibody, solubilized antigen may be detected readily.

**HUMAN HYBRIDOMAS FROM PATIENTS WITH MALIGNANT DISEASE.** K. Sikora, T. Alderson & J. Ellis, *Ludwig Institute for Cancer Research, MRC Centre, Hills Road, Cambridge*

Monoclonal antibodies provide unique tools to define and analyse the molecular components of tumour cell surfaces. There are many such antibodies of mouse or rat origin. Here we report the production of human monoclonal antibodies by the fusion of intratumoral or regional node lymphocytes from patients with a variety of malignant diseases, in the human myeloma line, LICR/LON/HMY2. Suspensions of lymphocytes were obtained either by disaggregating tumours or by teasing apart lymph nodes obtained at surgery. Fusion was carried out using polyethylene glycol and HAT medium used for selection. Material was obtained from 153 patients and, after processing, hybrids obtained in 22 of these. The total number of separate hybrids obtained exceeded 150. Hybridomas were analysed for DNA content using a flow cytometer and found to contain DNA equal to the sum of that present in the lymphocyte and the parent myeloma. This DNA content was stable after 8 months of continual culture, indicating the chromosomal stability of this hybrid system. The hybridoma supernatants contained human monoclonal immunoglobulins which were analysed using a sensitive solid-phase radioimmunoassay. Binding activity to tumour-cell surface components was detected in several hybrid supernatants, suggesting that B cells in the region of tumours may be involved in host defence.

**BIOLOGICAL ACTIVITY OF A MONOCLONAL ANTIBODY SPECIFIC FOR A RAT SARCOMA.** S. M. North & C. J. Dean, *Institute of Cancer Research, Clifton Avenue, Sutton, Surrey SM2 5PX*

A monoclonal antibody (M10/76) of IgG2 isotype, directed against a tumour associated antigen of the chemically induced rat fibrosarcoma, MC24, is being used to investigate the role of humoral immunity in metastatic disease.

To assess the biological activity of M10/76 *in vivo*, affinity-purified antibody was injected i.v. into immunocompetent rats immediately before an i.v. challenge with viable tumour cells. After 16 days the animals were sacrificed and the number of lung colonies estimated. Lungs from the control groups had large numbers of tumour colonies, whereas lungs from animals treated with specific antibody contained no visible tumour colonies.

Antibody M10/76 was also examined for its effects on spontaneous metastases *in vivo*. MC24 was grown in the hind limb of congenitally athymic male rats for 21 days and then removed by amputation. Specific antibody was administered i.v. at a dose of 50 μg/rat on alternate days throughout tumour growth, and until 75 days after amputation. The animals were then killed and examined for metastases. While all control animals showed metastases either to lung or lymph nodes, those treated with M10/76 showed either no metastatic lesions or enhanced metastatic disease.

We conclude that Ab M10/76 is biologically active *in vivo* and is capable of influencing the course of metastatic disease.

**THE EFFECT OF MONOCLONAL ANTIBODY-DIRECTED IMMUNOTOXINS AND IMMUNOMODULATORS ON CULTURED HUMAN OSTEOGENIC SARCOMA CELLS.** M. J. Embleton*, G. R. Flannery*, J. Pelham*, G. F. Rowland†, & R. W. Baldwin*, **Cancer Research Campaign Laboratories, University of Nottingham, and †Lilly Research Centre Ltd, Windlesham, Surrey*

A monoclonal antibody (MoAb) raised against a human osteogenic sarcoma cell line, 791T, was conjugated directly to various drugs and toxins, and to lymphoblastoid interferon (IFN, provided by Burroughs Wellcome Ltd). Conjugates were tested for cytotoxic properties on 791T and other cell lines using radioisotopic assays to measure protein or DNA synthesis.

Virtually all immunotoxin conjugates retained antibody-binding activity for 791T cells, but in most, cytotoxic activity was lost or diminished in comparison with the unconjugated agent. For example, a conjugate of Vindesine (VDS) and MoAb was 2000-fold less toxic than VDS alone on an $LD_{50}$ basis, when 791T cells were treated continuously for 24 h. However, when target cells were pre-exposed for 15 min and washed before culture, the conjugate was selectively toxic for 791T cells and other osteogenic sarcomas which bind the MoAb, but not for unrelated non-crossreactive cells.

IFN/MoAb conjugates did not directly affect the cells, but IFN alone had only a marginal cytostatic effect. However, IFN/MoAb was highly effective in stimulating NK cell activity against 791T and other target cells. 791T cells, but not antigenically unrelated cells, pre-incubated with the conjugate induced activation of NK cells against $^{51}$Cr-labelled third-party cells in mixed culture.

These studies suggest that with further development, immunotoxins may have specific anti-tumour properties, and that an alternative approach to therapy could be the use of indirectly acting MoAb-directed immunomodulators.

### NATURALLY CYTOTOXIC CELLS IN THE RAT: DO THE SAME CELLS LYSE SOLID AND LEUKAEMIC TARGETS?

G. R. Flannery, C. G. Brooks, J. D. Gray & R. W. Baldwin, *Cancer Research Campaign Laboratories, University of Nottingham, Nottingham NG7 2RD*

Most studies of natural cell-mediated immunity have used non-adherent lymphoid target cells, and only recently has it been recognized that cells derived from solid tumours may also be susceptible to such lysis. In the mouse it has been suggested that these functions are performed by 2 distinct effector cells. In the rat, our previous studies showed that sarcoma cells are lysed by effectors identical with the NK cell described by others using lymphoid targets. We report here that lysis of both target types is mediated by cells indistinguishable by a large variety of criteria.

A panel of solid and lymphoid targets from several species was tested in 6h $^{51}$Cr-release tests, using normal rat spleen cells as effectors. Both types of killer cell (a) exhibited a lag phase prior to steady-state lysis of some targets, (b) were absent from neonatal animals but present from 8 weeks to 18 months of age, (c) were non-adherent to nylon fibre, (d) were sensitive to anti-asialo GM1 serum and complement, (e) were enriched in low density fractions from discontinuous percoll gradients, (f) were augmented 2-fold by pre-incubation at 37°C (g) showed reduced activity in cross-competition tests in which each type of target cell was able to compete for lysis of the other.

These data, together with analysis of surface-antigen distribution defined by various monoclonal antibodies (see Cantrell *et al.*, below) strongly suggest that although NK-cell heterogeneity is found in the rat, as in other species, the cells which lyse solid and lymphoid targets are identical.

### NATURALLY CYTOTOXIC CELLS IN THE RAT: PHENOTYPE OF THE CELLS MEDIATING NATURAL CYTOTOXICITY AND A COMPARISON WITH CELLS MEDIATING ANTIBODY DEPENDENT CYTOTOXICITY.

D. A. Cantrell, R. A. Robins, C. G. Brooks & R. W. Baldwin. *Cancer Research Campaign Laboratories, University of Nottingham, Nottingham NG7 2RD*

The fluorescence-activated cell sorter (FACS) was used to separate rat spleen cells into subpopulations with and without the antigens defined by W3/13, W3/25, OX6 and OX8 monoclonal antibodies. The resultant populations were then assayed for NK and ADDC activity in a quantitative 6h $^{51}$Cr-release assay. The data establish that rat NK and ADCC effector cells are heterogeneous with respect to surface-antigen expression, and that subsets label with W3/13 and OX8 but not W3/25 and OX6 monoclonal antibodies. Despite this heterogeneity, NK and ADCC activity could not be dissociated by W3/13 and OX8 antibodies. Also, there was no evidence that NK-cell heterogeneity was related to target-cell specificity, since in the rat the data demonstrate that those NK cells which kill solid, leukaemic and non-neoplastic cells are identical on the basis of the surface markers examined.

**NATURALLY CYTOTOXIC CELLS IN THE RAT: AUGMENTATION OF NK ACTIVITY BY SPONTANEOUS AND CARCINOGEN-INDUCED TUMOURS.** G. R. FLANNERY & C. G. BROOKS, *Cancer Research Campaign Laboratories, University of Nottingham, Nottingham NG7 2RD*

We have previously shown that NK activity in rats developing spontaneous solid tumours is normal or raised, and our data suggest that impaired NK activity is not associated with spontaneous tumour development. However, early augmentation of NK activity, reported by others, might provide a mechanism for NK surveillance.

Splenic and blood NK activity was assessed in 6h $^{51}$Cr-release tests 3 days after inoculation of $2 \times 10^4$–$5 \times 10^5$ spontaneous or carcinogen-induced tumour cells into the mammary pads. Stimulation of NK activity occurred with 0/5 non-immunogenic tumours but with 3/4 immunogenic tumours. When injected i.p., $10^3$–$10^7$ immunogenic and non-immunogenic tumour cells augmented NK activity of peritoneal exudate cells, though this was seen in 12/31 pairs (39%) of animals injected with immunogenic cells but only 6/45 pairs (13%) of animals given non-immunogenic cells. In addition, 4/4 immunogenic tumours produced NK activation but only 3/6 non-immunogenic tumours did so. Activation was generally lower in animals receiving non-immunogenic tumour cells and high doses of these cells sometimes depressed responses.

These results suggest that early augmentation of NK activity is associated with tumour immunogenicity, and since most spontaneous tumours are non-immunogenic, such augmentation is unlikely to contribute to the potential role of NK cells in immune surveillance.

**INTERFERON PRODUCTION AND NK CELL ACTIVITY OF PERIPHERAL BLOOD LYMPHOCYTES FROM NORMAL CHILDREN AND ADULTS AND PATIENTS WITH ACUTE LYMPHOBLASTIC LEUKAEMIA.** A. M. DICKINSON, S. PROCTOR, E. JACOBS, G. MOHAMMED & G. A. TOMS, *Departments of Haematology and Virology, Royal Victoria Infirmary, Newcastle upon Tyne*

The erythroleukaemia cell line K562 has been shown to stimulate normal lymphocytes into interferon (IFN) production. Also NK cell activity correlated with amount of IFN produced (Peter, *et al.*, 1980, *Eur. J. Immunol.*, **10,** 547). In our studies peripheral blood lymphocytes (PBL) from normal controls and children undergoing treatment for Acute Lymphoblastic Leukaemia (ALL) were tested simultaneously for NK activity and IFN production against K562. NK-activity using $^{51}$Cr release was measured after 4h incubation of the mictotitre plates at 37°C/5% $CO_2$. IFN production required replenishment of the wells with culture medium (RPMI 1640 + 10% FCS) and incubation at 37°C/5% $CO_2$ for 24 h. Supernatants were assayed for IFN by a dye-uptake assay in Vero cells challenged with Semliki Forest virus. 10/13 PBL samples from normal adults produced IFN (range 18–43 u/ml) and simultaneously gave NK activities of 9–62 (mean 33%). Similarly 6/9 samples from normal children (age range 11 months to 11 years) produced IFN (range 16–43 u/ml) and gave a range of NK activities 5–26 (mean 13%). Conversely 12 child ALL patients ($<1$% blasts in PBL) were negative for IFN production and gave low values for NK activity (range 0–17%; mean 4%). Medium alone samples were negative. Previous studies (Reid *et al.*, 1977, *Arch. Dis. Child.*, **52,** 245) showed that ALL patients were immunosuppressed in absolute numbers of T and B cells. Our results have further shown that immunosuppressive treatment also impairs lymphocyte function in these patients.

**NK AND ADCC IN HODGKIN'S DISEASE, NON-HODGKIN'S LYMPHOMA AND RENAL TRANSPLANTATION.** S. J. PROCTOR, A. M. DICKINSON, S. GEORGE & E. JACOBS, *Department of Haematology, Royal Victoria Infirmary, Newcastle upon Tyne*

It has previously been demonstrated that in renal-transplant populations there is a 100-fold increase in non-Hodgkin's lymphoma in the 2-years post-transplant period (Mitchison & Kinlen, 1980, *Prog. Immunol.*, **4,** 645). The present study was designed to assess the effect of Immuran and Prednisone on cell cytotoxicity in an NK and ADCC assay in transplant patients and patients with Hodgkin's disease and non-Hodgkin's lymphoma. Target cell for the NK assay was K562 erythroleukaemia cell line. Chang liver cells were used as targets in the ADCC assay. The cytotoxicity testing was performed on the separated PBL and overnight cultures from affected lymph nodes in a small group of patients with Hodgkin's disease and non-Hodgkin's lymphoma. The result indicate a gross suppression of NK and ADCC activity in transplant patients (mean NK 12%, mean ADCC 8% Cr release in 4h assay) in comparison with normals (mean NK normal 40%, mean ADCC normal 38%). NK and ADCC activity was normal in peripheral blood in Hodgkin's patients and those patients with non-Hodgkin's lymphoma showing complete response to treatment. In a group of 10 high-grade NHL patients (patients dying less than one year from diagnosis) NK and ADCC activity was also significantly reduced (mean NK 22%, mean ADCC 15%). In suspensions from lymph nodes, low activity was seen in 2 patients with Hodgkin's disease demonstrating atypical clinical and cytological features. The possibility exists that the removal of cytotoxic T population or NK populations by immunosuppressive drugs may allow the outgrowth of neoplastic B cells and suggests that the cellular immunosurveillance system has a role in prevention of B-cell tumours.

**FUNCTIONAL CHARACTERISTICS OF NATURAL CYTOTOXICITY MEDIATED BY HUMAN TONSILLAR LYMPHOCYTES.** I. Kimber & M. Moore. *Department of Immunology, Paterson Laboratories, Manchester* 20

The possibility that cytotoxicity mediated by natural killer (NK) cells represents the *in vitro* expression of a non-adaptive mechanism of host resistance to malignancy has generated considerable interest.

Although the capacity of human peripheral and splenic lymphocytes to effect substantial lysis is now well documented, cytotoxicity mediated by cells isolated from other secondary lymphoid tissues is less well characterized, having been found in some studies but not in others.

To further clarify the somewhat elusive, phenomenon of extra-vascular natural cytotoxicity we have examined the functional characteristics of lysis mediated by lymphocytes isolated from human palatine tonsils.

We report that lysis of the NK-susceptible cell line K562 by tonsil lymphocytes, although weak compared with autochthonous vascular lymphocytes, is invariably manifest at high effector:target ratios. Like peripheral NK cells, tonsillar cytotoxic lymphocytes have a low buoyant density which allows their partial enrichment from non-cytotoxic cells by centrifugation on discontinuous Percoll gradients. However, examination of cytotoxic fractions indicates that, unlike vascular cytotoxicity, effector function is not associated with the presence of lymphocytes of characteristic large granular morphology.

Furthermore, a functional distinction from classical NK cells is apparent since, although tonsillar cytotoxicity is significantly enhanced following exposure to supernatants from polyclonally-activated allogeneic tonsils, pretreatment, with IFN-$\alpha$, at doses shown to maximally potentiate peripheral cytotoxicity, fails to influence reactivity.

These data provide preliminary evidence for the existence of, at least limited, heterogeneity among human natural killer cells.

**GENERATION OF SUPPRESSOR CELLS FOR NK CELL ACTIVITY IN CANCER PATIENTS BY SURGERY.** A. Uchida, M. Colot, & M. Micksche, *Institute for Cancer Research, University of Vienna, Austria*

As surgery often increases incidence of metastases, it is important to know the effects of surgery on NK activity and its regulatory mechanism in cancer patients. NK activity against K562 cells determined in a 4h $^{51}Cr$ release assay of untreated patients was comparable to that of normal controls. After surgery blood NK activity significantly fell, while the number of large granular lymphocytes (LGL) did not. When postoperative lymphocytes were depleted of monocytes and cultured for 24 h, they showed an increase in NK activity. However, the mere addition of blood monocytes to cytotoxicity assays did not inhibit NK activity. 24 h preculture of NK cells and postoperative monocytes suppressed NK activity. The monocytes also suppressed the enhancement of NK activity by interferon. These results suggest that postoperative monocytes inhibit the maintenance and development, but not effector phase, of NK cells. After contact with suppressor monocytes, NK cells changed their form from an irregular motile one to a round resting one and lost their active motility, which could be one mechanism of NK suppression, since the motility of NK cells is an important step of NK-mediated cytolysis. This was confirmed by using a new agarose microdroplet assay in which NK cells migrated from an agarose droplet toward target cells in medium and killed them. OK-432, a streptococcal preparation, reduced the suppressor activity of monocytes both *in vivo* and *in vitro*. This suggest that blood monocytes play an important role in the regulation of NK activity in cancer patients after surgery, and its modulation by OK-432 may produce a subsequent benefit to the host.

**AUGMENTATION OF AUTOLOGOUS LYMPHOCYTOTOXICITY (ALC) BY OK-432 – A STREPTOCOCCAL PREPARATION.** M. Micksche & A. Uchida, *Institute for Cancer Research, University of Vienna, Austria*

OK-432, a streptococcal preparation, has been already shown by us to enhance NK activity after *in vivo* and *in vitro* application.

In an ongoing clinical trial, patients with malignant pleural effusions (breast and lung cancer) are treated by intrapleural application of OK-432. Pleural-effusion fluid is collected immediately before injection and lymphocytes and tumour cells are separated by discontinuous gradient centrifugation (Uchida, 1981, *Cancer Immunol. Immunother.*, **11**, 131). Lymphocytotoxicity is determined in a 4h $^{51}$Cr-release assay, using autologous effusion derived tumour cells as targets. Patients with no ALC before therapy were found to express high cytotoxicity soon after the first week of treatment.

*In vitro* studies have demonstrated that 24h pretreatment of lymphocytes with OK-432 led to an increase or induction of ALC, whereas interferon (Hu-IFN $\alpha$) failed to modify this reactivity.

Induction of ALC by OK-432 therapy might be one mechanism responsible for therapeutic response (*i.e.* disappearance of pleural effusion) after intrapleural application of OK-432.

**INDUCED CYTOTOXIC ACTIVITY IN HODGKIN'S AND NON-HODGKIN'S LYMPHOMA.** D. B. Jones, K. Higginson & D. H. Wright, *University Department of Pathology, General Hospital, Southampton SO9 4QY*

The data presented represent a preliminary study of NK activity inducible in mononuclear cells prepared from tissue biopsies of Hodgkin's disease (HD) and non-Hodgkin's lymphoma (NHL).

Mononculear cell preparations from 15 HD spleens showed a significant increase in native NK activity over cells from 20 control spleens. NK activity was low or absent in 7 spleen-cell preparations from patients with NHL. This low activity could not be explained by dilution of effector cells by tumour, or by the absence of T cells.

The induction of cytotoxicity by co-culture with mitomycin C-treated lymphoblastoid cells and polyclonal-lymphocyte activators which induce NK like effectors *via* the induction of interferon synthesis (Potter & Moore, 1981, *Clin. Exp. Immunol.*, **44,** 332) gave comparable results. Cells from HD spleen were easily inducible to high levels of activity against K562 target cells. NHL tissue cells gave levels of induced cytotoxicity which fell below those observed with normal spleen cells. The induction results suggest that qualitative or quantitative differences exist between the spleen-cell populations in HD and NHL, which account for the differing response to polyclonal T-cell activation.

Individual HD spleens also produce significant killing over 5 days in control culture with non-mitogenic human AB serum. This spontaneous activation in culture is related to the level of T cell activation in normal uninvolved HD spleen, and may reflect the production of soluble potentiators of NK activity.

**LYMPHOCYTE SUBSETS INFILTRATING HUMAN MAMMARY CARCINOMAS.** O. Fremin, J. Ashley, M. Brown & S. Williamson, *Department of Clinical Surgery, University of Edinburgh*

Lymphocytes infiltrating human mammary carcinomas have been well documented histologically, but their precise role *in vivo* and possible anti-tumour activity has not been defined. Lymphocytes were isolated from primary human mammary tumours using mechanical and enzymatic preparative techniques and cell passage down a Sephadex G-10 column. Rosetting techniques were used to characterize both T and B lymphocyte subsets within the tumours. The pattern detected was different, in certain respects, from that found in the blood, and suggested a selective subset migration into the tumour. This was confirmed by *in vitro* cytotoxicity assays revealing an absence of K cells and a paucity of NK cells. The study also established the hyporeactive state of mitogen assays of the tumour-infiltrating lymphocytes, in contrast to the normal activity of blood lymphocytes.

**QUANTITATION OF HUMAN TUMOUR-REACTIVE LYMPHOCYTES IN BLOOD AND TUMOUR BY LIMITING-FREQUENCY ANALYSIS.** B. M. Vose, *Department of Immunology, Paterson Laboratories, Christie Hospital and Holt Radium Institute, Manchester M20 9BX*

Antigen-activated T cells can be maintained in conditioned media containing interleukin-2 (IL-2). Limiting-dilution techniques dependent upon IL-2 allow the quantitation of tumour-reactive lymphocytes at different sites by enumeration of cells stimulated in mixed lymphocyte-tumour cultures (MLTC).

Blood lymphocytes (PBL) and those isolated from 8 enzymatically dispersed lung and breast tumours (TIL) were plated under limiting-dilution conditions with irradiated autologous tumour, blood mononuclear cells as feeders and IL-2, and incubated for 7–9 days. Microcultures were assayed for proliferation by uptake of [$^3$H]dT and cytotoxicity against autologous and allogeneic tumour and K562. Tumour-associated lymphocytes showed significantly higher frequencies of (1) spontaneously IL-2-reactive cells (1/900 and 1/4750) and (2) proliferative (1/200 and 1/1200) and cytotoxic tumour-reactive precursors, than blood lymphocytes, but lower frequencies of NK (1/2500 and 1/1600) precursors. 4–8-fold concentrations of tumour-reactive cells were regularly found in TIL. Phenotypic analysis revealed that although proportions of T cells in PBL and TIL were similar (60–85%) TIL showed a marked increase in the number of OKT8$^+$ (cytotoxic/suppressor) cells (50% of total T) compared with the periphery (15–27%). There was a corresponding fall in OKT4 (helper) cells, though early data suggest that TIL were more efficient producers of IL-2 in MLTC than PBL. Taken together these data support the conclusion that there is significant homing of reactive T cells to the tumour site in human neoplasia.

**HUMAN TUMOUR-REACTIVE CULTURED T-CELL LINES: ANTIGENS ON LUNG AND BREAST TUMOURS DETECTED IN THE PRIMED LYMPHOCYTE TEST.** B. M. Vose & G. D. Bonnard, *Christie Hospital, Manchester M20 9BX and National Cancer Institute, Bethesda, MD 20205, U.S.A.*

Co-cultivation of cancer-patients' lymphocytes and autologous tumour cells (MLTC) leads to (1) production of interleukin-2 (IL-2), (2) induction of blasts and their proliferation and (3) the generation of specific cytotoxic effectors. Blasts generated in MLTC from 18 cases were isolated by flotation on discontinuous Percoll gradients and cultured in lectin-free conditioned media from mitogen-stimulated lymphocytes. In all cases growth was rapid so that redilution to $3 \times 10^5$ cells/ml and addition of fresh conditioned media was necessary every 3–4 days. Cultured T-cell lines (CTC) were used as responders in primed lymphocyte tests after 10 days *in vitro*. Decreasing numbers of CTC ($10^4$–$1{\cdot}25 \times 10^3$/well) were dispensed into microtest plates and restimulated with $5 \times 10^4$ irradiated cells or conditioned media in 0·2 ml microcultures. Proliferation was assessed by uptake of [$^3$H]dT over the last 6 h of a 48h test. Restimulation of MLTC-blast CTC was obtained in all cases with autologous tumour cells, but not with autologous monocytes, tumour-associated macrophages or lymphocytes. Tests are in progress with normal lung cells. Similar levels of restimulation were induced by allogeneic tumours of the same site and histology as the autologous. Tumours from different sites or histologies, allogeneic monocytes and lymphocytes, did not induce a significant increase of [$^3$H]-dT uptake. This suggests that human tumours of common site and histology express cross-reactive specificities, the nature of which has yet to be identified. Cloned T

cells from MLTC blasts are presently under investigation to facilitate further analysis.

### CYTOXICITY OF HUMAN BRONCHO-ALVEOLAR MACROPHAGES AND PERIPHERAL-BLOOD MONOCYTES FOR CULTURED HUMAN LUNG TUMOUR CELLS.

S. Swinburne & P. Cole, *Host Defence Unit, Department of Medicine, Cardiothoracic Institute, Brompton Hospital, Fulham Road, London SW3 6HP*

As part of the potential host anti-tumour response, mononuclear phagocytes may enter a bronchial tumour from at least 2 sources, blood and bronchus. Human bronchoalveolar macrophages (BAM) were purified from bronchial lavage fluid obtained from patients undergoing diagnostic fibreoptic bronchoscopy. Human peripheral blood monocytes (PBM) were purified from the blood of normal volunteers. The cytotoxic activities of the BAM and PBM for a cultured human lung tumour cell line, A549, were then tested using a modification of the $^{75}$Se-methionine uptake assay devised by Brooks *et al.* (1978) *J. Immunol. Meth.*, **21**, 111.

The cytotoxicity of BAM increased in a dose-dependent manner, approaching 100% at an effector cell target cell ratio of 20:1. The dose–response nature of the cytotoxicity was similar, whether the BAM were obtained from the lungs of patients with mild bronchial inflammation, chronic bronchitis, cryptogenic fibrosing alveolitis, allergic alveolitis or bronchial carcinoma. BAM were cytostatic at lower E:T ratios but cytolytic at higher ratios. In contrast, PBM cytotoxicity plateaued at an E:T ratio of 3:1 and was only cytostatic.

The results of kinetic studies of BAM and PBM cytotoxicity and the ability of BAM, but not PBM, to be cytotoxic for 2 additional human lung cell lines, E14 and MS853, suggest that there are qualitative differences in cytotoxicity between PBM and BAM.

### MONOCYTE–LYMPHOCYTE INTERACTION IN HODGKIN'S DISEASE.

I. H. Manifold, M. D. Whitham, L. Bruce & B. W. Hancock, *University Department of Medicine, Royal Hallamshire Hospital, Sheffield*

In Hodgkin's disease, it is thought that lymphocyte transformation (LT) is inhibited *in vitro* by prostaglandin-secreting monocyte suppressor cells. Previous studies have depleted monocytes from monocyte-lymphocyte cell suspensions. by methods said to be traumatic and prolonged. (Alonso *et al.*, 1978, *J. Immunol. Meth.*, **22**, 361). We have selectively depleted monocytes using the more recent method of one 15min passage through Sephadex G10 columns in 11 untreated Hodgkin's patients and 11 age/sex-matched controls. This produced no significant change in mean percentage T cells, a small decrease in B cells (12·2–10·5% in patients; 10·2–9·7% in controls), but greatly reduced monocytes (18·6 to 1·3% in patients; 11·8% to 1·3% in controls. LT was measured before and after monocyte depletion. LT was grossly reduced in patients from controls but monocyte depletion caused a marked LT enhancement to sub-optimal concentration of PHA in patients in autologous serum (3·79 mean ct/min $\times 10^{-3}$ *vs* 7·67, $P<0{\cdot}001$) indicating the dominant effect of monocyte suppressors. Enhancement was masked by indomethacin, present in pre- and post-column passage, and indomethacin added to cultures with undepleted monocytes, mimicked the effect of monocyte depletion, indicating that prostaglandin mediates the suppression. This pattern was not seen in controls nor in AB serum, nor with PWM and Con-A as mitogens. Rather in some of these series monocyte depletion significantly reduced LT, suggesting that monocyte helper cells were predominant. Further in some of these series, a subpopulation of suppressor monocytes appeared to be present with the helper cells, since indomethacin still caused LT enhancement. Thus monocytes have an important but complex role in modulating LT in Hodgkin's disease, and Sephadex column passage is a useful technique in its analysis.

### DISTINCT SUPPRESSOR-CELL POPULATIONS IN SPLEENS OF TUMOUR-BEARING MICE.

S. Howie & W. H. McBride, *Department of Bacteriology, University of Edinburgh EH8 9AG*

Spleens of tumour-bearing mice contain tumour-specific T helper cells which will collaborate with hapten primed B cells to induce specific antibody *in vitro*. Helper activity disappears when the tumour is large. This loss of helper activity coincides with the appearance of 2 distinct types of suppressor cells (a) nylon–wool-non-adherent T cells which are tumour specific and (b) nylon–wool-adherent non-T cells which are not specific in their action.

**INDUCTION OF CELL-MEDIATED CYTOTOXICITY TO A HUMAN LEUKAEMIA: DIFFERENCES BETWEEN THE RESPONSE OF A PATIENT AND HLA-MATCHED SIBS.** G. M. Taylor, *Department of Medical Genetics, St Mary's Hospital, Manchester M13 0JH*

Cell-mediated cytotoxicity (CMC) was induced in mixed lymphocyte cultures to a human acute leukaemia. We have studied the capacity of different HLA-mismatched lymphocytes to stimulate CMC to autologous leukaemia (aut L) cells in lymphocytes from a patient and his HLA-identical sibs. The results showed differences in the response to different allogeneic cells, and between the patient and sib's response to the aut L. The patient generated CMC to aut L in response to allogeneic stimuli, which was synergistically amplified by aut L, but did not respond to aut L alone. The sib responded to aut L and to allogeneic cells but did not respond synergistically to a mixture of the two. Stimulation of sib lymphocytes with HLA mismatched sib cells gave CMC to aut L irrespective of the HLA-type of the stimulator. Parallel tests on targets sensitive to NK-lysis, suggest that the leukaemia was sensitive to NK-CMC. No evidence was found in CMC assays on sib targets that leukaemia cells expressed inappropriate HLA antigens, though a number of non-HLA-directed reactions were found.

**ENHANCEMENT OF TRANSPLANTATION IMMUNITY TO A SPONTANEOUS MURINE TUMOUR.** A. Vyakarnam, P. J. Lachmann & K. Sikora, *Ludwig Institute for Cancer Research, Mechanisms in Tumour Immunity Unit, MRC Centre, Hills Road, Cambridge*

Previous work from our laboratories has shown that immunization with tumour cells coupled to tuberculin (PPD) can enhance the *in vivo* transplantation immunity to some chemically induced murine tumours in the syngeneic host. PPD is strongly recognized by T cells, and it was suggested that the coupling of PPD on to tumour cells may provide T-cell help in the induction of the anti-tumour response. Spontaneously arising murine carcinomas which may well provide more realistic models for human cancer are claimed to be non-immunogenic in conventional rejection assays. We have examined the antigenicity of a spontaneous murine carcinoma using PPD as an immunogenic carrier determinant. It was observed that multiple immunizations of PPD-coupled tumour cells did potentiate a significant anti-tumour response. Moreover such immunizations retarded the growth of tumours in previously un-immunized animals. Such enhancement may well have clinical application.

**HETEROGENEITY OF CIRCULATING IMMUNE COMPLEXES IN LUNG CANCER AND OTHER CHRONIC PULMONARY DISEASES.** K. M. Cooper & M. Moore, *Paterson Labs., Christie Hospital and Holt Radium Inst., Manchester*

Circulating immune complex (IC) levels in sera from 58 healthy controls and a total of 212 patients with various chronic lung diseases, including 74 with bronchial carcinoma were measured using 3 assays: 2 complement-dependent assays (Clq fluid phase and Raji) and a complement-independent assay (L1210). The 3 assays generally revealed similar patterns of reactivity when control and pathological groups were compared by the L1210 assay invariably demonstrated the lowest incidence of positive values in each group. The most significant elevations in IC levels were in bronchiectasis (chronic bronchial suppuration) and bronchial carcinoma. However, within these 2 groups, correlations between IC assays performed on individual sera were poor. Significant but weak correlations ($P < 0{\cdot}01$) were only seen for Clq *vs* Raji in bronchiectasis and Clq *vs* L1210 in bronchial carcinoma. Complement-binding ICs were present to a similar extent in both conditions. However, non-complement binding ICs were found to be commoner in bronchiectasis. Although interfering factors may contribute to the disparity between the assays, the interpretation favoured is that the lack of correlation is primarily a reflection of the intrinsic heterogeneity of immune complexes formed under similar and dissimilar pathological conditions.

### A COMPARISON OF METHODS FOR IMMUNIZING PIG MESENTERIC LYMPH NODES AGAINST HUMAN TISSUE.

D. M. Morris, D. Heinemann & M. O. Symes, *Departments of Obstetrics & Gynaecology and Surgery, University of Bristol, Bristol*

The use of immune pig mesenteric lymph-node cells to treat tumours in mice (Pritchard-Thomas & Symes, 1978*a*, *b*, *Cancer Immunol Immunother*, **4,** 129, 135) and man (Symes *et al.*, 1978, *Urology*, **12,** 398) has been reported. In order to study the optimal route and time of immunization, fragments of human skin were implanted into pockets in the mesentery or directly on to the surface of the mesenteric node. Alternatively, human leucocytes were injected into Peyers Patches. Three, 5, 7 or 14 days after immunization the reactivity of the lymph-node cells were compared, using a $^{75}$Se-release assay against human PBL as targets. Nodes immunized for 7 or 14 days showed no cytotoxicity. However, after 3 or 5 days immunization co-culture of effector and target cells produced negative cytotoxicity, in that $^{75}$Se release was less than that from target cells cultured alone (% cytotoxic $-14$ to $+20\%$ at an E/T ratio of 40:1). This phenomenon was more marked using LNC cells, from nodes in contact with human skin. It suggested there was recognition of the target by the effector LNC, which did not proceed to target cell lysis. Recognition was confirmed by increased uptake of [$^3$H]dT by LNC, showing negative cytotoxicity. Mesenteric lymph nodes immunized for 3 days showed hyperplasia of the thymus-dependent cortex, not seen after 7 days immunization.

### PIG LYMPH-NODE-CELLS IN THE TREATMENT OF THE OVARY.

G. M. Turner, D. Morris, V. Barley, E. Rhys Davis & M. O. Symes, *Departments of Obstetrics and Gynaecology, Radiotherapy, Radiodiagnosis and Surgery, University of Bristol*

Pig mesenteric lymph-node cells have been used to treat patients with $T_3$ or $T_4$ carcinoma of the ovary who have previously received standard treatment (i.e. surgery, $\pm$ chemotherapy $\pm$ radiotherapy).

The Phase 1 study (Turner & Symes, 1979, *Br. J. Cancer*, **40,** 823) now involves 6 patients in whom patient tumour or skin immune pig cells were used. Two patients are alive 7½ and 1¾ years later. One patient who died 2 months after treatment showed complete disappearance of previously existing multiple i.p. tumours. Two patients had objective evidence of tumour remission (in one there was no recurrence of previously persistent ascites and in the other a return to normal activity and weight). However, both patients eventually died from progressive disease. One further patient died within 2 weeks of treatment and showed haemorrhagic tumour necrosis at autopsy.

In the Phase 2 study similar patients were allocated at random to receive non-immune or immune pig cells. To date there are respectively 6 and 9 patients in the 2 arms of this study. All the patients have died and the survival times in days from treatment have been for non-immune cells, 9, 13, 34, 73, 91 and 116; and for immune cells 14, 25, 36, 47, 59, 62, 84, 98 and 150. One patient treated with non-immune cells has shown evidence of partial disease remission. Of the patients receiving immune cells one had a partial remission.

### A RANDOMIZED TRIAL COMPARING VINDESINE VDS WITH VINDESINE PLUS *CIS*-PLATINUM (DDP) IN INOPERABLE NON-SMALL CELL LUNG CANCER (NSCLC).

J. A. Elliott*, S. Ahmedzai†, R. D. Stevenson†, A. J. Dorward & K. C. Calman‡, **Western Infirmary; †Royal Infirmary, ‡Department of Clinical Oncology University of Glasgow*

Between January 1981 and January 1982, 59 patients with previously untreated inoperable NSCLC were randomly allocated to treatment with VDS (n=29) or VDS+DDP(n=30). As a single agent VDS was given in a dose of 3–4 mg/m$^2$ weekly $\times$ 8 and 4 mg/m$^2$ 2 wk thereafter in responding patients. In combination VDS was given in a dose of 3·0 mg/m$^2$ weekly $\times$ 8 and 3 mg/m$^2$q 2 wk thereafter in responders) together with 100 mg/m$^2$ DDP with mannitol-induced diuresis at 0, 4 and 8 wks, then q 6 wks thereafter in responders.

40 patients are fully evaluable. There was one partial tumour response in 23 patients given VDS alone (4%). Of 17 evaluable patients receiving VDS+DDP 9 (53%) showed a partial response.

Myelosuppression was mild in both groups, but significantly greater with the combination. Significant neurotoxicity, principally paraesthesiae and sensory impairment, was manifested by most of patients; severe neuropathy was seen with equal frequency in both groups (7%). Urinary creatinine clearance fell in nearly all patients receiving DDP, which prevented further DDP in 2 patients.

Our preliminary results confirm that VDS+DDP is an active combination in NSCLC and suggest its superiority over VDS as a single agent. The study is on-going. Survival data are not yet available.

### INTERMITTENT HIGH-DOSE CYCLOPHOSPHAMIDE WITH AND WITHOUT PREDNISOLONE IN METASTATIC LUNG CANCER.

N. Thatcher, J. Wagstaff, H. Anderson, M. Palmer & D. Crowther, *Depts. Medical Onolocy and Medical Statistics, Christie Hospital, Wilmslow Road, Manchester M20 9BX*

57 patients with metastatic lung carcinoma were treated with either high-dose cyclophosphamide (CY) alone or with a combination of high-dose CY and Prednisolone (100 mg/m$^2$ o.daily × 2). The CY was given i.v. on 3 occasions at 1·5 g/m$^2$, 2·5 g/m$^2$ and 3·5 g/m$^2$ with 3 week intervals between courses.

The overall response rate was 57% (18% CR) median survival 24 weeks (range 6–130) for CY alone and 24% (3% CR) median 14 weeks (1–94) for Cy+Pred. Patients with small-cell carcinoma given CY alone had a 69% response rate (19% CR) median survival 7 months and with non-small-cell-pathology 42% (16% CR) median survival 16 weeks. Performance scores and survival were better for responding patients. Addition of Prednisolone did not improve the therapeutic efficacy of high-dose CY, nor ameliorate toxicity. No marked or unexpected toxicity was observed with the high-dose CY. Blood counts had returned to normal by 3 weeks in the great majority of patients.

A short course of high-dose CY was not associated with unacceptable side-effects, and the therapeutic results obtained were superior to those described by CY at conventional dosage. High-dose CY is of value to patients with metastatic lung cancer, and the incorporation of the regimen into chemotherapeutic combinations could be advantageous.

### FACTORS INFLUENCING RESPONSE TO CHEMOTHERAPY IN SMALL-CELL ANAPLASTIC LUNG CANCER.

A. Gregor, J. Carmichael, G. K. Crompton, I. W. B. Grant & J. F. Smyth, *Departments of Clinical Oncology and Medicine, Western General Hospital, Edinburgh*

73 previously untreated patients with histologically proven small-cell carcinoma of the bronchus were treated with methotrexate (MTX) (200 mg/m$^2$ i.v. over 24 h) and cyclophosphamide (CY) (1 g/m$^2$ i.v. q 3/52) with CCNU (100 mg/m$^2$ p.o. q 6/52). Mean age was 59·4 years (range 28–75). 33% of patients were females. With conventional staging, 47% had extensive disease. 69% of patients had performance status (PS)M1. Assessment of response, including repeat bronchoscopy, was carried out at 12/52. Objective response rate was 57%, with 23% complete response (CR). Of 24 females, 70% responded and 30% achieved CR, despite 70% having extensive disease. In comparison, only 50% of males responded (20% CR rate), 35% having extensive disease. 65% of patients with PS, none responded, with 34% PS 0+1 patients achieving CR. 20% of extensive-disease patients achieved CR. Only 1/11 patients with liver metasases, and none of the 7 patients with marrow involvement, achieved CR. 3/5 patients with inappropriate ADH secretion responded (1 CR) but both patients with inappropriate ACTH secretion were non-responders. Sex and performance status were the principal factors influencing response to chemotherapy.

### PILOT STUDY OF COMBINATION CHEMOTHERAPY WITH LATE DOSE INTENSIFICATION AND AUTOLOGOUS MARROW RESCUE IN SMALL CELL BRONCHIAL CARCINOMA.

S. Banham, A. Burnett, R. Stevenson, D. Cunningham, S. Kaye, S. Ahmedzai, M. Soukop, *Glasgow Royal Infirmary and Gartnavel General Hospital, Departments of Respiratory Medicine, Haematology and Oncology*

The long-term survival in small-cell bronchogenic carcinoma remains poor despite good initial responses to combination chemotherapy. A prospective pilot study of the role of late dose intensification, in selected patients, with Cyclophosphamide 12g i.v. and autologous-marrow rescue, following 5 courses of combination chemotherapy with Cyclophosphamide (1 g/m$^2$), Adriamycin (40 mg/m$^2$), Vincristine (2 mg) and Prednisolone (CHOP) was commenced in Glasgow in January 1981. 44 patients have been admitted to the study, 40 being evaluable at present. 14 patients have received high-dose Cyclophosphamide therapy (HDCT) including both patients with limited and extensive disease and with responses to induction chemotherapy varying from a complete response to disease progression on treatment. All patients, receiving HDCT who had residual tumour following induction chemotherapy, subsequently had a further reduction in tumour size on HDCT. Several patients with limited disease and a poor response to induction regime of CHOP subsequently had good responses to HDCT, with only minimal residual tumour being detected. HDCT was well tolerated even by unwell patients with major weight loss and progressive disease. One patient died on Day 10 post HDCT and post-mortem suggested an acute cardiomyopathy. Aspirated marrow was stored at 4°C and reinfused at about 36 h from the commencement of HDCT. Subsequent marrow recovery occurred in all patients between 9 and 17 days post therapy. We consider HDCT a useful addition to the treatment of small-cell bronchial carcinoma, but its proper integration in the treatment requires further investigation.

**THE RESPONSE OF HUMAN LUNG-TUMOUR XENOGRAFTS TO HIGH-DOSE MELPHALAN.** E. WIST* & J. L. MILLAR†, **The Norwegian Radium Hospital, Montebello, Oslo, Norway and †Institute of Cancer Research, Sutton, Surrey*

The response of human tumour xenografts to cytotoxic treatment has correlated well with the response in the patients from whom the tumours were derived (Shorthouse *et al.*, 1980, *Br. J. Surg.*, **6**, 715). It was hoped that by studying the melphalan uptake in different lung tumour xenografts, a relationship could be established with their response to treatment with melphalan. No such relationship was found.

Indeed, the more chemosensitive oat-cell xenografts took up less drug than the more chemoresistant large-cell and adenocarcinoma xenografts. The response of these oat-cell tumours, as measured by *in situ* growth delay, was, as expected, consistently greater than that of the more chemoresistant tumours.

During these investigations, the residual red-cell volume, plasma volume, extracellular volume and vascular permeability of these xenografts was measured at different stages of their growth. One striking finding of these studies was how rapidly the uptake of melphalan, per g tissue, declined with increasing tumour size. Tumours were measured from 0·1–2 g and over this weight range uptake of melphalan per g of tissue declined a decade. The implications of this in the adjuvant use of chemotherapy will be discussed.

**PROGNOSTIC VALUE OF CYCLIC AMP-BINDING PROTEIN IN HUMAN BREAST CANCER.** R. O. SENBANJO, W. R. MILLER, J. TELFORD, D. M. A. WATSON & A. P. M. FORREST, *Department of Clinical Surgery, University of Edinburgh*

Cyclic AMP and its binding proteins are involved in regulating the growth of mammary tumours in experimental animals (Cho-chung, 1978, *Cancer Res.*, **38,** 4071; Cho-chung *et al.*, 1978, *J. Biochem.*, **86,** 51. In the present study, we have investigated the relationship between cyclic AMP-binding proteins in human breast cancers and clinical parameters including prognosis. The binding proteins were measured in the cytosols from 75 human breast cancers. All tumours contained measurable amounts of binding proteins, levels varying from 0·81 to 15·05 pmol/mg cytosol protein (mean = 5·34). No relationship was found between level of activity and menopausal status of the patient, clinical stage of the disease and presence or absence of nodal involvement. Poorly differentiated tumours have significantly higher binding activity than better differentiated tumours ($P < 0{\cdot}01$).

A group of 10 patients who at primary treatment had no evidence of metastatic disease, but have developed recurrence within 36 months have been compared with a similar group of 11 with a minimum of 24 months follow-up and were disease-free. Levels of cyclic AMP-binding proteins were significantly higher in tumours from women having early recurrence than in those disease-free ($P < 0{\cdot}005$ by Wilcoxon rank test). The range in disease-free patients was 1·57–7·21 pmol/mg cytosol protein; whereas in 9 of 10 tumours that subsequently recurred early it was 7·75–13·02. It is concluded that although levels of cyclic AMP binding protein are not associated with the clinical parameters described, it may be of independent prognostic significance.

### A COMPUTER PROGRAMME TO ASSIST THE PREOPERATIVE DIAGNOSIS OF AXILLARY-LYMPH-NODE METASTASES IN BREAST CANCER.

R. J. C. Steele, J. D. McGregor, O. Eremin & A. P. M. Forrest, *Departments of Clinical Surgery and Pathology, University of Edinburgh*

Clinical examination is known to be inaccurate in the diagnosis of axillary lymph-node metastases in breast cancer (Wallace & Champion, 1972, *Lancet*, i, 217). An attempt has therefore been made to improve this by combining several factors known to be associated with nodal tumour spread.

In 100 patients with breast cancer, nodal metastases were found to be significantly associated with tumour size, tumour border, histological grade and duration of symptoms. These parameters, along with clinical findings, were then used to create a data base from which the probability of nodal involvement by tumour could be derived. The calculation was carried out by a basic language computer programme, which used Bayesian probability theory. This programme was then tested on a total of 159 patients and achieved an overall accuracy rate of 84%, compared with 65% from clinical examination alone.

Such computer-assisted diagnosis may be useful in the preoperative assessment of breast-cancer patients, and may have implications for the selection of therapy.

### REACTIVITY OF MONOCLONAL ANTIBODIES AGAINST HUMAN LUNG CANCER CELL LINES.

D. T. Brown & M. Moore, *Paterson Laboratories, Christie Hospital and Holt Radium Institute, Manchester* 20

A panel of monoclonal antibodies was produced by fusion of sensitized murine spleen cells with the NS-1 myeloma, against several human lung carcinoma cells, including the E14 and BEN lines (squamous-cell carcinoma). Most of the monoclonal probes, tested by radioimmunoassay, recognized species-specific antigens or lung differentiation antigens. However several of the antibodies showed reactivity against target antigens with a more restricted distribution. Antibodies designated 7B3·5, 7B5·4 and 7B17·7 were reactive with a variety of human carcinoma cell lines derived from several different tissues, but were negative against several normal lung lines. Two monoclonal antibodies (7B24·4, 7BC9·1) recognized a target antigen found only on the immunizing cell line (BEN) and in the case of 7BC9·1 at a much lower density on one colorectal carcinoma cell line (W1DR). These antibodies should prove useful for the definitive characterization of putative human tumour-associated antigens in lung and other cancers.

### MONOCLONAL ANTIBODY ISOTOPE SCANNING IN THE DETECTION OF METASTATIC CARCINOMA.

H. M. Smedley,, K. Sikora, E. Lennox & P. Wraight, *Ludwig Institute for Cancer Research, MRC Centre, MRC Laboratory of Molecular Biology, Department of Nuclear Medicine, Addenbrooke's Hospital, Hills Road, Cambridge*

We present our initial results together with representative scans obtained from patients with metastatic cancer following the injection of radioisotope-labelled monoclonal antibodies. Rat monoclonal antibodies were prepared by immunizing rats with human colorectal carcinoma cell membranes and fusing splenic lymphocytes with a rat myeloma, Y3 Ag 123. Hybridoma supernatants were screened by binding assays on colorectal carcinoma cell lines. One hybridoma supernatant contained a monoclonal antibody with high binding activity and was grown in large quantities in serum-free medium. After ammonium and sulphate precipitation the antibody was purified by ion-exchange chromatography. A modified version of the chloramine-T method was used for iodination with $^{131}I$. Binding assays to cell lines and *in vivo* experiments on xenograft-bearing immune-deprived mice revealed that this coupling method leaves the biological activity and specificity of the monoclonal antibody substantially unaltered. This preparation has been injected into patients with metastatic, colorectal and breast tumours without untoward side-effects. Gamma-camera scans have been obtained at 6, 24 and 48 h and following estimates of the blood pool using conventional imaging reagents, computerized subtraction scans were obtained showing areas of localized high uptake corresponding well with areas of known disease. No clinical complications of this technique have been encountered, and one patient has been scanned on 2 separate occasions without apparent problems. This work is currently being expanded to use other monoclonal antibodies with patients with different types of malignancy.

### THE DETECTION OF BLOOD-GROUP ISOANTIGEN ON SUPERFICIAL BLADDER TUMOURS USING MONOCLONAL ANTIBODIES AND ITS VALUE IN PREDICTING SUBSEQUENT INVASIVE RECURRENCE.

P. J. Finan*, E. S. Lennox†, & N. M. Bleehen*, **MRC Clinical Oncology Unit and* †*MCR Laboratory of Molecular Biology, Cambridge*

It has been claimed that loss of normally occurring blood-group isoantigen (BGI) on malignant transitional epithelium is a reliable predictor of subsequent invasive recurrence (Limas & Lange, 1979, *Cancer*, **44**, 2099). Previous workers have used the specific red-cell-adherence test together with conventional antisera. However, problems have been encountered both with the technique and reproducibility of results. We have therefore investigated a more direct method for demonstrating the presence of BGI on normal and malignant transitional epithelium.

Using an indirect immunoperoxidase technique and monoclonal antibodies to the blood groups A and B (Voak *et al.*, 1980 *Vox. Sang.*, **39**, 134; Sacks & Lennox, 1981, *Vox. Sang.*, **40**, 99) BGI was detected on paraffin sections of normal urothelium from 14 patients. On initial biopsies from 39 patients with superficial bladder tumours, followed for 5 years or until muscle invasion occurred, BGI was detected in 31 (79%). The rate of invasive recurrence in the isoantigen-group was significantly higher than in the isoantigen$^-$ group ($P=0{\cdot}04$, Fisher's exact test).

Using these specific reagents, blood-group isoantigen may be readily detected on paraffin-embedded material. Furthermore loss of isoantigen expression would appear to identify a group of patients at higher risk of developing an invasive recurrence, who should be subjected to closer follow-up.

### CIRCULATING LYMPHOCYTES IN BLADDER-CANCER PATIENTS TREATED WITH MEGAVOLTAGE X-RAY THERAPY.

L. L. Alexander*, W. Duncan†, C. M. Steel*, & J. N. Webb‡, **MRC Clinical and Population Cytogenetics Unit,* †*University of Edinburgh and Radiation Oncology Unit, Western General Hospital and* ‡*Dept of Pathology, W.G.H., Edinburgh*

30 patients with transitional-cell carcinoma of bladder have been studied by serial counts of circulating lymphocytes, and of T cells before, during and at intervals (up to 3 years) after radiotherapy.

Total lymphocyte counts fell, reaching a nadir (less than 50% of pretreatment levels) one month after the end of treatment. Recovery of lymphocyte numbers was very gradual and did not approach pretreatment levels for about 2 years. The proportion of T cells remained within normal limits throughout. Nine patients died of their disease within 18 months of entering the study. In this group, pretreatment lymphocyte counts were higher and post-treatment counts lower than in the remaining patients. This correlation between patterns of change in circulating lymphocytes and survival was independent of either stage grade of the original tumours.

Lymphocytes from every blood sample have been stored in liquid $N_2$. With the availability of monoclonal antibodies, these are now being examined to establish the behaviour of T-cell subsets in this group of patients.

### A POPULATION-BASED STUDY OF FAMILIAL ASPECTS IN SOFT-TISSUE SARCOMAS OF CHILDHOOD.

J. M. BIRCH & A. L. HARTLEY, *University of Manchester, Department of Epidemiology and Social Research, Christie Hospital and Holt Radium Institute, Manchester M20 9BX*

A familial syndrome involving soft-tissue sarcomas (STS) in children and early-onset breast cancer in their mothers has been described. At present neither the proportion of familial childhood STS nor the risk to the mothers can be estimated. The present study, based on the Manchester Children's Tumour Registry (MCTR), seeks to do this, and to identify features which distinguish familial from non-familial cases.

Between 1954 and 1981, 154 cases of STS were included in the MCTR. The ratio of boys to girls was 1·4:1. The median age at onset was 4 years among the largest group, the rhabdomyosarcomas.

A search to the MCTR records of these 154 cases revealed: (a) 5 sib-pairs involving a STS compared with 5 sib-pairs with other tumours, among ~2800 cases in the MCTR as a whole; (b) 2 mothers with early-onset of breast cancer, including the mother of one of the sib-pairs; and (c) 14 other cases with close relatives who had early onset breast, childhood or other cancers. In addition 9 of the 154 cases had congenital abnormalities and 5 had 1 or more first degree relatives with congenital abnormalities. There were 4 cases who were one of a pair of twins and 5 cases who had twins amongst their close relatives. This represents an increased frequency of both congenital malformations and twinning.

More detailed investigations of children with STS and their families are under way to characterize the familial cases further. The work has important implications for genetic counselling.

### MARROW INVOLVEMENT IN ADULT SOFT TISSUE SARCOMAS.

V. BRAMWELL, M. B. LITTLEY, J. CHANG, & D. CROWTHER, *Depts of Medical Oncology, Haematology, Christie Hospital, Manchester and Stepping Hill Hospital, Stockport*

As marrow involvement is common in disseminated childhood rhabdomyosarcoma, and implies a poor prognosis, we wished to assess the incidence and significance of marrow infiltration in adult soft tissue sarcoma. There was invasion of the marrow by tumour in 4/74 cases, all from the group of 56 patients who had other evidence of metastatic disease, giving an overall incidence of 7%. Angiosarcomas of the breast are extremely aggressive, and long-term survivors are rare. Our only patient with this diagnosis had marrow invasion demonstrated by trephine biopsy, and rapidly succumbed, despite intensive chemotherapy. About 12% tumour giant cells were found in the marrow aspirate from a patient who had a solitary pulmonary metastasis from a pleomorphic rhabdomyosarcoma of the right biceps muscle. A third case had widespread metastases from a poorly differentiated sarcoma of uncertain histogenesis. Marrow aspirate and trephine demonstrated almost complete replacement of normal marrow by undifferentiated tumour cells. A single case of well differentiated myxoid liposarcoma also metastasized to the marrow. This tumour disseminated widely, yet the metastases retained the low mitotic rate and myxoid appearances of the primary. Although 27% of the total material comprised leiomyosarcomas, none metastasized to marrow. Durations of survival from the time of documented marrow involvement were 4, 5, 20, 19+ months, and did not differ significantly from those of the whole group with metastatic disease (median 11 months). Although it was not possible to determine whether response to chemotherapy was influenced by marrow involvement, haematological toxicity seemed excessive.

### LECTIN BINDING TO THE PERIPHERAL BLOOD MONONUCLEAR CELLS OF PATIENTS WITH LYMPHOMA–CORRELATION WITH CLINICAL STAGE AND OTHER SURFACE MARKERS.

G. BLACKLEDGE, J. GALLAGHER, A. MORRIS & D. CROWTHER, *CRC Dept of Medical Oncology, Christie Hospital, Manchester M20 9BX*

The binding of various lectins to the surface membranes of different cell types has been reported previously and using Con A, LCA and WGA, characteristic binding patterns have been obtained for acute myelogenous, acute lymphoblastic and chronic lymphocytic leukaemia. The peripheral blood mononuclear cells of patients with lymphoma have been studied by flow cytometry using the method previously described (Blackledge *et al.*, 1980, *Flow Cytometry*, **4**, 222). It has been found that there are particular patterns of lectin binding to PBL cells in different pathological types of lymphoma. Those lymphomas with a poorly differentiated cell type (Rappaport) regardless of whether nodularity exists in the tumour, have a repeatable pattern. Tumours with the so-called histiocytic type of cell also have a reproducible pattern of lectin binding, with a population of cells having an increased lectin binding. Cells from patients with DWDL lymphoma resembled those found in CLL. These results suggest that regardless of morphological evidence of blood involvement by lymphoma, there are definite changes in the peripheral blood mononuclear cells, which may indicate abnormal cells in the peripheral blood or some other influence on the binding of lectins to normal PBL.

### TREOSULFAN CHEMOTHERAPY IN ADVANCED OVARIAN CANCER: A LONG-TERM EVALUATION IN PREVIOUSLY UNTREATED DISEASE.

W. F. White & J. E. Masding, *Regional Centre for Radiotherapy and Oncology, Guildford, Surrey*

Previously presented data (White, 1982, *Curr. Chemother. and Immunother.*, in press) indicated that treosulfan chemotherapy administered in an intermittent regimen was superior to a continuous regimen, in terms of improved survival and reduced toxicity. This communication reports on progress of patients with ovarian cancer on intermittent treosulfan, and includes a number of patients enrolled since the previous evaluation.

From November 1977 to December 1980 inclusive, a total of 56 patients with advanced (FIGO Stages III/IV) non-radically operable, ovarian cancer of epithelial origin were treated with treosulfan, in a regimen which usually comprised 1 g daily for 28 days followed by 28 days off treatment. Patients aged 75 or over were started at 750 mg/day. All patients had residual disease after operative procedures, and significant surgical reduction of tumour masses was carried out in only 20 cases.

Of 47 fully evaluable patients, there were 18 complete responders (CR:38%) 14 partial responders (PR:30%). and 15 non-responders (NR:32%). Median survival of CRs is at present >19 months, of PRs 11·5 months and NRs 9 months. Of the CRs, 6 are continuing in remission, 3 are alive with recurrent disease and 9 have died. Two PRs are in complete remission at 12 and 40 months, due to radical "2nd-look" laparotomy. Dose-limiting toxicity was haematological: 35 (74%) of patients required dose modification due to leucopenia and/or thrombocytopenia. The commonest side-effect was a generalized skin pigmentation which occurred in 12 (26%) patients.

Treosulfan is a well-tolerated treatment for advanced ovarian cancer, and produced a higher remission rate than those generally reported for other types of alkylating agent.

### TOTAL ABDOMINAL AND PELVIC IRRADIATION FOLLOWING TREATMENT WITH *CIS*-DICHLORODIAMININE PLATINUM II (CDDP) AND SECOND-LOOK LAPAROTOMY IN PATIENTS WITH ADVANCED OVARIAN CARCINOMA: A PHASE I STUDY.

K. K. Chan, T. A. Latief, G. A. Newsholme, T. J. Priestman, C. E. Newman, R. A. Hurlow, J. Fielding, D. Luesley & A. Howell, *Departments, Medicine and Obstetrics & Gynaecology, Queen Elizabeth Hospital, Birmingham*

It has been shown that total abdominal and pelvic irradiation (TAR) improves survival in patients with microscopic residual disease after surgery for ovarian carcinoma (Dembo, *et al.*, 1979, *Am J. Obstet Gynaecol*, **134,** 793). The aim of this study was to reduce bulky disease with CDDP × 5 courses and cytoreductive surgery, and to treat patients with no macroscopic residual disease with TAR.

Eleven patients were treated according to this protocol between 1979 and 1981. CDDP 100 $mg/m^2$ was given at 3-weekly intervals × 5 followed by second-look laparotomy. Only patients with a surgical complete remission or who could be converted to no macroscopic residual disease were entered. TAR was given by the strip technique (Dembo *et al.*, 1979, *J. Radiol Oncol. Biol. Phys.*, **5,** 1933). Two patients failed to complete TAR, because of disease progression during treatment. Nine completed treatment; toxicity included diarrhoea (3) vomiting (2) nausea (2) abdominal colic (1) and parasthesiae (3). Treatment was delayed because of marrow suppression in 3 patients (1 leukopenia, 2 thrombocytopenia). One patient required transfusion 8 weeks after TAR began. Mean creatinine clearances before CDDP, before surgery and after TAR were 66·7, 77·3 and 72·7 ml/min; no early renal toxicity was seen. Five patients are alive and well at 36, 24, 19, 17 and 8 months, one alive with disease and 3 died with progressive disease. It is possible to give TAR after CDDP with minimal toxicity.

**ASSESSMENT OF RENAL FUNCTION DURING HIGH-DOSE *CIS*-PLATIN THERAPY IN PATIENTS WITH OVARIAN CARCINOMA.** P. K. BAUMAH, A. HOWELL, H. WHITBY E. S. HARPUR & A. GESCHER, *Depts. of Clinical Chemistry and Medicine, Queen Elizabeth Medical Centre, University of Birmingham, Dept. of Pharmacy, University of Aston, Birmingham*

Renal function was assessed in 22 previously untreated patients with Stage III/IV ovarian cancer treated with CDDP (100 mg/m$^2$) at 3-weekly intervals $\times$ 5. Hydration consisted of 3 l saline on Day 1 and 5 l saline and 1 l 10% mannitol with i.v. bolus CDDP on Day 2. Day 1 urine was collected for creatinine clearance and early-morning urine on Day 1 for $B_2$ microglobulin ($B_2M$) excretion and osmolality ($n = 22$). The brush-border enzyme alanine aminopeptidase (AAP) and the lysosomal enzyme N-acetyle-B-glucasaminidase (NAG) were estimated on concentrated urine specimens taken $\sim$34 h before and 38 and 62 h after CDDP bolus for 2 consecutive courses ($n = 7$).

Mean creatinine clearances (ml/min) before the first and fifth courses of treatment were 75·8 $\pm$ 25·6 (s.d.) and 76·8 $\pm$ 26·0. There was no change in mean urine osmolality and the mean $B_2M$ excretion fell from 143 to 86 mg/mmol creatinine. Pre-CDDP urinary NAG was raised in 5/7 and AAP in 4/7 patients. There was a tendency for pre-CDDP, AAP but not NAG excretion to rise with consecutive treatments ($P = 0·025$). Both NAG and AAP excretion increased at 38 h post CDDP and tended to fall by 62 h. In view of the absence of changes in conventional indices of deteriorating renal function it is not clear what significance may be attributed to the increased excretion of AAP and NAG in urine.

**THE ANTI-EMETIC POTENTIAL OF ORAL LEVONANTRADOL IN PATIENTS RECEIVING CANCER CHEMOTHERAPY.** J. F. B. STUART*†, J. WELSH*, G. SANGSTER*, M. SCULLION*, H. CASH‡, S. B. KAYE*, K. C. CALMAN*, **Pharmacy Department, University of Strathclyde, †Department of Oncology, University of Glasgow, ‡Clinical Projects Manager, Pfizer Ltd, Kent.*

Levonantradol a new anti-emetic which has no activity on the dopaminergic system, has shown to be effective in controlling nausea and vomiting caused by *cis*-platin chemotherapy. A dose-ranging study was made, using 0·25mg capsules of levonantradol in patients who had suffered from severe nausea and vomiting following different chemotherapeutic regimes despite adequate doses of conventional anti-emetics. Various doses of levonantradol were explored ranging from 0·25 mg 4-hourly to 1 mg of drug 4-hourly in 24 h after chemotherapy. 20 patients were studied who were receiving drugs such as *cis*-platin and cyclophosphamide, which are known to cause severe nausea and vomiting. The 20 patients were divided into 4 groups of 5. Five patients in Group 1 were given 0·25 mg of levonantradol and the remaining 3 groups of 5 received 0·5 mg, 0·75 mg and 1 mg respectively. Treatment with the drug was started 1 h before chemotherapy. The results obtained from the 4 different dose levels were as follows: only one patient in the 2 groups with 0·25mg and 0·5mg dose obtained $>50\%$ relief from nausea and vomiting. In the 0·75mg and 1mg group 7/10 patients obtained a $>50\%$ relief from nausea and vomiting, as compared with previous anti-emetic therapy. Toxicity due to the anti-emetic was mild but did show a dose relationship. As a result of these findings further studies at the 0·75mg and 1mg doses are planned, and alteration of dose schedules will be explored.

**EVALUATION OF NABILONE AS AN ANTI-EMETIC.** M. A. CORNBLEET, D. A. HAMILTON, P. CHRISTIAN & J. F. SMYTH, *Department of Clinical Oncology, Western General Hospital, Edinburgh*

Of the major side-effects of cytotoxic chemotherapy, nausea and vomiting remain among the most distressing. Conventional antiemetics are of only limited benefit and some patients find it impossible to continue treatment. Although the anti-emetic properties of marijuana are well established, its psychoactive properties make it unlikely to be widely acceptable. Nabilone is a synthetic cannabinoid in which a dimethylheptyl side-chain prevents conversion into $\Delta^9$ tetrahydrocannabinol, the psychoactive metabolite of marijuana. In a pilot study, nabilone has been assessed as an anti-emetic in 18 patients receiving cytotoxic chemotherapy. Twelve were treated with platinum-containing combinations, 4 received methotrexate and cyclophosphamide and 2 received adriamycin-containing combinations. All had previously experienced chemotherapy-associated nausea or vomiting. Nabilone was prescribed as 2mg capsules 6-hourly commencing 12 h before treatment, and then 2 mg bd for the duration of chemotherapy. Ten patients reported significant psychotropic effects of whom 6 recorded dysphoria and 4 euphoria. Five were sufficiently distressed to refuse a further trial of nabilone. Postural hypotension was observed in 1 patient, but no other side-effects were detected. 23% of patients experienced minimal or no nausea or vomiting but 77% still experienced moderate or severe symptoms. The high incidence (55%) of significant psychotropic side-effects make nabilone unlikely to be of routine value as an anti-emetic, particularly in out-patient practice. However a significant proportion of patients (39%) found chemotherapy more tolerable when treated with nabilone in this way.

### METOCLOPRAMIDE: DOSE-RELATED EFFECT ON THE EMESIS OF CHEMOTHERAPY.

R. Cox, C. E. Newman & M. J. Leyland, *East Birmingham Hospital and Newfoundland Cancer Treatment and Research Foundation*

The nausea and vomiting induced by chemotherapy is a major problem.

A randomized placebo-controlled trial of metoclopramide (M) was conducted in patients receiving chemotherapy for small-cell carcinoma of the lung. 10 mg of M or placebo was given i.v. or orally 4-hourly during treatment. The i.v. route was used for nauseated or vomiting patients. No vomiting occurred in 28/59 courses with M, compared with 10/59 with placebo. The difference is highly significant ($\chi^2 = 12{\cdot}17$, 1 df, $P < 0{\cdot}001$). In a further 39 courses in which M or placebo was given less than 4-hourly, no difference was noted.

It was thought that the anti-emetic effect of M could be dose-related. Groups of 5 patients undergoing chemotherapy were given 1–4 mg/kg/day of M i.v. in 6 divided doses. A further group of 9 patients have received 5 mg/kg/day. Only one episode of vomiting occurred and only one patient felt nauseated of these 9. Of 8 patients capable of eating all but 1 ate something of every meal whilst receiving M. One patient had moderate extra-pyramidal side-effects.

These preliminary data suggest that previous studies of metoclopramide have used it in inadequate doses, and that at high doses i.v. it is an effective anti-emetic.

### ASSESSMENT OF NUTRITIONAL SUPPLEMENTATION AND PLASMAPHERESIS IN CANCER PATIENTS.

G. E. Raines, J. M. Trotter, J. C. Willox, G. MacAuley & K. C. Calman, *Department of Clinical Oncology, Gartnavel General Hospital, Glasgow*

The effectiveness of enteral hyperalimentation and plasma exchange were investigated in cancer patients, examining in particular, albumin and urea metabolism, with the aim of improving their condition and determining which patients were likely to respond to nutritional support.

The distribution and rate of excretion or synthesis of the appropriate radioactive products, following i.v. injection of $^{131}$I-human serum albumin, sodium $^{125}$Iodide and sodium $^{14}$Carbonate, was monitored to determine albumin catabolism, synthesis and transcapillary escape rate and urea synthesis rate and half-life.

Of the patients studied to date with nutritional support, all had normal or high albumin synthetic, catabolic and transcapillary escape rates, in contradistinction to the previously reported low synthesis rates (Waldmann, *et al.*, 1963, *J. Clin. Invest.*, **42**, 171) and to the normal adaptation to protein-energy malnutrition. Most patients had normal urea-synthesis rates and half-lives, although 2 patients had low rates, despite their hypercatabolic state and nutritional support, and one patient studied before and during feeding showed a rise in urea-synthesis rate.

The benefits of plasma exchange were more obscure, though short-term clinical improvements were noted (Shaw *et al.*, 1980, *Br. Med. J.*, **281**, 1459). The changes in biochemical parameters and metabolism effected by the tumour were not ameliorated for greater than 12–24 h, except for a slight though statistically insignificant fall in urea-synthesis rate and a rise in transcapillary-escape rate. From these preliminary data it was concluded that a patient with net albumin synthesis was more likely to respond to nutritional supplementation and show an improved prognosis, than with a net catabolism.

### COMBINATION CHEMOTHERAPY IN ADVANCED GASTRIC CANCER.

D. CUNNINGHAM, D. C. CARTER, C. S. MCARDLE & M. SOUKOP, *Department of Medical Oncology and University Department of Surgery, Royal Infirmary, Glasgow*

The median survival of patients with advanced gastric cancer is 4 months. Recent reports of combination chemotherapy have been encouraging. We thus undertook a prospective Phase II study of 28 consecutive patients with inoperable or meastatic gastric cancer treated with intravenous 5-Fluorouracil, Adriamycin and Mitomycin-C (F.A.M).

Seventeen patients (61%) failed to respond (median survival 2·5 months). In 11 patients (39%) a partial response was obtained, their median survival being 9 months; 9 are still alive. They included 2 patients whose cancer became resectable after cytotoxic therapy and a further patient with dysphagia in whom the need for intubation was avoided. Apart from alopecia, the treatment was well tolerated. Before therapy, performance status was similar in both groups; all responders subsequently showed an improvement in performance status.

This study suggests FAM prolongs survival and may permit secondary resection in patients initially considered to be inoperable.

### ORAL CANCER EPIDEMIOLOGY IN SCOTLAND.

P. BOYLE*‡, C. SCULLY† & C. R. GILLIS‡, **IARC, Lyon; †University Department of Oral Medicine and Pathology, Glasgow and ‡West of Scotland Cancer Surveillance Unit, Glasgow*

The epidemiology of oral cancer in Scotland appears not to have been studied, and it is now 10 years since the last published major study in England and Wales (Binnie *et al.*, 1972, *OPCS Studies on Medical and Population Subjects No.* **23**, HMSO). We have therefore examined mortality from oral cancer in Scotland since 1911 and incidence since 1963. The all-ages age-standardized mortality rates for oral cancer fell during the 67 years as did the rates for those subsites for which data were available—lip, tongue and rest of mouth. Similar declines in mortality were observed in both sexes, these mirroring the falls in incidence. Falls in mortality and incidence were observed in all age groups. A strong birth-cohort effect was found for all subsites, with each cohort born in 10-year periods subsequent to that centred on the year 1878 experiencing declining levels of mortality from oral cancer. In view of the strength and consistency of the demonstrated association between oral cancer and the use of tobacco, this decline is surprising, and suggests the presence of other aetiological factors of more importance in the Scottish environment.

### PROGNOSTIC FACTORS IN HODGKIN'S DISEASE.

G. VAUGHAN HUDSON & A. M. JELLIFFE, *The British National Lymphoma Investigation*

During the last 12 years many patients with Hodgkin's Disease have been referred to the B.N.L.I. It has become apparent that various pretreatment factors, including clinical stage, symptoms, age, histology, ESR, lymphocyte count, haemoglobin, and albumin, can be related to the tempo of the disease and thus to the prognosis of the individual case and need for more vigorous treatment.

Factors associated with a poor prognosis appear to be advanced clinical stage, "B" symptoms (weight loss, fever, night sweats), older age group (over 45), poor histology (including recently recognized Grade II nodular sclerosis), high ESR, and low peripheral-blood values for lymphocytes, haemoglobin and albumin.

The provisional results of a detailed analysis of 1600 cases are presented with reference to is possible implications for staging and management.

### POSTER PRESENTATIONS

### IMPLICATIONS OF GOMPERTZIAN GROWTH OF TUMOURS FOR RATE OF DECLINE OF CLONOGENIC CELL NUMBER DURING SINGLE-AGENT CHEMOTHEARPY.

T. E. WHELDON*, R. BELL† & G. F. BRUNTON‡, **MRC Cyclotron Unit*, *†Department of Medical Physics, Hammersmith Hospital, London W12 and ‡Dept of Clinical Physics and Bio-engineering, Glasgow G4 9LF*

Gompertzian growth of tumours may be due to a decreasing growth fraction, an increasing cell-loss factor, or both. When Gompertzian retardation is primarily due to a changing growth fraction, cycle-specific chemotherapy is increasingly efficient in killing clonogenic tumour cells, more of these cells being called into cycle as the tumour regresses and the growth fraction expands. However, when retardation is largely due to a changing loss factor, there is no increase in the vulnerability of clonogenic cells during regression, but, as the net specific growth rate increases, regressing tumours may display "kinetic resistance", the rate of regrowth increasing until it balances the rate of cell kill. Kinetic resistance may also be displayed by tumours conforming to Gompertz growth kinetics (of either type) when treatment is with a non-cycle-specific drug. This may provide a mechanism for the failure of long-term chemotherapy to achieve cure, in some cases where short-term chemotherapy using the same agent is seen to cause tumour regression.

### STRUCTURE-ACTIVITY STUDIES WITH THE HOMOLOGOUS SERIES OF CROSSLINKING DIMETHANE-SULPHONIC ACID ESTERS.

P. BEDFORD & B. W. FOX, *Paterson Laboratories, Christie Hospital and Holt Radium Institute, Manchester M20 9BX*

The ability of members of the homologous series of dimethanesulphonic acid esters ($n = 1$–9) to crosslink the DNA of cells derived from the transplantable rodent Yoshida Lymphosarcoma, was assayed by the technique of alkaline elution described elsewhere (Bedford & Fox, 1982, *Chem. Biol. Interact.*, **38,** 119). A peak of interstrand crosslinking occurred after treatment of the cells with hexane dimethanesulphonate ($n = 6$) which decreased with shorter, or longer alkylating chain lengths. The peak of crosslinking activity by the 6-carbon chain member was paralleled by its optimal cytotoxicity towards the Yoshida cells in culture. Ethylene dimethanesulphonate ($n = 2$) produced no detectable interstrand crosslinks, which was reflected in a lack of *in vitro* cytotoxicity and *in vivo* antitumour activity. A possible relationship between DNA–DNA interstrand crosslinking ability and cytotoxicity throughout the series was obtained, which was partially but not wholly reflected in the antitumour effectiveness of members of the series. Preliminary data suggest however that the action on haemopoietic and spermatogenic systems may not correlate with DNA–DNA interstrand crosslinking in the same way.

### ASPECTS OF ANALYSIS, FORMULATION AND PHARMACOKINETICS OF 1,3,5 - TRILGLYCIDYL - S - TRIAZINE-TRIONE (αTGT) AFTER I.V. ADMINISTRATION.

M. N. AZMIN*†, J. F. B. STUART*†, A. SETANOIANS*, R. G. G. BLACKIE*, D. WHITEHILL*, J. WELSH*, **Department of Oncology, University of Glasgow and †Department of Pharmaceutics, Strathclyde University*

The search for new cytotoxic agents led to the discovery of αTGT, a tri-epoxide derivative shown to have antineoplastic activity in several murine tumours including the cyclophosphamide-resistant P388 murine tumour lines. Recent Phase I clinical trials have suggested that αTGT may be useful in treatment of human malignancies. Since this drug is intended for parenteral administration, the final dosage form must be sterile and have an acceptable level of purity and stability. The poor aqueous solubility and stability of αTGT are the added problems which have to be taken into account during the preparation of the injectable formulation. About 10–13 mg αTGT dissolved in every ml of water. αTGT in water is broken down to 3 degradation products, and the process is accelerated by an increase in temperature. Breakdown of dry αTGT was also evident after exposure to γ-irradiation. Since heat and γ-irradiation could not be used, filtration is the only viable method for sterilization though about 20% of the drug may be lost in the process. The sterile filtrate was freeze-dried to the final form, which is stable for at least 2 months when stored at 4°C. The aqueous solubility of αTGT was doubled or trebled by the addition of non-ionic surfactants. Apart from reducing the bulkiness of αTGT solution to 1/2 or 1/3 prior to filtration and freeze-drying, the surface-active agent added was also found to reduce the rate of αTGT degradation. Of the i.v. infusion fluids studied, only 5% dextrose solution was found to be suitable as a vehicle for αTGT. 0·9% sodium chloride injection on the other hand enhanced αTGT breakdown.

### DEFICIENCY OF 3-HYDROXYBUTYRATE UTILIZATION IN TUMOURS.

M. J. Tisdale & R. A. Brennan, *C.R.C. Experimental Chemotherapy Group, Department of Pharmacy, University of Aston, Birmingham B4 7ET*

Ketone bodies (3-hydroxybutyrate, acetoacetate and acetone) are an important metabolic fuel for peripheral tissues during starvation. Utilization of 3-hydroxybutyrate requires the presence of a 3-oxoacid CoA transferase, whereby the CoA moiety of succinyl CoA is transferred to the oxoacid thereby providing a precursor for the formation of acetyl CoA. This enzyme is present in variable amounts in all normal tissues except liver, but is virtually absent from a range of murine and human tumours, which are unable to utilize 3-hydroxybutyrate *in vitro*. In contrast with the loss of 3-oxoacid CoA transferase, the activity of 3-hydroxybutyrate dehydrogenase is comparable in tumour and normal tissue. This suggests that such tumours may be unable to maintain their ATP levels under conditions in which ketone bodies are the predominant energy source. Such a situation could be achieved *in vivo* by feeding patients a low carbohydrate diet supplemented with high levels of 3-hydroxybutyrate, together with an inhibitor of gluconeogenesis from lactate. In view of the ability of 3-hydroxybutyrate to feedback-inhibit breakdown of muscle and adipose tissue, such a treatment would also be expected to alleviate the wasting syndrome sometimes associated with advanced cancer.

### THE SITE OF ACTION OF AMINOGLUTETHIMIDE (AG) IN ADVANCED BREAST CANCER.

A. L. Harris*, M. Dowsett†, S. Jeffcoate†, I. E. Smith*, **Royal Marsden Hospital, Fulham Road, London and †Endocrinology Department, Chelsea Hospital For Women, London*

AG is an effective drug in advanced postmenopausal breast cancer. It inhibits the conversion of cholesterol to androgens in the adrenal glands (desmolase) and also the conversion of androgens to oestrogens peripherally in adipose tissue and breast carcinoma (aromatase). It is combined with replacement doses of hydrocortisone.

Short Synacthen tests (250 μg Synacthen i.m.) were performed on 10 post-menopausal women with advanced breast cancer who had received AG (250 mg 4 × day) plus hydrocortisone (20 mg 2 × day) for at least 3 months. There was no significant rise in cortisol, oestrone or dehydroepiandrosterone sulphate. However, 17OH progesterone (17OHP), $\Delta^4$ androstenedione, ($\Delta^4$A) and dehydroepiandrosterone rose markedly. 17OHP rose by up to 53-fold, and $\Delta^4$A up to 10·8-fold. This pattern of response to ACTH suggests that there is little inhibition of desmolase by AG, but that inhibition of aromatase is more important, and even a marked rise in precursors does not override aromatase inhibition. Since conventional dose AG is given to inhibit desmolase we used low dose AG without hydrocortisone to inhibit aromatase, which *in vitro* is more sensitive than desmolase. Twelve postmenopausal patients were given AG (125 mg 2 × day) for 1 week and in all patients oestrone fell (50 × 19% of baseline). AG was more than doubled each week until 500 mg 2 × day. Oestrone at the highest dose of AG was 54 ± 28% of baseline. The addition of 20 mg hydrocortisone 2 × day did not further suppress oestrone (55 ± 25% of baseline). This suggests that adrenal suppression by hydrocortisone does not contribute to the effect of AG, and that low-dose AG alone should be assessed for therapeutic effect.

### INCREASED ANTITUMOUR ACTIVITY OF PLATINUM DRUGS IN COMBINATION WITH PREDNISOLONE.

P. M. Goddard, C. R. Shepherd & K. R. Harrap, *Dept Biochem. Pharmacol., Inst. Cancer Res., Sutton, Surrey*

In experimental tumour systems, prednisolone potentiates the antitumour activity of some alkylating agents by reducing host toxicity and increasing tumour-cell kill, particularly against alkylating-agent-resistant tumours. The clinical efficacy of alkylating agents is also increased by their use in combination with prednisolone. The modes of action of alkylating agents and platinum drugs are very similar: both cross-link DNA and interact with nuclear proteins. Alkylating-agent-resistant Walker and Yoshida tumours are cross-resistant to platinum compounds, and we now report increased antitumour activity of platinum/prednisolone combinations. Using both s.c. and i.p. routes, prednisolone, 4 h after either cisplatin (*cis*-dichloro diammine platinum (II)) or CHIP (*cis*-dichloro, *trans*-dihydroxy *bis*-isopropylamine platinum IV), resulted in a doubling of the therapeutic index against the alkylating-agent-sensitive Walker tumour carried in Wistar rats. Prednisolone alone is not active against this tumour. The host toxicity of the combination was identical to that of the platinum drug alone, but the $ED_{90}$ was halved. An increased cell kill of the alkylating-agent-resistant tumour was also obtained using platinum/prednisolone combinations. These data suggest that therapeutic benefit may be obtained from the use of prednisolone in combination with platinum drugs in the clinic even for non-steroid responsive tumours.

### MODIFIERS OF DRUG METABOLISM: THEIR EFFECTS ON THE RESPONSE OF TUMOUR AND NORMAL TISSUES TO CYTOTOXIC AGENTS.

P. Workman & P. Twentyman, *MRC Clinical Oncology and Radiotherapeutics Unit, Cambridge*

We have investigated the effects of the hepatic microsomal enzyme-inducer phenobarbitone and the inhibitor SKF 525A (proadifen HCl) on the response of the RIF-1 and KHT solid tumours to cytotoxic agents in mice. Effects on peripheral white-blood cells and $LD_{50}$ were also determined. SKF 525A enhanced tumour response to chlorambucil and CCNU but not melphalan. Normal-tissue responses indicated a therapeutic gain with CCNU but not chlorambucil. Phenobarbitone reduced both tumour and normal-tissue responses to CCNU and chlorambucil, but the effects on therapeutic ratio were complex. Studies are in progress with cyclophosphamide.

The effects of SKF 525A on cytotoxic drug response are very similar to those with the nitroimidazole misonidazole, which is under investigation as a chemosensitizer for clinical use. For a range of misonidazole analogues, structure-activity relationships for chemosensitization were closely similar to those for enhancement of pentobarbitone sleep-time, used to assay inhibition of drug metabolism.

We conclude: (1) response to cytotoxic drugs can be altered by modifiers of drug metabolism, and this can result in changes in therapeutic ratio; (2) inhibition of drug metabolism appears to represent a major component of the *in vivo* chemosensitization mechanism.

### CHEMOTHERAPY OF A NEW WELL DIFFERENTIATED TRANSPLANTABLE MOUSE ADENOCARCINOMA OF THE COLON (MAC 30/T).

J. A. Double & M. C. Bibby, *Clinical Oncology Unit, University of Bradford, Bradford BD7 1DP*

The MAC series (Double *et al.*, 1975, *J. Natl Cancer Inst.*, **54,** 271) has been used in a variety of chemotherapy studies. MAC 30/T was regrown from MAC 30 (Cowen *et al.*, 1980, *J. Natl Cancer Inst.*, **64,** 675) after storage in liquid $N_2$. The tumour is a well-differentiated mucoid adenocarcinoma which has remained histologically unaltered in a subcutaneous serial passage in NMRI mice for 31 months. The epithelium of the tubules contains goblet cells with periodic acid–Schiff and Alcian Blue positive contents.

The anti-tumour activity of a series of standard agents against this line has been determined. Antitumour activity was measured by growth delay from semi-log plots of relative tumour volumes calculated from serial calliper measurements. The growth of control tumours has remained consistent throughout the course of these experiments, with a mean volume doubling time of 3·4 days. Like other tumour lines within this series responses are only seen close to maximum tolerated dose. Results to date indicate that the best responses are seen with the alkylating agents Methyl CCNU and

Cyclophosphamide, where tumour-volume-doubling can be delayed by as much as 16 days. The antimetabolite 5FU at MTD however, produced a delay of less than 3 days.

A tumour exhibiting these response characteristics with different agents would seem a useful model for further studies, particularly in the area of combination chemotherapy.

**COMBINATION CHIP-X-RAY TREATMENT OF THE $C_3H$ MOUSE MAMMARY ADENOCARCINOMA,** M. PENHALIGON, M. LAVERICK & A. H. W. NIAS, *Richard Dimbleby Department of Cancer Research, St. Thomas's Hospital Medical School, London SE1 7EH*

CHIP (*cis*-dichlorobis(isopropylamine)*trans*-dihydroxy platinum (IV)) has previously been shown to enhance the radiation response of hypoxic C3H mouse mammary adenocarcinoma cells *in vitro*. The enhancement ratio was 2·1 using 60 $\mu$g/ml 1 h before X-rays (Laverick & Nias, 1981 *Br. J. Radiol.*, **54**, 529). *In vivo* combination studies were made using the C3H mouse mammary adenocarcinoma since it has a high hypoxic fraction and is radioresistant ($TCD_{37}$ 68·8 Gy in air, Tozer, 1981, *Br. J. Radiol.*, in press). We use SPF-derived C3H mice in which the $LD_{50}$ of CHIP is 65 mg/kg. Unanaesthetized mice were given the maximum tolerated single dose of CHIP, 40 mg/kg, i.p. either 30 min, 1 or 3 h before a single dose of 50, 70 or 90 Gy.

When the experiment was repeated, one group of animals received 40 mg/kg of the protective agent WR 2721 30 min before CHIP in order to raise its MTD to 90 mg/kg. Tumours were then treated with X-rays (70 Gy) 1 h later. Tumour response was assessed by both regrowth delay and cure ($TCD_{37}$ by 80 days).

**ENHANCEMENT OF RESPONSE OF A LYMPHOBLASTIC TUMOUR BY COMBINATION OF THE CYCLE SPECIFIC DRUGS CISPLATIN AND TREOSULFAN.** A. W. PREECE & M. WELLS-WILSON, *Radiotherapy Centre, Bristol BS2 8ED*

Clinical interest in the treatment of solid tumours, particularly ovarian cancers, with combinations of active agents has led to the investigation of these agents in animal tumour lines. The L2C lymphoblastic tumour in strain 2 guinea-pigs has been proposed as a useful model for testing chemotherapeutic regimens (Murphy, 1978 in *Immunological Parameters of Host-Tumour Relationships*, **5**, 20). Chemotherapy is administered when lymphoblastoid cells appear in the blood (about 8 days after implantation).

Varying doses of treosulfan, cyclophosphamide, cisplatin and prednisolone were administered. Prednisolone had no effect on tumour growth, either alone or in combination with cyclophosphamide or treosulfan. Moderate doses of treosulfan or cyclophosphamide produced temporary remissions, but cisplatin was less effective. Combinations of cisplatin and treosulfan produced longer remissions than either agent alone, with no evidence of increased toxicity, which might have been predicted by drug classification.

These studies may indicate potentially useful chemotherapeutic combinations for clinical adoption.

**PHASE II TRIAL OF 5-FLUOROURACIL IN DIFFUSE MALIGNANT MESOTHELIOMA.** V. J. HARVEY, M. L. SLEVIN, B. A. J. PONDER & P. F. M. WRIGLEY, *ICRF Dept of Medical Oncology, Hackney and St Bartholomew's Hospitals, London*

Malignant mesothelioma is a rare tumour. Although predominantly a localized tumour, long-term survival following surgery or radiotherapy is uncommon. Assessment of the value of chemotherapy has been limited. FU has been reported to be active in mesothelioma in a total of 3/8 patients in 3 different studies. Our experience with 1 patient who had a dramatic response to FU led us to conduct a Phase II trial of this agent. Adriamycin is currently the most active agent tested with 16/36 patients responding. We have therefore treated patients who relapse or progress on FU with adramycin in a second Phase II study. Since 1978 we have treated 18 consecutive patients with a confirmed diagnosis of diffuse malignant mesothelioma of the pleura or peritoneum with FU as a single agent (550 mg/$m^2$ daily $\times$ 5 q 28 days). Seventeen patients had pleural mesothelioma, and 1 patient had peritoneal mesothelioma. There were 12 males with a median age of 56 years and 6 females with a median age of 41 years.

No responses were seen in the 18 patients receiving FU and the only patient responding to adriamycin (1/8) was the patient who had responded initially to FU. The median survival was 5½ months and the longest survival amongst the non-responders was 15 months. The 1 responder is alive and has recently relapsed at 5½ years. We conclude that FU has only minimal activity in diffuse malignant mesothelioma. Our experience with adriamycin is not yet sufficient to make definite conclusions, but to date little activity as a second-line agent has been demonstrated.

**A COMPARISON BETWEEN A SINGLE-AGENT SHORT-COURSE CHEMOTHERAPEUTIC REGIMEN AND A QUADRUPLE PROLONGED-COURSE REGIMEN, FOR SMALL CELL BRONCHOGENIC CARCINOMA OF LIMITED EXTENT.** K. B. CARROLL, H. MOUSSALLI & N. THATCHER, *Regional Cardiothoracic Unit, Wythenshawe Hospital, Manchester*

68 patients with inoperable but "limited" stage small-cell carcinoma of the bronchus were treated with 2 different chemotherapeutic regimens. 34 patients received methotrexate, cyclophosphamide, procarbazine and vincristine in standard dose over a 2-year schedule. 34 patients received cyclophosphamide alone over a period of 3 months. The groups were evenly balanced with respect to clinical details which are, fully described. The toxicity was acceptable.

The median actuarial survival for the whole group was 11 months, the 1-year, 2-year and 4-year survival being 48%, 30% and 1·5% respectively. There was no statistically significant difference in survival between those classified as responders and those as non-responders, nor between the groups treated with the 2 different regimens. There was a high local-recurrence rate.

**METASTATIC MEDIASTINAL TERATOMA.** D. PARKER, E. S. NEWLANDS, G. J. S. RUSTIN, R. H. J. BEGENT & K. D. BAGSHAWE, *Department of Medical Oncology, Charing Cross Hospital, Fulham Palace Road, London W6 8RF*

Malignant mediastinal teratoma has been regarded as inevitably incurable. By contrast, 4/7 patients referred to our unit have survived for between 12 and 123 months in remission after a combination of chemotherapy and surgery. The 3 factors preventing success in the 3 patients who died were: failure of previous therapy before referral; severe respiratory embarrassment due to breakdown of extensive disease on starting therapy, and drug resistance in a patient with a yolk sac element in the teratoma.

Our experience suggests that successful treatment depends upon (1) the use of drug combinations including *cis*-platin at an early stage (Newlands *et al.*, 1980, *Br. J. Cancer*, **42,** 378), (2) moderation of initial drug dose if there is very extensive disease, (3) the use of tumour markers to calculate the optimal time for thoracotomy after chemotherapy.

**HYPOVITAMINOSIS C IN LUNG CANCER.** H. M. ANTHONY & C. J. SCHORAH, *University Departments of Immunology and Chemical Pathology, Leeds General Infirmary*

In a study involving 158 samples from 139 lung cancer patients, 64% had plasma vitamin C values below 0·3 mg% and 25% had buffy-coat levels below 10 $\mu$g/$10^8$ cells, the thresholds for incipient clinical scurvy. Vitamin C levels were diet-dependent and could be increased by oral supplementation. Compared with control values established in this laboratory, levels were low both in tumour-bearing patients and in those clinically free of disease after resection, particularly in the first 6 months.

The expected tendency for buffy-coat vitamin C to correlate with the proportion of lymphocytes in peripheral blood was only apparent in "unreactive" patients. Patients with relative lymphocytosis ($\geqslant$25%) tended to show an inverse relationship, significant in the group clinically free of disease, in many of whom subclinical recurrence is likely. A link between lower mononuclear-cell vitamin C, higher lymphocyte counts and resectability was noted in 14 samples taken on diagnosis, in which the former was measured directly.

The vitamin C content of 13 surgical specimens of primary lung tumours was assayed: tumours had higher vitamin C content (mean 67·7 ± 33·4 mg/g tissue) than normal lung (35·5 ± 12·4 mg/g tissue).

The data cannot be explained solely by preferential accumulation of vitamin C in tumour tissue in patients with inadequate vitamin C intake: it appears that vitamin C utilization in repair after major surgery and in resistance to lung cancer also contributes.

**4′ EPI-DOXORUBICIN IN LEUKAEMIA AND LYMPHOMA (A PRELIMINARY CLINICAL TRIAL).** S. ERIDANI, A. KUBIE & F. LUNGU, *Department of Haematology, St Thomas' Hospital, London*

4′ Epi-Doxorubicin (EDX) is a stereoisomer of doxorubicin with a different configuration in the sugar moiety. After early results in solid tumours (Bonfante *et al.*, 1979, *Cancer Treat. Rep.*, **63,** 915) we have started a clinical trial in leukaemia and non-Hodgkin's lymphoma, substituting EDX for daunorubicin and doxorubicin in combination regimes.

Thirteen patients so far have been treated: 6 cases of previously untreated acute leukaemia at presentation (4 myeloblastic, 1 monoblastic, 1 lymphoblastic), 1 case of undifferentiated acute leukaemia in full relapse after 2 years' remission, 1 case of multiple myeloma also in relapse, 5 cases of non-Hodgkin's lymphoma either in incomplete remission or relapse, all treated previously. The age range was 18–64, with a prevalence of the over-50's. Patients were unselected; in particular no exclusion was made on the basis of short life expectancy. In the acute leukaemia group at presentation there was a variable response: 3 complete and 1 partial remission, and 1 failure. An additional failure was seen in the patient with full-blown relapse. All 5 patients with lymphoma achieved complete remission, and in the case of multiple myeloma, a favourable response. The highest dose, of EDX was 620 mg, (1 case) including 180 mg doxorubicin given previously. Side effects were limited: occasional nausea, hair loss in 1 case, depression in a few cases. No signs of cardiac impairment were found either on clinical or laboratory evidence.

If confirmed, these results could indicate that EDX has similar activity to other antracyclines, but with less cardiac and general effects

**I.V. TREOSULFAN IN ADVANCED OVARIAN CANCER: A MULTI-CENTRE PILOT STUDY.** U. ABDULLA*, H. H. MAKANJI*, C. COX†, T. K. ALSAIDI†, W. F. WHITE‡ & J. E. MASDING‡, **Department of O & G, Royal Liverpool Hospital, †Department of O & G, Walsgrave Hospital, Coventry and ‡Radiotherapy Centre, St Luke's Hospital, Guildford*

A formulation of treosulfan for i.v. use has recently been made available for investigation. From December 1979 to September 1981, 22 patients with inoperable, histologically confirmed, ovarian cancer have been treated with this preparation as first-line chemotherapy at 3 centres.

The usual dose regimen was 5–15 g either by bolus i.v. injection or i.v. infusion, repeated at 2–3-weekly intervals depending on blood counts; a marked variation in individual tolerance to treosulfan was found. Of 18 fully evaluable patients at March 1982, there were 14 responses (78) of a minimum duration 3 months (range 4–28+ months), 8 of which were complete (CR), mainly judged on clinical evaluation. "Second-look" operations were not routine, but were carried out in 4 instances: 1 CR was confirmed, and 2 PRs underwent radical surgery, though both relapsed, at 2 and 9 months following operation.

Dose-limiting toxicity was leucopenia (leucocytes $<2500/mm^3$ in 50%) and thrombocytopenia (thrombocytes $<100{,}000/mm^3$ in 45%). Single instances of vomiting were observed in 2 patients, and some hair loss in 1 patient.

I.v. treosulfan is an effective treatment for ovarian cancer, enabling a response to be obtained in a high proportion of patients. The formulation is suited to combination with active agents in this disease in view of moderate toxicity when carefully monitored, and excellent patient acceptability.

**IMPORTANCE OF CYTOPLASM/CELL MEMBRANE DAMAGE INDUCED BY ADRIAMYCIN.** J. M. WALLING & M. J. ORD, *Department of Biology, University of Southampton, Hants. SO9 3TU*

Adriamycin has been shown to have a variety of cellular effects besides intercalation into DNA, including an inhibition of mitochondrial enzymes (Iwamoto *et al.*, 1974 *Biochem. Biophys, Res. Commun.*, **58,** 633) and an interaction with membrane phospholipids including cardiolipin (Goormagtigh *et al.*, 1980, *Biochim Biophys Acta*, **597,** 1). In the present study Adr was found to have a biphasic pattern of toxicity towards *A. proteus* and CHO cells. Cells were either killed very rapidly as a result of complete loss of membrane integrity and consequent organelle destruction, or more slowly over a period of up to 8 days following removal of the treatment solution as assayed by single-cell cloning.

EM studies on amoebae showed aberrant mitochondrial profiles with a reduction in the number of internally located cristae and a widening of the peripherally located cristae. Very aberrant mitochondria were eventually sequestered in autophagocytic vacuoles. The typical anthracycline-induced nucleolar fragmentation was often seen in later stages of pathogenesis. However, this was never observed without concomitant cytoplasmic damage. The results of an initial series of nuclear transfers between treated and control amoebae suggest that treated cytoplasms have a far poorer ability to survive than treated nuclei.

Taken together the results with both amoebae and CHO cells suggest that damage to the cytoplasm/cell membrane may contribute considerably to Adr-induced cell death.

### EFFECT OF ADRIAMYCIN ON MEMBRANE POTENTIAL OF HUMAN ERYTHROCYTES.

S. B. Chahwala, J. A. Hickman & R. G. Grundy, *C.R.C. Experimental Chemotherapy Group, Department of Pharmacy, University of Aston, Birmingham B4 7ET*

The antitumour activity of adriamycin (Adr) is generally considered to be related to its ability to intercalate DNA, though other targets have been proposed, particularly the cell membrane (Tritton *et al.*, 1976, *Biochem. Biophys. Res. Commun*, **84,** 802). Dasdia *et al.* have reported that $10^{-7}$M Adr altered ion flux in HeLa cells and they suggested that these changes may be relevant to cytotoxicity, presumably since changes in alkali metal and calcium ion fluxes are associated with control of cell growth (*Pharmacol. Res. Commun.*, 1979, **11**).

We have studied the effects of Adr on the membrane potential ($\Psi$) (a refiection of complex changes in ion flux) of human RBCs, a cell type used previously as a model for the study of Adr interaction with membranes (Mikkelsen *et al.*, 1977, *J. Molec. Med.* **2,** 33). Measurements of $\Psi$ were obtained by following the accumulation of the lipophilic cation triphenyl methylphosphonium bromide ($TPMP^+$) into fresh RBCs. Resting $\psi$ was $9{\cdot}98 \pm 1{\cdot}09$ mV (n = 4) which agrees with that obtained by other methods. Adr ($10^{-7}$M) had no effect on $\Psi$ after 1 h. Adr ($10^{-4}$M) for 1 h induced a hyperpolarization with $\Delta\Psi$ of $25 \pm 2$ mV (n = 3). The lack of effect of $10^{-7}$M Adr on $\Psi$ of RBCs may be explained by supposing that $10^{-7}$M Adr has little effect on the $Cl^-$ Donnan equilibrium, which predominantly governs RBC $\Psi$ (Ouabain, an inhibitor of $Na^+K^+$ATPase ($10^{-4}$M), similarly had no effect). The effects of $10^{-4}$M Adr may have been induced *via* gross morphological changes observed by us and Mikkelsen *et al.*, resulting in the disturbances of the $CL^-$ equilibrium.

### THE USEFULNESS OF HUMAN PROSTATE TUMOUR-CELL LINES IN THE STUDY OF CHEMOSENSITIVITIES.

S. A. Metcalfe, J. R. W. Masters & B. T. Hill, *Institute of Urology and Imperial Cancer Research Fund Laboratories, London*

We are using cell lines derived from human prostate carcinoma to evaluate *in vitro* methods for predicting response of tumours to anticancer agents. Logarithmically growing cells were exposed to drugs for 24 h and their viability assessed by measuring growth rates and clonogenicity in agarose. On the basis of growth rates, PC3mA2 and DU145 cells showed a greater sensitivity to a range of drugs than PC3 cells, possibly reflecting the longer population-doubling time of the PC3 line. When cloned in agarose (0·17%) the CFEs were ~15, 4 and 0·2% for PC3mA2, DU145 and PC3 respectively, so only PC3mA2 and DU145 cells have been used to study chemosensitivity based on colony-forming ability. Using this assay, PC3mA2 cells were more sensitive than DU145 cells to several drugs, including *cis*-platinum ICRF-159, dibromodulcitol and methotrexate; though some drugs (*e.g.* FU), had a similar effect on both lines. Survival data with DU145 cells for these drugs were similar to those described using a human neuroblastoma line, CHP100 (Hill & Whelan, 1981, *Pediatr. Res.*, **15,** 1117) and a human colon carcinoma line, LoVo (Drewinko *et al.*, 1981, *Cancer Res.*, **41,** 2328, treated for 24 or 1 h respectively. Certain antitumour drugs thus appear more effective in killing PC3mA2 prostate carcinoma cells. Preliminary studies are underway to assess the response of human prostate tumour biopsy material from individual patients to chemotherapy, using the human "stem" cell assays.

### *IN VIVO* CONCENTRATION AND TIME INTER-RELATIONSHIPS FOR ANTI-CANCER DRUG CYTOTOXICITY TO HUMAN OVARIAN CARCINOMA CELLS.

H. T. RUPNIAK & B. T. HILL, *Laboratory of Cellular Chemotherapy, Imperial Cancer Research Fund, London WC2A 3PX*

We have studied the drug sensitivity of cryopreserved tumour cells obtained from the ascitic fluid of a patient with ovarian carcinoma who had relapsed following treatment with chlorambucil. Tumour-cell survival of drug treatment *in vitro* was assessed by measurement of colony-forming ability using the Courtenay assay (Courtenay *et al.*, 1978 *Br. J. Cancer*, **38,** 77). The anticancer drugs *cis*-platinum (*cis*-Pt) and adriamycin (Adr) generated exponential survival curves, wherein increasing time of exposure increased degrees of cell kill. For *cis*-Pt, the doses required to reduce tumour cell survival to 10% ($D_{10}$) after 1, 6, 18 h and continuous exposure were 14·6, 3·3, 0·7 and 0·18 μg/ml respectively. The $D_{10}$ values for treatment with Adr for 1, 6, 18 h and continuously were 1·1, 0·38, 0·19 and 0·03 μg/ml respectively. In contrast, treatment with hydroxyurea (HU) in concentrations up to 1 mg/ml for either 1 or 6 h elicited no detectable cell kill. However, when HU was incorporated into the agar cloning system, producing continuous drug exposure, an exponential survival curve resulted with a $D_{10}$ value of 28 μg/ml. Exposure to methotrexate in concentrations up to 10 μg/ml for 1 h also resulted in no detectable cell kill, though this may be partly attributable to protection/rescue by dT present in the Ham's F12 medium used for these studies. The patterns of response to drug treatment are in essential agreement with most studies in tumour cell lines but appear inconsistent with some of the data derived from the alternative Hamburger & Salmon assay (Hamburger *et al.*, 1978, *Cancer Res.*, **38,** 3438).

### THE ACTION OF 6-MERCAPTOPURINE (MP) NUCLEOTIDE "PRODRUGS" ON MP-RESISTANT CELLS.

D. M. TIDD, H. P. J. JOHNSTON & I. GIBSON, *University of East Anglia, Norwich, Norfolk NR4 7TJ*

Purine and pyrimidine antimetabolite-resistant cells with reduced drug nucleotide-forming capacity may be inhibited by so-called "prodrugs" of the analogue nucleoside 5′-monophosphate. It has been suggested that this is due to intracellular uptake of intact prodrug molecules and their subsequent hydrolysis to the free nucleotide. We report that *bis*-(6-mercaptopurine-9-β-D-ribofuranoside)-5′,5‴-monophosphate (*bis*-(MPR)P) and its butyryl derivative, *bis*-(O³'-dibutyryl-6-mercaptopurine-9-β-D-ribofuranoside)-5′,5‴-monophosphate (*bis*-(dibutyrylMPR)P) inhibited growth of thiopurine-resistant L1210/MPR cells in culture with $EC_{50}$ values of 580 μM and 42 μM respectively, whilst 1mM MP riboside (MPR) had no effect. *Bis*- (dibutyryl-MPR)P was less readily broken down to MPR by serum enzymes than *bis*-(MPR)P, and cells did not contribute to its extracellular degradation. The breakdown product, MPR, was responsible for the delayed cytotoxicity of the pro-drugs on MP-sensitive L121/0 cells. *Bis*-(MPR)P elicited a delayed cytotoxicity in L1210/MPR cultures, in keeping with is proposed action as a prodrug of MPR 5′- monophosphate, whilst *bis*-(dibutyrylMPR) Pinduced acute growth inhibition and no delayed cytotoxicity in the resistant subline. However, MPR was incorporated into L121/O) DNA as 6-thioguanine deoxyribonucleotide, whilst *bis*-(MPR)P was not incorporated into L1210/MPR DNA. These results suggest that the action of the prodrugs may not represent true circumvention of MP-resistance.

**CHROMOSOME DAMAGE IN LEWIS LUNG (LL) TUMOUR LINES WITH A SPECTRUM OF SENSITIVITIES TO MeCCNU.** J. H. Peacock, G. Casey, T. J. McMillan & T. C. Stephens, *Institute of Cancer Research, Sutton, Surrey*

In the course of a study to explore the development of resistance to the chemotherapeutic drug methyl CCNU (MeCCNU) in wild-type LL we have derived several tumour lines varying widely in sensitivity to this drug. These lines could have arisen either by selection of cells surviving treatment or by genetic or epigenetic changes induced by the treatment. Initially, we have studied the chromosomal content of some of these lines to look for specific genetic differences.

Five lines, wild-type LL, a line designated R4/1 (resistant to MeCCNU) and 3 closely related lines R10 (MeCCNU sensitive), R10/1 (resistant) and R10/4 (sensitive) were karyotyped. The modal chromosome number varied slightly between the lines ($\sim 70$); however the most striking difference was the presence of only 2 metacentric marker chromosomes in both the resistant lines compared to 3 in the other lines.

We have also studied MeCCNU-induced DNA damage in wild type LL and R4/1, using the occurrence of sister chromatid exchange (SCE) and micronucleus formation as end-points. The relationship between SCE occurrence and drug dose was similar for both lines; however micronucleus formation at a given dose was substantially less in R4/1, consistent with its resistance to the drug.

The micronucleus assay is a measure of chromosome fragmentation and loss, and suggests that MeCCNU is capable of inducing chromosomal changes leading to altered cell karyotypes. Studies are continuing to further elucidate the mechanism of appearance of these genetically distinct lines, and also to investigate the specificity of chromosomal markers in resistant tumour lines.

**CYTOTOXICITY AND ANTITUMOUR ACTIVITY OF N-HYDROXYMETHYLFORMAMIDE, A PUTATIVE METABOLITE OF N-METHYLFORMAMIDE.** P. G. Cooksey, E. N. Gate, A. Gescher, J. A. Hickman, S. P. Langdon & A. E. Wilson*, *C.R.C. Experimental Cancer Chemotherapy Group, University of Aston, Birmingham B4 7ET and *Withington Hospital, Manchester M20 8LR*

N-Methylformamide (NMF) is active against certain murine and human xenograft tumours in mice and is currently undergoing Phase 1 trials in man. There is evidence that is activity depends upon metabolic activation *in vivo* (Gescher *et al.*, 1982, *Br. J. Cancer*, **45,** 843). N-Hydroxymethylformamide (HMF), which appears to be formed during oxidative metabolism of NMF *in vivo* (Ross *et al.*, 1982, *Br. J. Cancer*, **44,** 278), is a possible candidate for the active species. HMF (30 mM) gave approximately a 4 log cell kill of TLX5 lymphoma cells incubated for 2 h at 37°C whereas 500mM NMF was non-toxic. HMF (2·5 mM) inhibited the incorporation of radiolabelled formate, leucine, uridine and thymidine into cellular macromolecules of TLX5 cells under these incubation conditions. The inhibition of uridine incorporation into TLX5 cells was abolished by preincubation of the cells with 2·5mM semicarbazide. This suggests that the inhibition is caused by formaldehyde, a known cytotoxic agent, which is a decomposition product of HMF and found in small amounts ($<3\%$) in aqueous solutions of HMF. Similar results were obtained using human ovarian carcinoma cells *in vitro.* HMF had no significant antitumour activity against murine tumours which are sensitive to NMF (M5076 sarcoma, TLX5 lymphoma) nor did it reduce hepatic glutathione, as did NMF. It is concluded that the formation of HMF may be a deactivation pathway of NMF metabolism, unlike the formation of an N-hydroxymethyl compound from another N-methyl-containing drug, hexamethylmelamine, in which case the N-hydroxymethyl compound appears to play a role in cytotoxicity (Rutty & Connors, 1977, *Biochem. Pharmacol.*, **26,** 2385).

**CHARACTERIZATION OF CHEMORESISTANCE OF HUMAN MALIGNANT MELANOMA TO DAUNORUBICIN, METHOTREXATE, VINDESINE AND DTIC.** J. M. Gaukroger*, N. G. L. Harding†, L. Wilson* & R. M. MacKie*, **Depts. of Dermatology and †Pathological Biochemistry, †University of Glasgow*

Following *in vitro* culture of melanoma cells in continuous contact with chemotherapeutic agents used for the treatment of melanoma and other malignant diseases, we have produced cell lines (murine and human) resistant to daunorubicin ($10^{-7}$M), methotrexate ($10^{-6}$M), DTIC ($10^{-3}$M) and vindesine ($5 \times 10^{-7}$M). The drug concentrations lethal to the wild type cells are respectively $10^{-10}$M, $10^{-10}$M, $10^{-5}$M and $10^{-10}$M.

We have developed *in vivo* 2 murine cell lines resistant to $10^{-3}$M DTIC, and we are currently developing vindesine resistant lines *in vivo*. Analysis of the genetic content of the DTIC resistant cell lines has not revealed any change in the base sequence of the cellular DNA and we are therefore studying amino-acid incorporation into cellular protein.

Vindesine is currently favoured for the treatment of malignant melanoma, and although we have been able to obtain the radiolabelled drug, it unfortunately appears to be subject to radiolysis. We have, however, commenced a study on the metabolism of vindesine by melanoma patients receiving this drug as a single agent. *In vitro* melanoma cells do not appear to metabolize this agent to any significant extent.

Studies with a tumour stem cell assay have just started with some success. Preliminary trials on continuous cell lines including a resistant human melanoma line, resulted in cell division and growth of clones visible to the naked eye after 3 weeks.

**FORMATION AND RETENTION OF METHOTREXATE POLYGLUTAMATES BY A HUMAN BREAST-CANCER CELL LINE IN THE PRESENCE AND ABSENCE OF INSULIN.** D. G. Kennedy*, R. Clarke*, H. W. van den Berg†, & R. F. Murphy*, **Dept Biochemistry and †Dept Therapeutics and Pharmacology, The Queen's University of Belfast*

The rate and extent of the formation of MTX poly-$\gamma$-glutamates has been studied in a human breast-cancer cell line (MDA.MB.436). Cells were exposed to medium containing $10^{-7}$M radiolabelled MTX for various times. Cell extracts were subjected to gel filtration on Bio-Gel P2 and radioactivity in each fraction determined. This has allowed quantitation of the relative amounts of MTX and its polyglutamate derivatives. It has been proposed that MTX polyglutamates may act as storage forms of the drug which could result in prolonged cytotoxicity. This possibility was investigated by determining the ability of the cell line to retain MTX and its polyglutamate derivatives for periods of up to 48 h after removal of MTX from the incubation medium. Our results show that the lower-mol. wt polyglutamates (<4 extra glutamic acid residues) rapidly efflux from the cell, while the higher-mol. wt species (>4 glutamic acid residues) are extensively retained, an efflux half-life of ~30 h. It has been shown that insulin may potentiate the cytotoxicity of MTX to breast-cancer cells *in vitro*. Our results indicate that in the MDA.MB.436 cell line total intracellular drug at steady state is unaffected by insulin ($10^{-6}$M). However insulin does increase by 26% in the contribution of the higher molecular weight polyglutamates to total intracellular drug. Our data therefore suggest that the ability of insulin to potentiate the cytotoxic effects of MTX may be related to the hormone's ability to modulate the synthesis of MTX polyglutamates.

**ISOLATION AND CHARACTERIZATION OF A SUBLINE OF CHO CELLS WITH INDUCED RESISTANCE TO ICRF 159.** S. J. Kenwrick & A. M. Creighton, *Cellular Pharmacology Laboratory, Imperial Cancer Research Fund, London WC2A 3PX*

A subline of CHO cells has been derived, with induced resistance to the antitumour drug ICRF 159. This subline (CHO/159–1) has a consistent resistance index of ~300 relative to the parent line in colony-forming assays, and has the same diploid chromosome number and growth rate. Since the presence of the detergent Tween 80 does not restore any sensitivity, it seems unlikely that the resistance is due to a membrane alteration affecting the uptake of the drug. No cross resistance has been demonstrated to a variety of cytotoxic drugs including alkylating agents and antimetabolites. A 2-fold increase in resistance has been observed to the anthracycline antibiotics, adriamycin and daunomycin similar to that found earlier with 1 out of 3 BHK lines with induced resistance to ICRF 159 (White & Creighton, 1976, *Br. J. Cancer*, **34**, 323). Although complete cross-resistance is shown to ICRF 202 (a more potent homologue of ICRF 159) CHO/159–1 is not significantly resistant to the Chinese drug AT-1727 (the *bis*-N-morpholinomethyl derivative of ICRF 154). The latter result is a little surprising since the morphological effects of

AT-1727 on cells closely resemble those produced by ICRF 159. Relevant markers have been introduced into both the parent and the drug-resistant sub-line and interspecific hybrids with mouse L/TK$^-$ cells have been shown to express a codominant resistant phenotype. Analysis of the DNA content by flow cytometry and of the chromosome number and type confirmed the hybrid nature of these cells. The codominant nature of the resistance makes it feasible to attempt its transfer into L/TK$^-$ cells with chromosome or DNA preparations.

**FLOW-CYTOFLUORIMETRIC DETERMINATION OF INTRACELLULAR LEVELS OF DAUNORUBICIN IN P388 CELL LINES SHOWING DIFFERENTIAL SENSITIVITY TO THE DRUG.**
A. T. McGown, T. H. Ward & B. W. Fox, *Experimental Chemotherapy, Paterson Laboratories, Christie Hospital and Holt Radium Institute, Manchester M20 9BX*

Resistance to the anthracycline antibiotics daunorubicin has been attributed to a net decrease in drug accumulation. The technique of flow-cytometry coupled with the intrinsic fluorescence of these agents allows the rapid examination of large, statistically valid, numbers of individual cells for drug content.

P388 mouse lymphoma cell lines showing differential sensitivity to daunorubicin have been developed. The resistant cell line can be seen to show a lower drug accumulation than the wild-type by both flow-cytometry and by extraction of drug from the cells. The effect of pH, temperature, drug concentration, and metabolic inhibitors on drug uptake have been investigated.

**FLOW-CYTOFLUORIMETRIC STUDIES WITH A FLUORESCENT DERIVATIVE OF METHOTREXATE ON SENSITIVE AND RESISTANT CELL LINES.**
D. G. Poppitt, A. T. McGown, & B. W. Fox, *Paterson Laboratories, Christie Hospital and Holt Radium Institute, Manchester M20 9BX*

L1210 mouse leukaemia cell lines showing differential sensitivity to the antimetabolite MTX, have been studied using techniques of cell culture and flow cytofluorimetry. A fluorescent derivative of MTX has been prepared and comparative studies of enzyme inhibition and cell survival with MTX suggest similar modes of action. The resistant cell line has been shown previously to owe its resistance to an increased production of the enzyme, dihydrofolate reductase. Flow cytofluorimetric studies after treatment with fluorescent methotrexate can detect cells with increased dihydrofolate reductase. Furthermore, in a mixed cell population, it is possible using a Fluorescent Activated Cell Sorter (FACS), to recognize and separate the the 2 subpopulations.

**BIOCHEMICAL DISTURBANCES OBSERVED *IN VITRO* AND *IN VIVO* FOLLOWING INHIBITION OF THYMIDYLATE SYNTHETASE BY C 3717.**
A. L. Jackman, G. A. Taylor, A. H. Calvert, D. R. Newell & K. R. Harrap. *Dept. Biochem. Pharmacol., Inst., Cancer Res., Sutton, Surrey*

CB 3717 is a quinazoline-based folate analogue which is a potent inhibitor of thymidylate synthetase (TS) (Jones *et al.*, 1981, *Eur. J. Cancer*, **17**, 11. Inhibition of the *de novo* synthesis of thymidylate may be achieved by pyrimidine analogues (e.g. 5-fluorouracil) whose active metabolite, 5-fluorodeoxyuridylate, inhbiits TS, or indirectly by folate analogues (e.g. MTX), which inhibit dihydrofolate reductase. Both have additional loci of action: The former is incorporated into RNA and the latter inhibits *de novo* purine synthesis. There is strong evidence that in cultured cells the inhibition of TS is the sole cytotoxic locus of CB 3717. We also have evidence to suggest that the antitumour properties of the drug *in vivo* are also attributable to this same locus. Firstly, CB 3717 is not metabolized to a compound that might act at a different locus. Secondly, plasma levels of deoxyuridine in mice, following treatment with CB 3717, rise to a peak at 4 h. This observation correlates with the large increase in intracellular deoxyuridylate observed *in vitro*. Finally the antitumour activity of CB 3717 against the L1210 tumour is prevented by co-administration of thymidine. CB 3717 is currently undergoing clinical evaluation which will allow the therapeutic value of the specific inhibition of TS to be evaluated in man for the first time.

### ALGINATE: A NEW REVERSIBLE GELLING MEDIUM FOR INVESTIGATING CELL TRANSFORMATION.

J. P. Roscoe & A. M. Owsianka, *School of Pathology, Middlesex Hospital Medical School, London*

Previous investigations of the malignant transformation of rat brain cells by ethylnitrosourea (ENU) have shown that tumorigenic cells form colonies in agar. Furthermore, pre-neoplastic cells which do not form colonies nevertheless survive, but in an essentially non-dividing state, for much longer (up to 10 weeks) than control cells (Roscoe & Winslow, 1981, *Br. J. Cancer*, **41**, 992). Investigation of this phenomenon has been hampered by the technical difficulties of long term maintenance and recovery of cells suspended in methocel or agar. We have developed alginate as an alternative suspending medium from which viable cells can be recovered by disrupting the cation dependent linkages with a chelating agent. A base layer of 0·6% agar is allowed to set and the appropriate concentration of CaCl solution placed on top. Cells suspended in 1% sodium alginate (Manucol FH, Alginate Industries Ltd, London) are immediately overlaid, forming a gel as the Ca ions react with the alginate. Cells can be released by addition of EDTA and collected by centrifugation. The results using this medium show that (1) tumorigenic and non-tumorigenic cells can be distinguished by colony-forming ability in alginate (2) the difference in viability of pre-neoplastic and control cells in agar can be reproduced, with the advantage that the recovery over several weeks can be quantitated. The method is therefore useful as a test for transformation and for investigating anchorage dependence and similar phenomena.

### HETEROGENEITY IN THE RESPONSE OF LEWIS LUNG TUMOURS (LL) TO MeCCNU.

T. C. Stephens, K. Adams & J. H. Peacock, *Institute of Cancer Research, Sutton, Surrey*

Using an excision cell-survival assay, we have demonstrated that most cells in wild-type LL tumours are exquisitely sensitive to the chemotherapeutic drug MeCCNU (survival curve $D_{10} = 1{\cdot}8$ mg/kg). Cell-survival data predict that 0·2 g tumours should be cured by a single dose of 15 mg/kg (about 1/3 $LD_{10}$), but in practice only about 30% cures can be achieved at 40 mg/kg. Model calculations show that this discrepancy can be explained if the tumours contain a subpopulation ($\sim 10^{-6}$) of cells which are *ca* $5\text{–}10 \times 10^{-6}$ more resistant to$^5$the drug, and the existence of a biphasic growth delay curve supports the model. However, the model also predicts that *all* tumours regrowing after high MeCCNU doses should be resistant to the drug, but this was *not* found. Of 4 tumour lines which regrew after 40 mg/kg, only 1 was more resistant than wild-type LL, using both cell survival and regrowth delay endpoints. In a repeat experiment, using regrowth delay only, 2 lines were resistant, 4 were of similar sensitivity to wild-type LL and 2 were more sensitive. For comparison, 6 clonal lines, derived from previously untreated wild-type LL, showed the same sensitivity to MeCCNU as the wild-type tumour. Therefore, a more complex model, possibly involving sanctuary sites in which inherently sensitive cells are protected from drug exposure, or perhaps drug-induced fluctuations in the sensitivity of cells to MeCCNU, is necessary. It may be relevant that MeCCNU has a substantial mutagenic effect in mammalian cells, and we have shown that it can induce chromosome fragmentation (these Abstracts). Experiments involving fractionated MeCCNU treatments are underway, in order to characterize more precisely the mechanisms underlying resistance to this chemotherapeutic agent.

### MODIFICATIONS OF CLONOGENIC ASSAY PROCEDURES RESULTING IN ENHANCED PLATING EFFICIENCIES FOR HUMAN TUMOUR-CELL LINES.

R. D. H. Whelan & B. T. Hill, *Laboratory of Cellular Chemotherapy, Imperial Cancer Research Fund, London WC2A 3PX*

In an attempt to enhance the colony-forming efficiency (CFE) of certain established human tumour-cell lines, we have compared the published assay methods of Chu & Fischer

(1968, *Biochem. Pharmacol.*, **17,** 753), Courtenay & Mills (1978, *Br. J. Cancer*, **38,** 77) and Hamburger & Salmon (1977, *Science*, **197,** 461) and made certain modifications. The lines tested included 2 derived from colon, COLO 205 and LoVo, 2 from prostate, DU145 and PC3mA2 and 2 neuroblastomas, CHP100 and LAN-1.

Using the first soft-agar procedure, a reduction of the agar or agarose concentration from 0·3% to 0·18% increased the CFE (2–15-fold with all the lines, except the LoVo cells). The Courtenay assay proved most valuable, improving for example, the CFE of COLO 205 cells from 2% to 40% and PC3mA2 cells from 1·5% to 37% in 0·3% agar. CFE may be further increased (up to 2-fold) by a reduction of the agar concentration. The quality of colonies produced was also superior and they could be scored in 6–12 days instead of 2–4 weeks. The Hamburger & Salmon assay failed to support reproducible colony growth. The addition of EGF did not enhance the CFE in any of these cell lines. However, the substitution of Ham's F12 medium, 10% foetal calf serum and RBC from August rats recommended by Courtenay, resulted in satisfactory CFEs, comparable to those obtained with the Courtenay assay. Use of the low $O_2$ gassing mixture had minimal effects.

Drug sensitivities of the cell lines tested were not altered by enhancing CFE by these modifications. We recommend wider use of the Courtenay procedure, together with reduced agar/agarose concentrations.

### DEMONSTRATION OF PATCHES OF DIFFERING GENOTYPE IN TISSUE SECTIONS OF GENETICALLY MOSAIC MICE.

B. A. J. Ponder*, M. Wilkinson*, D. Robertson*, P. E. Monaghan† & M. Wood‡. **Institute of Cancer Research; †Ludwig Institute for Cancer Research, Clifton Avenue, Sutton, Surrey; and ‡MRC Laboratory Animal Centre, Carshalton, Surrey*

Genetically mosaic mice are made by aggregation of early embryos of mice of different inbred strains. The aggregated embryos are transferred to the uterus of a pseudopregnant foster mother and develop in the normal way. The tissues of the resulting chimeras are a mosaic of patches of cells derived from each of the 2 embryos. These mice are potentially useful for studies of cell lineages in development, of organogenesis, tissue organization, and of the cellular populations involved in pre-neoplastic and early neoplastic lesions. A major limitation on their use has been the lack of a general method to distinguish the cells of the 2 component genotypes in tissue sections.

We describe the successful development of such a method using immunohistochemical techniques with (i) monoclonal antibodies to H2 antigens in $H2^a \leftrightarrow H2^b$ chimeras and (ii) a newly discovered polymorphism for binding of *Dolichos biflorus* lectin to gut epithelium and vascular endothelium of different mouse strains. Preliminary results from analysis of 11 chimeras suggest that (i) patches in the epithelium examined are mostly rather large (of the order of several hundred cells), but that patches in vascular endothelium are probably smaller and (ii) the epithelium of an intestinal crypt is always composed entirely of cells of one genotype, suggesting origin from a single precursor cell.

### GROWTH OF HUMAN TUMOUR CELL COLONIES FROM XENOGRAFTS USING 3 SOFT-AGAR TECHNIQUES.

R. Ranjini Rao, *Dept Radiotherapy Research, Institute of Cancer Research, Sutton, Surrey*

Three methods are currently in use for *in vitro* clonogenicity assay of human tumour cells: Method A (Courtenay, 1976, *Br. J. Cancer*, **34,** 39); Method B (Hamburger & Salmon, 1977, *Science*, **197,** 461) and Method C (Courtenay & Mills, 1978, *Br. J. Cancer*, **37,** 261). The plating efficiency of tumour cells from human melanoma biopsies and from xenografts was much higher with Method C than Method B (Tveit *et al.*, 1981, *Br. J. Cancer*, **44,** 530).

The objective of the present study was to compare the clonogenicity of human tumour cells from xenografts of 5 different tumours using the same 3 methods. The techniques for preparing single cell suspensions and for plating the cells in the agar cultures were as reported for these methods, except that conditioned media and 2-mercaptoethanol were omitted from Method B. The 5 human xenografts used were HX117 (melanoma), HX70 (adenocarcinoma—lung), HX113 (adenocarcinoma—ovary), HX69 and HX123 (small-cell carcinoma). Colonies of more than 50 cells were counted, usually after 14–21 days of incubation. PE of 4–37% were obtained with HX117, HX70 and HX123 in Method C, and of 2–17% with Method A. No colonies were detected with Method B, though some clumps were noted. HX113 did not form colonies in soft agar with any of the 3 methods. A few colonies (PE 0·007%) were obtained from HX69 cells using Method C after 6 week incubation.

These results suggest that Methods A and C which incorporate rat (August) red blood cells and hypoxic gas phase (5% $CO_2$/5% $O_2$/90% $N_2$) yield much higher PEs than Method B, not only with human melanoma xenografts but with other types of human tumour xenografts as well.

### THE USE OF VINCRISTINE TO MEASURE THE CELL PRODUCTION RATE IN A MOUSE C3H MAMMARY CARCINOMA FOLLOWING IRRADIATION.

B. Jones & R. S. Camplejohn, *Richard Dimbleby Department of Cancer Research, St Thomas' Hospital Medical School, London SE1 7EH*

The stathmokinetic method has proved useful in the investigation of cell populations which have fluctuations in their cell kinetics (e.g. Wright *et al.*, 1980, p. 102 in *Cell Proliferation in the Gastro Intestinal Tract*). In the current study, the stathmokinetic effect of Vincristine was used to determine cell production rates in a transplantable mouse C3H mammary carcinomas after irradiation (20 Gy). Remarkable constant values of cell production were found during a 7-day period in which there was no growth.

The apparent discrepancy of a constant flow of cells into mitosis in the absence of tumour growth, must be explained by an increase in cell loss following irradiation. Histological methods were used to monitor the time course of such changes. A 7-fold increase in the percentage of pyknotic cells was observed by Day 2 following irradiation and normal values were again attained by Day 7. These changes may reflect the processes of reoxygenation and repopulation known to influence the results of fractionated radiotherapy.

### QUANTITATION OF THERMOTOLERANCE CAPACITY OF AN EXPERIMENTAL RAT TUMOUR.

T. E. Wheldon, E. C. Hingston & J. L. Ledda, *M.R.C. Cyclotron Unit, Hammersmith Hospital, London W12*

The ability of tumours subjected to hyperthermia *in vivo* to acquire heat resistance (thermotolerance) was investigated using the rat fibrosarcoma SSB1a, serially transplanted in Wistar/CFHB rats in the dorsal site. Hyperthermia treatment was by waterbath heating of tumours, previously clamped to occlude blood flow, at 43·5°C for various times. Both single treatments (heating times 10–60 min) and split treatment (initial time 30 min, interval 24 or 48 h, variable second heating time 10–90 min) were given and tumour response assessed as growth delay. Single treatments yielded a linear dose–response curve, whilst split treatments (at both 24 and 48 h intervals) yielded less uniform, though approximately linear, dose-response curves of considerably shallower slope. Comparison of the slopes of these dose–response curves gave a "Thermotolerance ratio" (TTR) of 4·09 at 24 h and 3·45 at 48 h, indicating a large capacity for thermotolerance. Monte Carlo simulation studies suggest that the distribution of tumour sensitivities to a second treatment is much broader than to a first treatment. Thermotolerance is capable of providing substantial but variable protection of tumours against subsequent hyperthermia, and is influenced by factors other than those which determine response to a first heat treatment.

### RELATIONSHIPS BETWEEN RADIATION CELL-SURVIVAL-CURVE PARAMETERS.

J. Kirk & G. F. Brunton, *Glasgow Radiobiology Group, Glasgow Institute of Radiotherapeutics and Oncology, Belvidere Hospital, London Road, Glasgow G31 4PG and West of Scotland Health Boards, Department of Clinical Physics and Bio-Engineering, Glasgow G4 9LF*

Assessment of cellular radiation damage is based on loss of reproductive integrity, quantified by the fraction of surviving clonogenic cells and depicted by cell-survival curves. Recent studies challenge the established view, held for more than 30 years, that the parameters of the expressions used to describe radiation cell-survival curves are independent and characteristic of a cell line. Investigations using CHO derived cell lines indicate on the contrary, that cell-survival parameters are related and that, within that constraint, cell lines can display different survival characteristics under different conditions and after trauma. That parametric relationship is not an artefact of formulation or data fitting, and has been identified for various melanoma cell lines in animal and man; *in vitro* and *in vivo*.

Further evidence (Sparrow *et al.*, 1967, *Radiat. Res.*, **32,** 915) supports the view that a simple relation exists between these cell survival curve parameters and chromosome volume or DNA content, strongly suggesting that radiation damage and repair may be linked through the chromosome volume.

Clinically, the implications of these relationships may be of considerable significance; offering the possibility of rapid assessment of cellular radiation characteristics for certain tumour classes from assays of the DNA content of cells taken from patients during biopsy, and offering the prospect for improved treatment of such radio-resistant tumours as melanoma.

### IDENTIFICATION OF 2 DIVIDING CELL POPULATIONS IN THE RAT JEJUNUM WHICH RESPOND DIFFERENTLY TO X-RAYS.

P. G. Barnes & N. A. Wright, *Histopathology Department, Royal Postgraduate Medical School, London W12 0HS*

A method has been devised for the investigation of any heterogeneity of cellular response to cytotoxic agents that may arise with respect to differentiation and position in the cell cycle.

From studies over a time course after exposure to a wide range of doses of X-rays, it has been found that:

1. The population of cells in the last transit cell cycle (a) undergoes death *via* liquefaction necrosis, (b) is the population from which survivors give rise to clonal repopulation after expsoure to high doses.
2. The population of cells in previous transit cell cycles (a) produces a large number of autophagosomes that contain nuclear material within 2 h of irradiation, (b) is unusually sensitive to a rapid, interphase cell death.

The biological origin of these differences is a matter of speculation. Identification of the point at which the differentiating progeny of the functional stem cells are no longer potential stem cells has previously been a matter of conjecture. These studies provide definitive evidence that cells lose their "stemness" at the last transit division. Two implications of this result are that in this renewal system (a) functional organization appears to differ from that in the haemopoietic system, where multipotential "stemness" is promptly lost on differentiation, (b) arguably, all dividing epithelial cells in tumours (of the intestine) may be clonogenic, as is the case in normal tissue.

### THE VALUE OF LACTATE DEHYDROGENASE AS A NON-SPECIFIC TUMOUR MARKER IN SEMINOMA OF THE TESTIS.

A. G. Robertson & G. Read, *Department of Radiotherapy, Christie Hospital, Manchester*

Serum $\alpha$-foetoprotein and $\beta$-HCG have been found to be excellent tumour markers in malignant teratoma of the testis, but are of little value in seminoma. Serum lactate dehydrogenase (LDH) has been found to be a non-specific tumour marker. Serum LDH levels were measured in 38 patients with seminoma of the testis treated between December 1980 and December 1981 at the Christie Hospital, Manchester. No patients with minimal or no para-aortic disease had raised levels of the marker. Ten out of 11 patients with more advanced disease had high levels. Serum levels were measured during treatment with radiotherapy and chemotherapy. Of the 10 patients with high levels, 7 returned to normal before the completion of treatment. Subsequent evaluation with computer assisted tomography confirmed complete resolution of the disease. One patient's level fell, but not to normal, and at his first follow-up visit was found to have further metastatic disease. One patient with rising levels died of uncontrolled disease and the third had residual disease. In 1 patient, on follow-up, levels rose before clinical recurrence became apparent. It is suggested that LDH levels may aid assessment of the extent of disease in seminoma and its response to treatment.

**THE TECHNIQUE AND POTENTIAL APPLICATION OF PROGESTERONE RECEPTOR MEASUREMENT BY ISOELECTRIC FOCUSING.** R. HARLAND, E. HAYWARD, D. M. BARNES, A. HOWELL, L. G. SKINNER & R. A. SELLWOOD, *Department of Surgery, Withington Hospital and Clinical Research Laboratories, Christie Hospital, Manchester*

Recently the induction of progesterone receptor (PR) synthesis in human breast cancer by tamoxifen has been described (Namer, *et al.*, 1980, *Cancer Res.*, **40,** 1750) and it has been suggested that this may give a specific prediction of hormone response. However, metastases may be inaccessible or too small for assay by standard techniques (Scatchard analysis). This reduces the value of any test which relies of PR measurement at the time of treatment.

A method has been developed which allows the use of small samples including needle biopsies, for PR estimation. After incubation of cytosol (minimum total volume 70 $\mu$l) with $^3$H-R5020 (a synthetic progestagen), hormone-labelled receptor was separated by iso-electric focusing (IEF). PR measured by IEF, though an underestimate, correlates with Scatchard analysis (rho = 0·89) with a specificity of 94% and a lower limit of sensitivity of 30 fmol/ml cytosol.

This method has allowed us to measure PR before and after starting tamoxifen in 14 patients, of whom 4 showed induction of PR. Progression of disease by 3 and 6 months respectively was seen in 0/4 and 0/3 patients in whom PR was induced, compared with 6/10 (NS) and 7/7 ($P = 0{\cdot}0167$) patients in whom no induction occurred. PR assay by IEF increases the availability of PR data in metastatic disease and may allow an early indication of hormone response.

**BACKSCATTERED ELECTRON IMAGING BY SEM OF NORMAL AND NEOPLASTIC URINARY BLADDER.** G. P. SMITH & A. E. WILLIAMS, *Teaching and Research Centre, University of Edinburgh, Western General Hospital, Edinburgh*

The study of urinary bladder cancer by scanning electron microscopy (SEM) has concentrated on the changes in luminal surface structure and organization during neoplasia (Hodges, 1979, in *Biomedical Research Applications of Scanning Electron Microscopy Vol* **1,** p. 307). However, SEM backscattered electron imaging (BSE) of tissues stained with heavy elements such as silver, allows visualization of subsurface structures (Becker & Sorgard, 1979, *SEM*/**II,** 825). This method was used to examine normal and carcinogen treated rat bladders, and biopsies from patients under investigation for bladder cancer. Results show a predominance of binucleate superficial cells in the bladders of normal rats, and an organizational relationship between the superficial layer of cells and the underlying intermediary cell nuclei suggestive of the proliferative unit organization seen in epidermis (Potten, 1974, *Cell Tissue Kinet*, **7,** 77). In rat bladders treated with methylnitrosurea, changes were observed in intracellular organization before urothelial changes could be observed histologically or by SEM examination of surface topology; superficial cells became predominantly uninuclear, and the relationship between superficial and intermediary cells was lost. Areas where these changes have occurred can be readily seen at low magnification. Preliminary observations of human biopsy material suggest that similar organizational and neoplastic changes occur. The results suggest that the use of BSE imaging may provide a useful tool in the early diagnosis of bladder cancer.

**PROPERTIES OF DIHYDROFOLATE REDUCTASE FROM HUMAN TUMOURS.** J. A. BLAIR, A. E. PHEASANT, A. M. SALEH, A. SAHOTA, S. B. WHITBURN, G. D. OATES & R. N. ALLAN, *Department of Chemistry, University of Aston in Birmingham, Birmingham B4 7ET and The General Hospital, Steelhouse Lane, Birmingham 4*

Dihydrofolate reductase (DHFR) plays an important role in cell proliferation. Its inhibition arrests the cell cycle at the $G_1$-S transition. 10-Formylfolate and 10-formylfolatetetraglutamate are formed by oxidation of the tetrahydro forms found in mammalian cells. Bovine liver DHFR does not reduce 10-formylfolate and is inhibited by 10-formylfolate and 10-formylfolatetetraglutamate ($K_i = 2 \times 10^{-8}$M and $1{\cdot}3 \times 10^{-8}$M respectively). *Lactobacillus casei* DHFR reduces 10-formylfolate slowly and is weakly inhibited by it. In normal man 10-formylfolate is not reduced and inhibits the [5]reduction of folic acid by DHFR. DHFR from some human intestinal and breast tumours slowly reduces 10-formylfolate and is weakly inhibited by it. Thus DHFR from these tumours is similar to the *L. casei* enzyme, and different from the normal mammalian enzyme. $^{13}C$ NMR studies suggest that the reduced binding of 10-formylfolate to the *L. casei* DHFR is caused by lack of a nucleophilic group on the enzyme. It is suggested that an important event in some malignant transformations [5]is the synthesis via a mutated gene of a DHFR which no longer fully responds to a normal cell proliferation control mechanism.

**CORRELATION OF SIALYLTRANSFERASE AND SIALIC ACID AND THEIR COMPARISON WITH 6 OTHER BIOCHEMICAL MARKERS FOR MONITORING THE EXTENT OF DISEASE IN PATIENTS WITH BREAST CANCER.** B. J. McDermott*, C. C. Quinn*, B. M. Leamy†, L. Jeffrey‡ & R. F. Murphy*. **Dept Biochemistry, Queen's University Belfast; †Bon Secours Hospital, Cork and ‡Belvoir Park Hospital, Belfast*

For up to 24 months post-mastectomy the following serum markers were measured in 86 patients: carcinoembryonic antigen (CEA), C-reactive protein (CRP), α-acid glycoprotein (AGP), erythrocyte sedimentation rate (ESR), ¼-glutamyl transferase (GGT), alkaline phosphatase (AP), sialyl transferase (ST): sialic acid (SA) was determined retrospectively. Prior to therapy, there was a higher incidence of raised marker measurements in the sera of patients with metastatic cancer than in those who were apparently disease-free, except for ST. Only CEA and ST concentrations were raised in a significantly greater number of patients with minimal disease (lymph-node involvement or recurrence) than in the tumour-free group (CEA, $P \leqslant 0{\cdot}01$: ST, $P \leqslant 0{\cdot}005$). In those with minimal disease, 5/6 raised values were attributed to the lymph-node$^+$ group. A patient defined as tumour-free had the higher ST value. Lymph-node involvement was detected clinically 6 months later. Alterations in tumour burden were reflected in 50% of the changes in ST concentrations by comparison with a correlation of >75% for CEA, CRP and GGT measurements. ST values were raised up to 8 months before clinical detection of advancing disease in 9/17 patients. In 7 cases, ST concentrations reached a peak and then declined; notably in the evolution of metastases, values returned to normal before progression was overt. ST results have been substantiated by SA estimations in both vertical and horizontal studies. These markers appear to be particularly sensitive to distant spread of disease at an early stage.

**THE SURFACE PROTEINS OF HUMAN ASTROCYTOMAS IN CULTURE.** G. V. Sherbet*, M. Wildridge* & R. M. Kalbag†, **Cancer Research Unit, Royal Victoria Infirmary and †Department of Neurological Surgery, Newcastle General Hospital, Newcastle upon Tyne*

Cell cultures were initiated from human astrocytomas. Surface proteins of subconfluent cultures were labelled by lactoperoxidase-catalysed radio-iodination, and separated by electrophoresis on 6·0% cylindrical polyacrylamide gels. Normal glial and astrocytoma cells were found to have a common surface-protein pattern, but there were significant quantitative changes in the expression of certain protein components of the astrocytomas, as compared with normal glial cells. A component of mol. wt ~225K which appears, by criteria of electrophoretic behaviour and immunological reactivity with mono-specific anti-fibronectin antibodies, to be fibronectin, was reduced by between 15 and 70% in 6/8 astrocytomas. However, receptors for fibronectin could be detected on the surface of the cells. Another component(s) of average mol. wt of 148K appeared to be amplified by a factor of 1·6–3·5 in all astocrytomas. Surface components of mol. wt <75K also incorporated more radioiodine than did the corresponding external proteins of normal glial cells. It is suggested that some of these changes, especially in the 225K and 148K external proteins, may be associated with the malignant state.

### AN E.M. STUDY OF ALKALINE PHOSPHATASE IN A TRANSPLANTABLE OSTEOGENIC SARCOMA OF THE RAT.

P. M. INGLETON, P. V. GAITENS, L. A. COULTON* & R. G. G. RUSSELL*. *Departments of Zoology and *Human Metabolism and Clinical Biochemistry, The University, Sheffield S10 2TN*

Studies of a transplantable osteogenic sarcoma of the rat have shown that it lays down ground substance which can mineralize (Ingleton *et al.*, 1977, *Lab. Anim. Sci.*, **27**, 748); that areas, designated A–D, can be distinguished within the tumour mass on the basis of mitotic activity, hormone responsiveness and degree of necrosis and mineralization (Underwood *et al.*, 1979, *Eur. J. Cancer*, **15**, 1151); and that it is rich in bone-like alkaline phosphatase (AP) (Ingleton *et al.*, 1979, *Eur. J. Cancer*, **15**, 685). This report details ultrastructural investigations, using Gomori's technique, into the site of AP activity in the tumour, during growth after implantation. After only 15 days growth no distinct areas could be discerned in the tumour nodule, but AP activity was seen in cells throughout, particularly in Golgi membranes, as discrete punctate areas on plasma membranes and as tiny spots on matrix vesicle membranes. Active cells of areas A–D from tumours growing for 22–35 days had membrane-associated AP activity, even when dividing. Area C contained a high proportion of necrotic tissue and no AP activity could be detected in the cell debris. Organized collagen developed intercellularly during tumour growth and AP activity was detected in discrete bodies associated with collagen. Crystalline calcium spicules also developed, apparently from AP-containing bodies, whether on cell membranes or collagen bundles, notably in area D after 35 days growth. The close association of AP activity with tumour cells during early stages of tumour growth, and later appearance intercellularly and in venules, correlates with previous *in vivo* observations that serum AP concentrations only rise significantly after the tumour has been growing for at least 11 days.

### ISOFERRITIN STUDIES USING THE LEUCOCYTE-ADHERENCE INHIBITION ASSAY IN PATIENTS WITH MALIGNANT LYMPHOMA.

L. BRUCE, A. GRAIL & B. W. HANCOCK, *Department of Medicine, Royal Hallamshire Hospital, Sheffield*

We have shown (Hancock *et al.*, 1979, *Br. J. Haematol.*, **43**, 223) that ferritin from Hodgkin's disease involved spleen differs in its immunological reactions from ferritin from normal spleen.

LAI assays (Browne *et al.*, 1980, *Tumour Diagnos.*, **5**, 266) were performed on 17 patients with malignant lymphoma and 14 controls, comprising 5 patients with non-lymphomatous disease and 9 normal individuals. Ferritin was purified from autopsy spleens and resolved into a series of isoferritins of discrete pI by preparative isoelectric focusing. Normal spleen ferritin, lymphoma spleen ferritin and constituent acidic and basic isoferritins were tested in LAI assays.

A non-adherence index (NAI) was calculated for acidic and basic isoferritins in each patient and control. In the lymphoma group a NAI of 39·1 was obtained with the acidic isoferritins, compared to a NAI of −8·0 in the control group ($P<0{\cdot}01$). With the basic isoferritins, the lymphoma group gave a NAI of 17·5 and the control group −1·2 ($P<0{\cdot}2$).

Analytical isoelectric focusing of lymphoma-spleen ferritins revealed no extra acidic isoferritins in comparison with normal-spleen ferritin, but there were differences in basic components. The LAI results suggest a possible difference in the antigenicity of isoferritins associated with malignant lymphoma.

**INHIBITORY EFFECT OF THE LANDSCHÜTZ ASCITES CARCINOMA ON GRANULOMATOUS INFLAMMATORY RESPONSE INDUCED BY *CORYNEBACTERIUM PARVUM*** L. C. McIntosh, R. G. P. Pugh-Humphreys*, R. A. Fraser, A. W. Thomson & J. I. Milton, *Departments of Pathology and *Zoology, University of Aberdeen, Aberdeen AB9 2ZD, Scotland*

I.p. or i.v. administration of *Corynebacterium parvum* (*CP*) within MF1 mice resulted in a generalized inflammatory response, associated with marked hepatosplenomegaly and accompanied by a pronounced granulomatous response in the liver. Injection of the Landschütz ascites carcinoma (LAC) 24 h after the microorganism substantially reduced the intensity of the inflammatory response, and decreased both the frequency and size of the hepatic granulomas, as revealed by morphometric analysis of histological sections.

The difference in cellular composition of the granulomas between the experimental groups, as revealed by light microscopy, was further emphasized and characterized by ultrastructural studies. These revealed the predominance of macrophages within the granulomas in tumour-bearing mice, in contrast to the predominance of epithelioid cells in those lesions which developed in mice given *CP* alone.

Our experimental findings show that the inhibitory effect of the growing LAC on granuloma formation in response to *CP* cannot be ascribed to (a) sequestration of the microorganism within the growing tumour, nor (b) the action of a non-specific inflammatory stimulus nor (c) diversion and sequestration of mononuclear phagocytes in the growing tumour. The observed inhibition of liver granuloma formation is consistent with an effect mediated by a soluble, heat-stable tumour-associated factor(s).

**BLOOD GROUPS IN LUNG CANCER PATIENTS AND THE EFFECTS OF IMMUNOTHERAPY.** H. M. Anthony, *University Department of Immunology, Leeds General Infirmary*

In a trial started in 1975, lung cancer patients treated with Levamisole on an intermittent schedule starting before operation showed significantly greater post-operative mortality (Anthony *et al.*, 1979, *Thorax*, **34**,), particularly if they were Blood Group O or Rh(D) − ve. The trial patients have now been followed for at least 4 years, and deaths in persons of these blood groups were also significantly increased from 7 weeks after operation onwards if they received Levamisole.

The trial patients showed rather higher A/O and D+/D− relative incidences if they were node$^-$ than if they were node$^+$. This has been confirmed in 2 other series of lung-cancer patients, though the difference within each series did not reach significance. Preliminary results suggest higher A/O and D+/D− relative incidences in a series selected for longer survival, but no effect of blood group on survival of trial patients treated with placebo has been observed.

An interaction of blood group with the response to immunotherapy has also been described in AML by Harris *et al.* (2nd Meeting on the Immunotherapy of Cancer, N.I.H., in press), in which patients who were Blood Group A and/ or Rh(D) + ve survived longer if treated with immunotherapy. It seems that the interaction of genotype with the response to immunotherapy may be a more generalized effect, linked to differences in the mechanisms of resistance to tumour spread, though our data suggest that the mechanisms involved may not be identical in respect of ABO and Rh Groups.

***IN VITRO* LYMPHOCYTE RESPONSES AND PLASMA COPPER LEVELS IN PATIENTS WITH MALIGNANT LYMPHOMA.** M. D. Whitham & B. W. Hancock, *Department of Medicine, Royal Hallamshire Hospital, Sheffield*

In this study, *in vitro* lymphocyte response to PHA, T-cell population and plasma copper levels have been assessed in 70 patients with malignant lymphoma, as all of these indices have been shown to deviate from normal in these patients and elevated plasma copper has been associated with depressed *in vitro* lymphocyte responses (Hancock *et al.*, 1980, *Tumor Diagnosis*, **3**, 140).

39 patients with Hodgkin's disease (HD) and 31 with non-Hodgkin's lymphoma (NHL)

have been tested together with groups of age/sex-matched controls. Taken as groups, significant differences were seen from control responses in HD for PHA response in autologous plasma ($P<0{\cdot}001$) and AB serum ($P<0{\cdot}005$), T-cell population ($P<0{\cdot}025$) and plasma copper ($P<0{\cdot}05$). In the NHL group significant differences were seen in PHA response in autologous plasma ($P<0{\cdot}001$) but not in AB serum, in T-cell population ($P<0{\cdot}005$) but not in plasma copper. Taking individual responses, in HD generally the number of abnormal values increase with disease stage for PHA response and plasma copper but not T-cells. In NHL the relationship between stage and abnormal values is not as clear; only plasma copper levels show any association. No association was found in either group between high plasma copper and depressed lymphocyte responses.

We have observed abnormal PHA responses and T-cell counts in both HD and NHL and abnormal copper levels in HD, and are continuing the study to see whether these responses correlate with response to treatment.

### INCIDENCE AND TYPE OF TUMOURS INDUCED BY ORAL DMBA IN NK-DEFICIENT C57B1 BG/BG MICE, +/BG LITTERMATES AND H-2 CONGENIC STRAINS OF B10 OF VARYING NK ACTIVITY.

A. J. Cochran, S. Argov†, K. Kärre†, G. O. Klein* & G. Klein*, **Pathology and Surgery, University of California, Los Angeles and †Department of Tumor Biology, Karolinska Institutet, Stockholm*

To study the effect of the beige mutation on chemical carcinogenesis, 64 C57BL bg/bg mice and 83 ± /bg littermate controls received 5 weekly intragastric doses of DMBA. We examined the incidence and histological types of tumours that developed. By 165 days after first DMBA contact 18% of ±/bg, and 31% of *bg/bg* mice developed tumours. Beige mice had a higher incidence of epithelial and non-epithelial tumuors in cutaneous or subcutaneous sites. The incidence of lymphomas was similar in the groups but they appeared earlier in the beige. After 500 days, 33 ± /*bg* and 27 *bg/bg* mice were still alive, 60/83 ± /bg animals (72%) and 47/64 *bg/bg* (74%) having developed tumours. The beige group showed a higher incidence of non-thymic lymphomas, but the incidence of thymic lymphomas, cutaneous epithelial tumours and bile-duct adenomas was similar in the 2 groups.

In a continuing study of NK, intact B10 (27), B10A (25), B10.BR (39), B10G (48) mice and developed more frequently in B10S congenic animals (30), squamous carcinomas have developed more frequently in B10S animals (B10S 8/30–27%) than in the rest (11/139–8%, $P<0{\cdot}05$). At present it looks as though both thymic and extrathymic lymphomas may also be of increased frequency in the B10S, NK-deficient animals.

The role of DMBA as a suppressor of NK activity has been confirmed.

### *IN VIVO* AND *IN VITRO* CHARACTERIZATION OF A TRANSPLANTABLE, SPONTANEOUS MURINE ASTROCYTOMA.

G. J. Pilkington*, J. L. Darling†, P. L. Lantos* & D. G. T. Thomas†, **Department of Neuropathology, Institute of Psychiatry and †Gough Cooper Department of Neurological Surgery, Institute of Neurology, London*

Spontaneous brain tumours of mice are rare, but Fraser (1971, *J. Pathol.*, **103**, 266) found gliomas in 1·5% of mice from the inbred VM strain. Homogenates of tumour-bearing brain when injected intracerebrally into syngeneic mice produced anaplastic astrocytomas. The present paper describes the fine structure and immunohistochemistry of these transplanted tumours.

Three cell lines derived from VM tumours (Serano *et al.*, 1980, *Acta Neuropathol.*, **51**, 53) have also been maintained *in vitro* and examined by fluorescence and electron microscopy in order to establish the glial nature of the cells. Immunofluorescence studies using the astrocyte specific markers (glial fibrillary acidic protein and glutamine synthetase) and large extracellular transformation-sensitive protein were carried out.

TEM of cell pellets reveals different ratios of microtubules to 10 nm filaments in the 3 cell lines; this indicates the degree of cellular differentiation. The effects of dbcAMP on the surface activity and organelle distribution have also been assessed. Multicellular spheroids have been induced and examined by scanning and transmission EM in order to elucidate the extent of adhesion between cells.

Tumours are now being induced in VM mice by intracerebral injection of cells from the line with the most obvious astrocytic features.

**LIPID CLEARANCE IN A COLONIC TUMOUR MODEL IN RATS.** R. WINDLE & P. R. F. BELL, *Department of Surgery, University of Leicester, Royal Infirmary, Leicester*

Marked derangement of fat metabolism occurs in cancer. Body fat is lost and serum triglyceride (TG) levels may be elevated (Dilman *et al.*, 1981, *Br. J. Cancer*, **43,** 637). Although adipose tissue lipolysis is increased in experimental cancer (Kralovic *et al.*, 1977, *Eur. J. Cancer*, **13,** 1071) an alternative explanation may be that there is a reduction in turnover of circulating TG. This may be measured by the clearance of an exogenous lipid emulsion load (Rossner *et al.*, 1974, *Eur. J. Clin. Invest.*, **4,** 109).

In order to test this hypothesis lipid clearance has been measured with TG levels during the induction of a colorectal cancer model in rats. Tumours were induced by 15 weekly injections of 15 mg/kg dimethylhydrazine (DMH). Serum TG and the rate of clearance of i.v. lipid emulsion (0·5 ml/kg of 20% intralipid) were measured before DMH and 8 weeks thereafter in 6 groups of 12 fasted rats. Groups of age-matched rats not given DMH acted as controls.

Tumours appeared at 32 weeks in the DMH groups. TG levels were reduced by 18% at 16 and 24 weeks and then became raised by 33% over pretreatment levels at 48 weeks, by which time the animals had lost 15–20% of their body weight. Lipid clearance was significantly increased at 16 and 24 weeks and then reduced by half at 48 weeks. Administration of heparin at 48 weeks increased lipid clearance and reduced TG levels. There was no change with time in TG levels or in lipid clearance rate in the control groups. In conclusion, TG levels are related to the rate of lipid turnover. The early increase of clearance may have been caused by DMH. A large tumour load was associated with raised TG levels and a reduction in turnover.

**THE EFFECT OF A SUB-CUTANEOUS GROWING TUMOUR ON THE ACTIVITY OF HOST TISSUE MITOCHONDRIA.** J. CUMMINGS & K. C. CALMAN, *Department of Oncology, University of Glasgow*

Mitochondrial fractions were prepared from the liver, kidney and skeletal muscle of rats 1, 6, 12, 18 and 26 days after an s.c. injection of either a suspension of Walker carcinosarcoma cells ($10^5$) or sterile saline, and immediately assessed for structural and functional integrity. Using a polarographic technique to measure respiratory capacity, it was found that Day 1 mitochondria from both controls and tumour-bearing animals showed signs of damage and impaired respiratory function. This effect was believed to be caused by the presence of anaesthetic molecules in the mitochondrial membranes. On Day 6 all mitochondria were respiring properly; no evidence of membrane disrupttion was apparent. From Day 12 onwards, however, by which time tumours had become palpable, mitochondria from rats injected with the tumour cells would not consume $O_2$ unless magnesium ions ($MgCl_2$) were added to incubation media. Over this period of time, control animal mitochondria were still respiring normally. Both types of mitochondria were structurally intact. Kidney mitochondria required by far the greatest amount of magnesium for normal activity; in some cases 10 mM $MgCl_2$. The liver's requirement never exceeded 3 mM $MgCl_2$. The quantity of magnesium ions necessary to restore normal mitochondrial function increased with tumour size. These results suggest that host tissue mitochondria of the tumour-bearing animals became increasingly more deficient in magnesium ions with tumour growth, magnesium ions are essential for normal respiratory activity and magnesium repletion *in vitro*, at least, restores normal mitochondrial function.

**STUDIES ON THE COMPOSITION OF HUMAN BREAST CYST FLUID.** P. L. Yap, W. R. Miller, M. M. Roberts, B. Freedman, C. L. Mirtle, A. D. Pryde & D. B. L. McClelland, *Blood Transfusion Service, University Department of Clinical Surgery and University Department of Therapeutics and Clinical Pharmacology, Royal Infirmary, Edinburgh EH3 9HB*

An increased risk of breast carcinoma exists in women with gross cystic disease of the breast (GCD), suggesting that the 2 diseases share a common aetiology. We have therefore studied cyst fluid in an effort to discover its origin.

Wide variations in the concentration of IgA, IgG, lactoferrin, lysozyme, albumin and dehydroepiandrosterone sulphate (DHAS) were found in 96 cyst fluids obtained from 75 patients with GCD. Sedimentation-coefficient determinations performed on the 19 cyst fluids with the highest IgA concentrations indicated that 10 contained mainly 11S (secretory) IgA and resembled external mammary secretions such as colostrum and milk. These cysts also contained high concentrations of DHAS ($80{\cdot}9 \pm 28{\cdot}9$ mg/l; mean $\pm$ s.e.) but low concentrations of IgG and albumin. The remaining 9 cyst fluids resembled serum, with the IgA mainly of the 7S (serum) type, and these cysts contained low concentrations of DHAS ($5{\cdot}9 \pm 1{\cdot}9$ mg/l) but high concentrations of IgG and albumin. Other cyst fluids not analysed by ultracentrifugation contained low concentrations of all the proteins measured. No relationship was observed between the concentrations of the substances studied, and clinical factors.

The differing composition of cyst fluid suggests that components of the fluid may be derived from either serum or breast sources. There may therefore be more than one mode of cyst formation.

**CHARACTERIZATION OF HAMSTER NK CELLS.** D. M. Teale, R. C. Rees, A. Clark & C. W. Potter, *Department of Virology, University of Sheffield Medical School, Sheffield S10 2RX*

The importance of NK cells in tumour immunology has previously been reviewed (Herberman & Holden, 1978, *Adv. Cancer Res.*, **27,** 305), and their association with large granular lymphocytes has been described in the human, rat and mouse (Timonen *et al.*, 1981, *J. Exp. Med.*, **153,** 569). In this report we describe the existence of a cytotoxic cell population, in hamster peripheral blood, capable of lysing NK-susceptible target cells in a 4h Cr-release assay. These cells proved to be non-adherent to nylon–wool, non-phagocytic and capable of lysing xenogeneic targets. When separated on Percoll Discontinuous Density Gradients cytotoxicity was associated with fractions enriched for large, sometimes granular, lymphocytes. In addition, cells with the morphology of large lymphocytes were shown to bind to NK-sensitive target cells. NK activity was only demonstrable in spleen and peripheral blood, with low levels in peritoneal-exudate cells, and activity was shown not to be age-restricted.


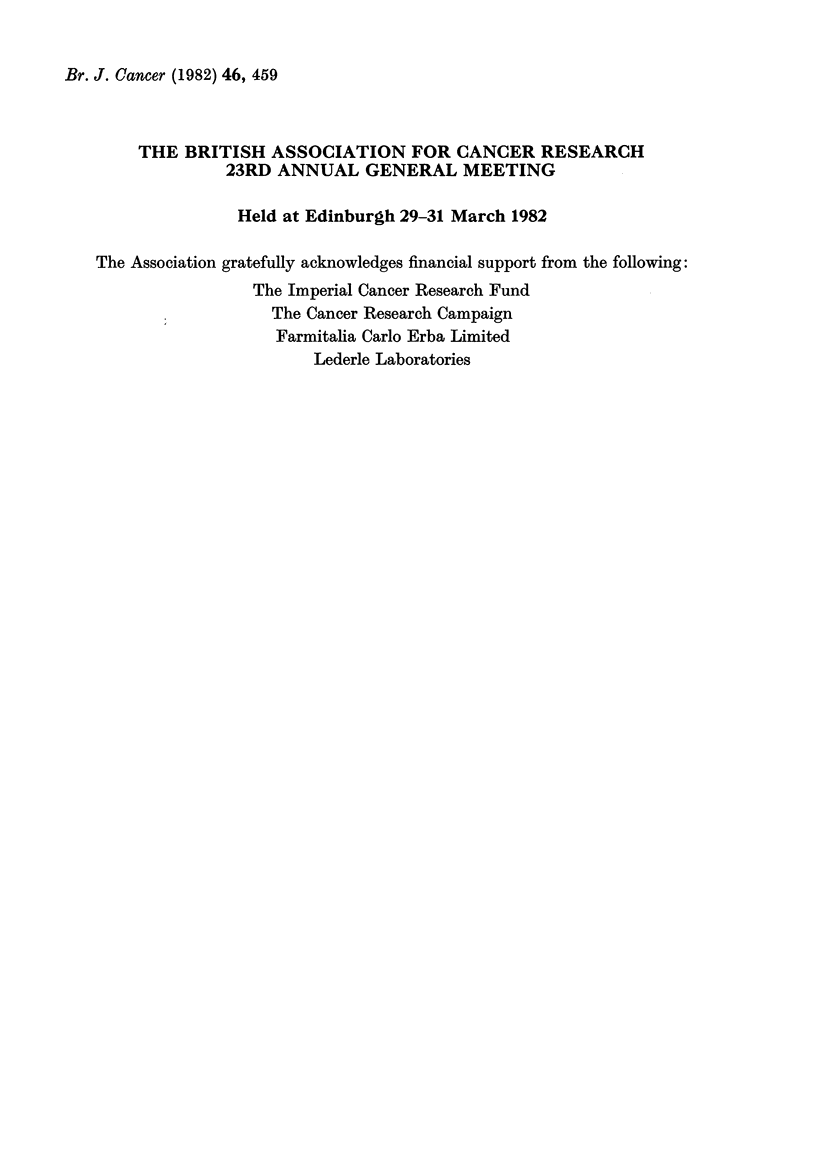

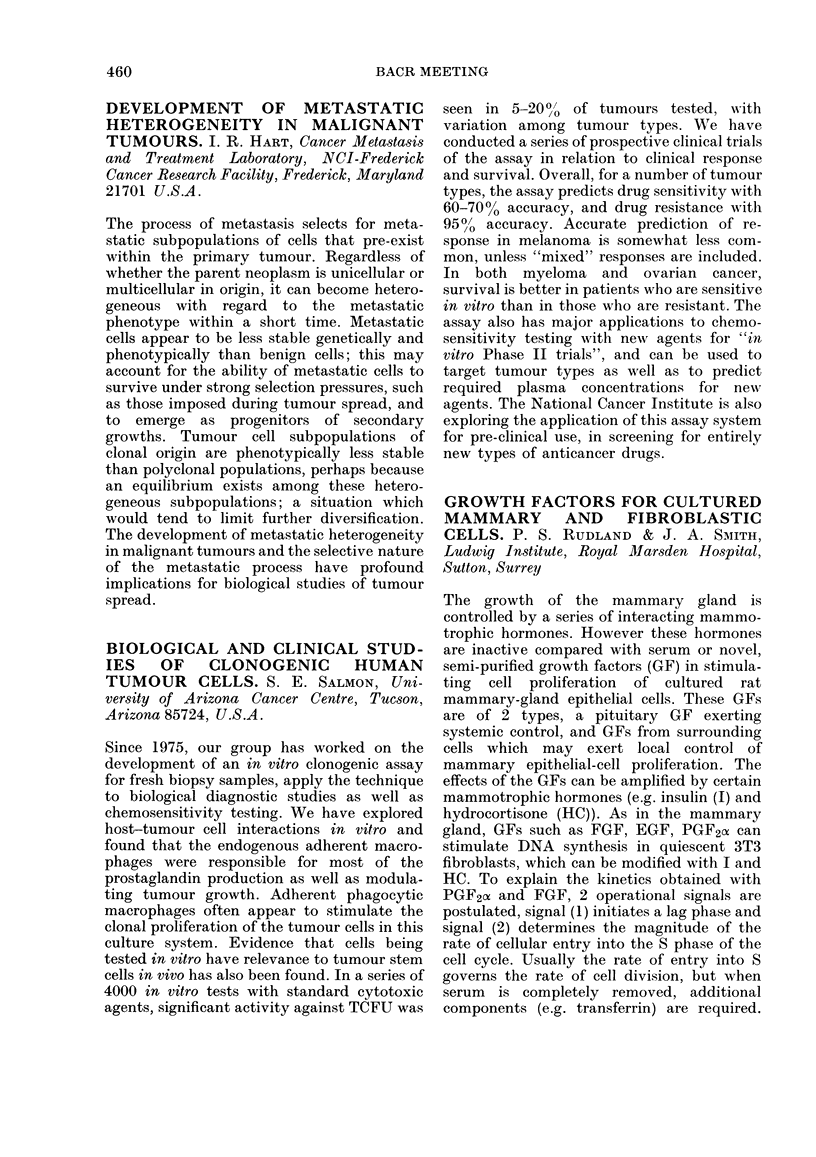

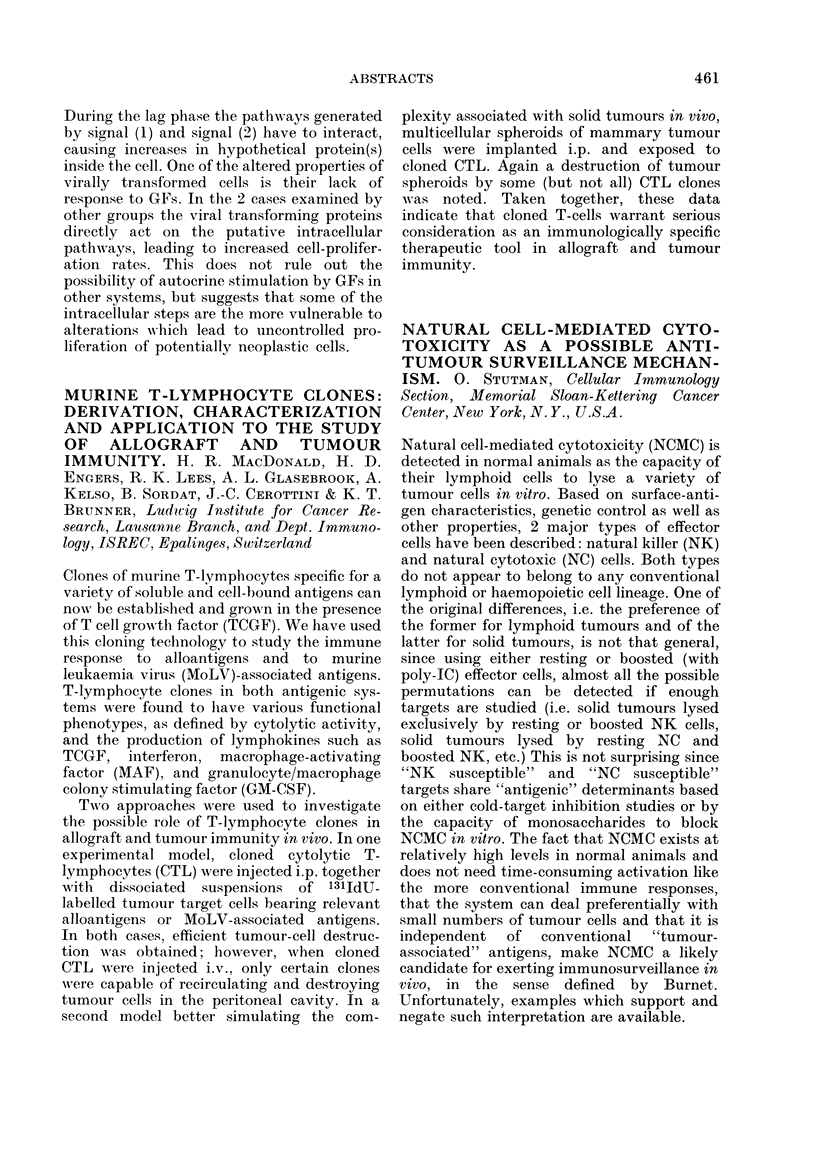

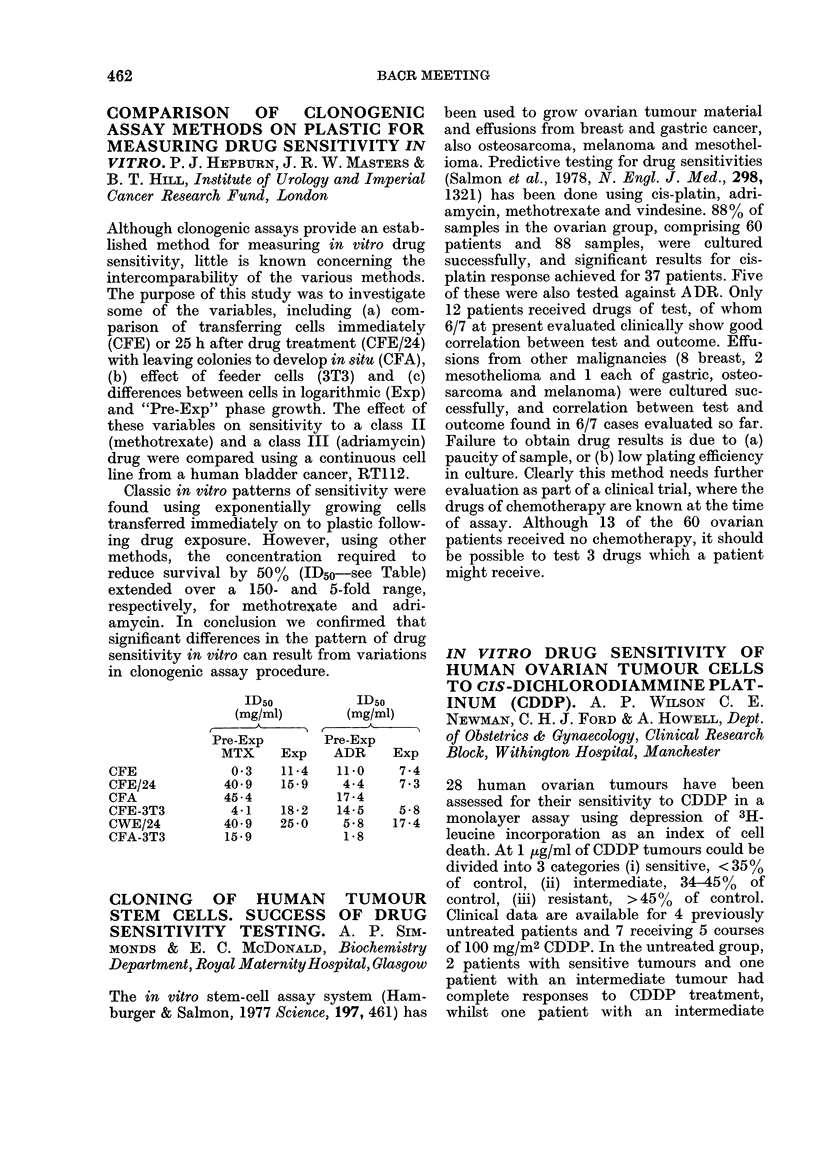

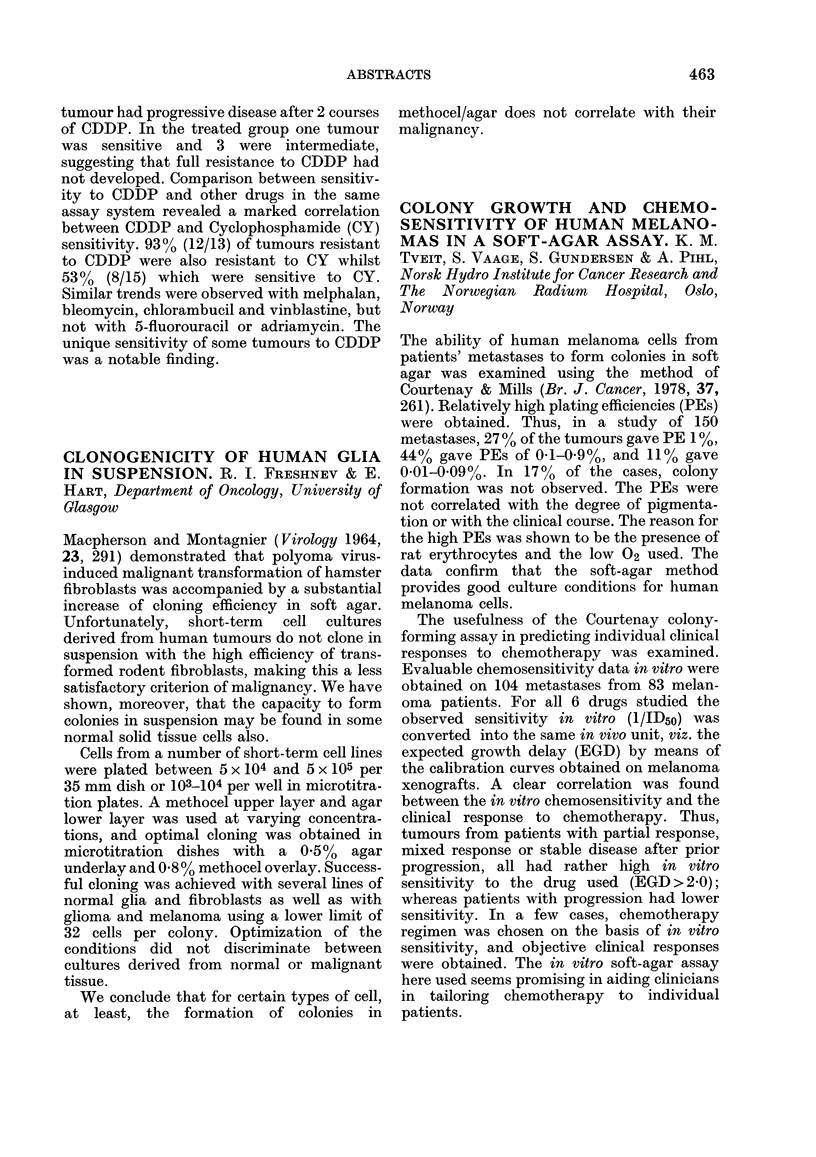

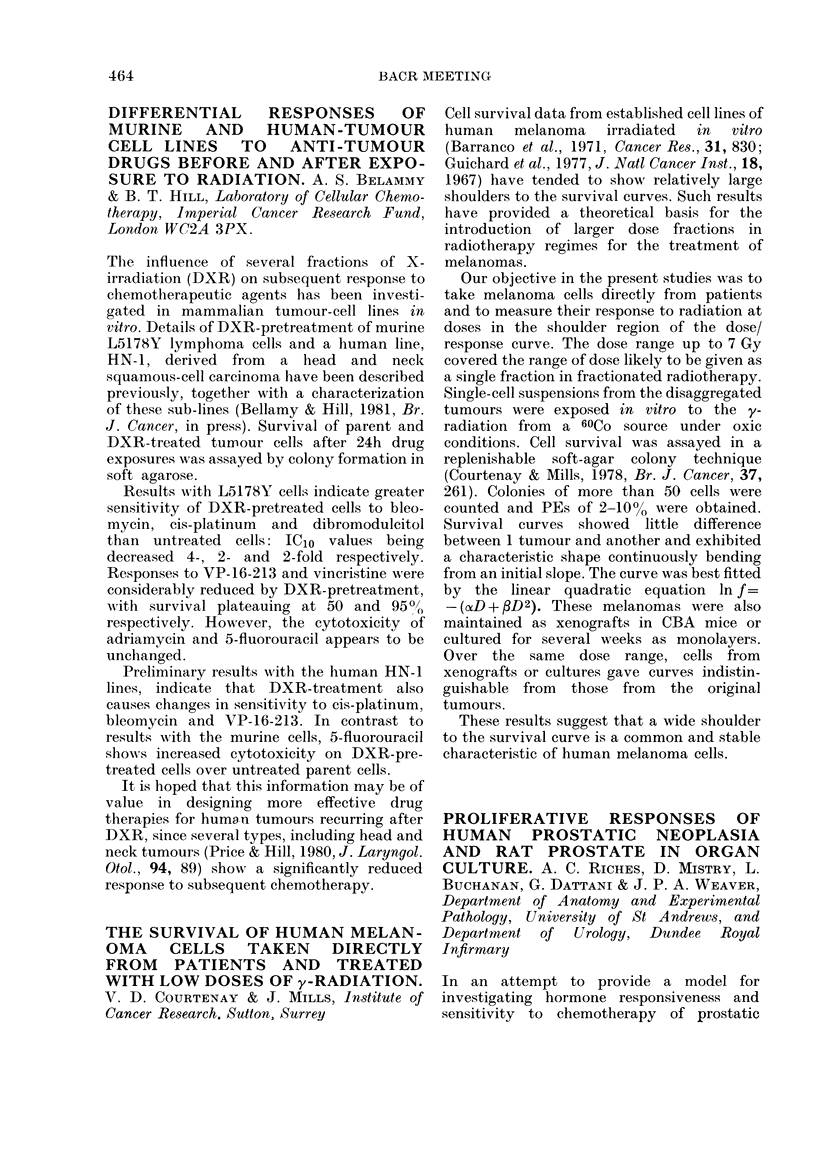

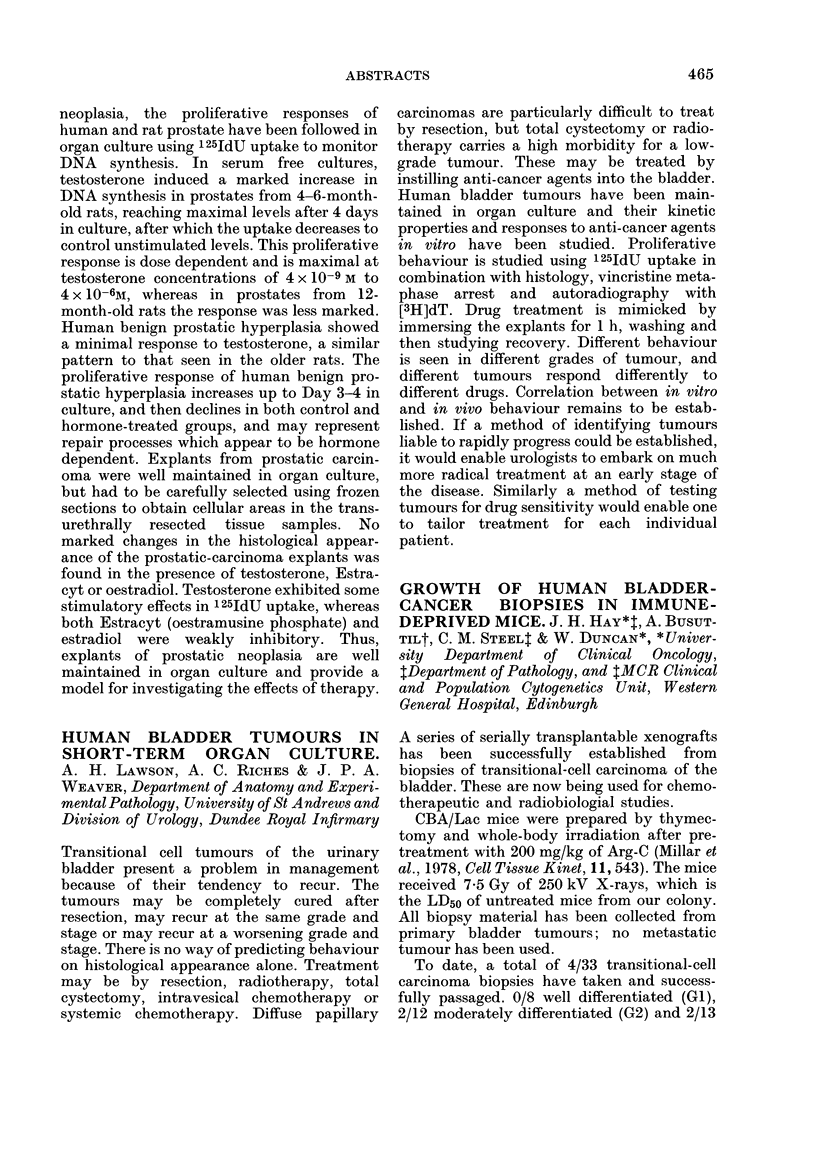

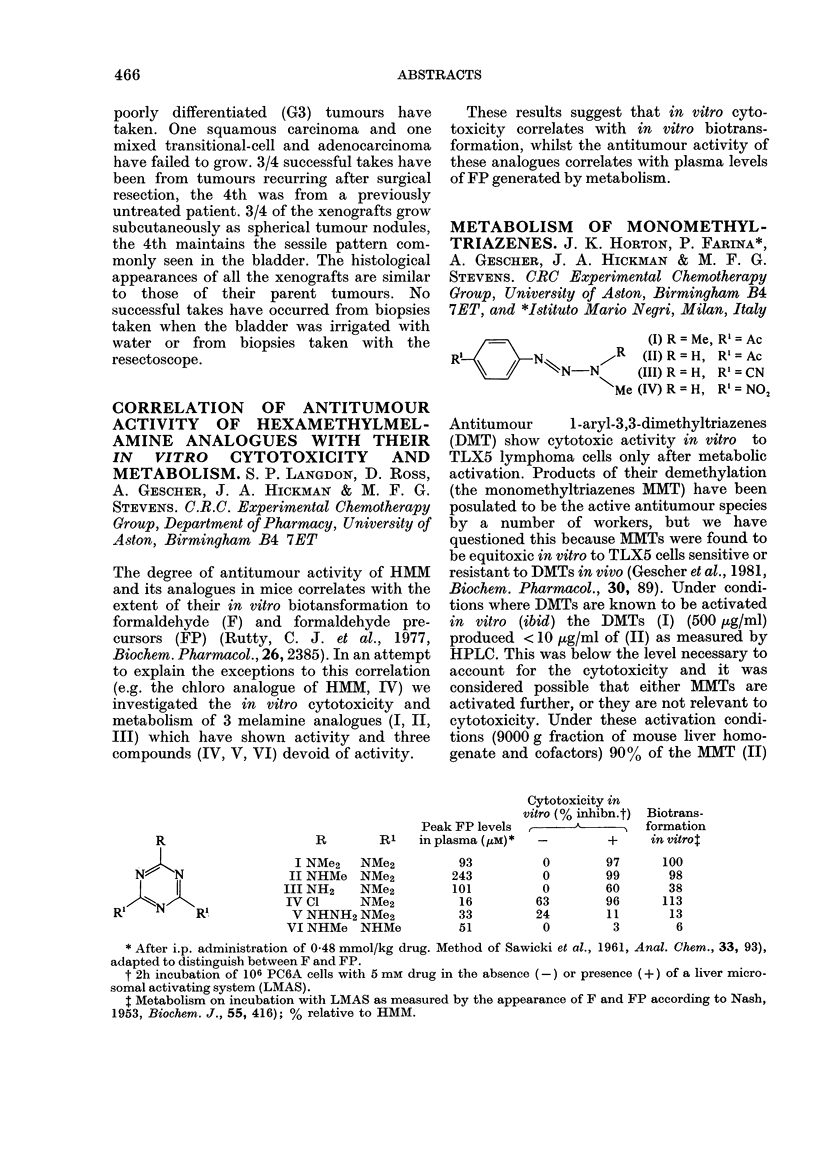

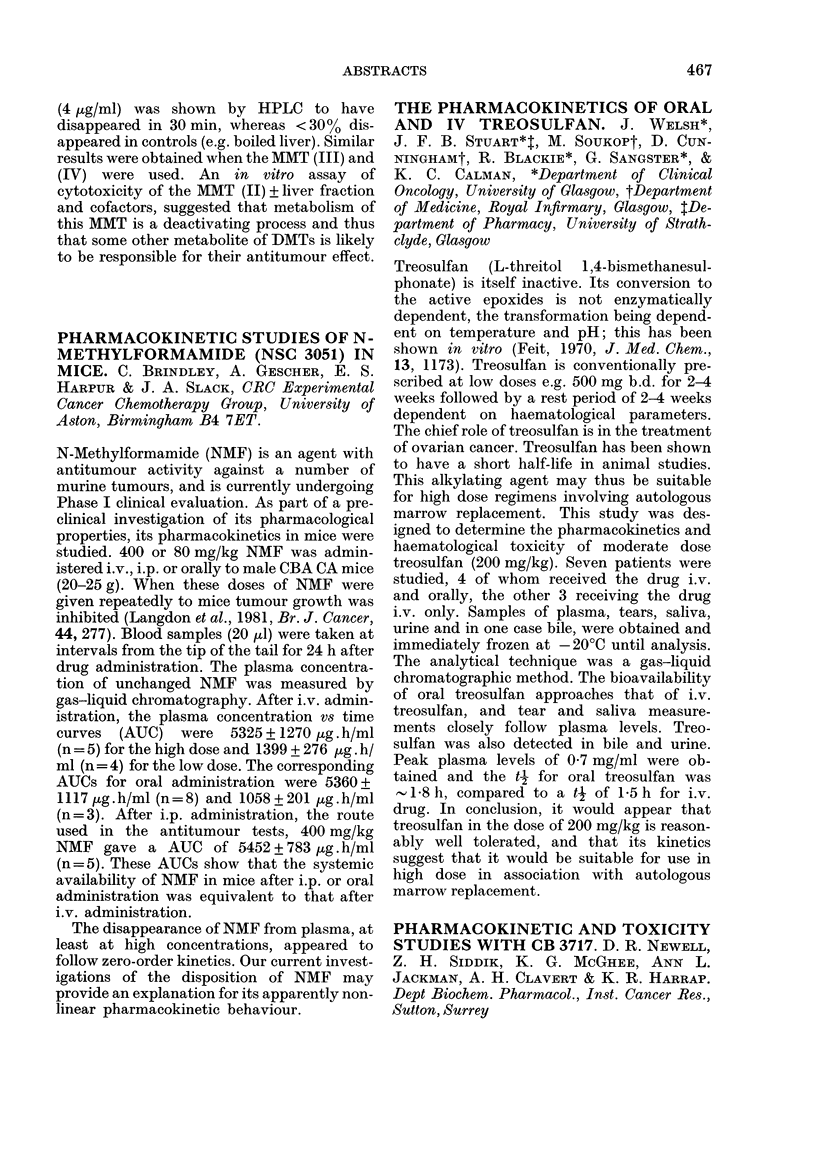

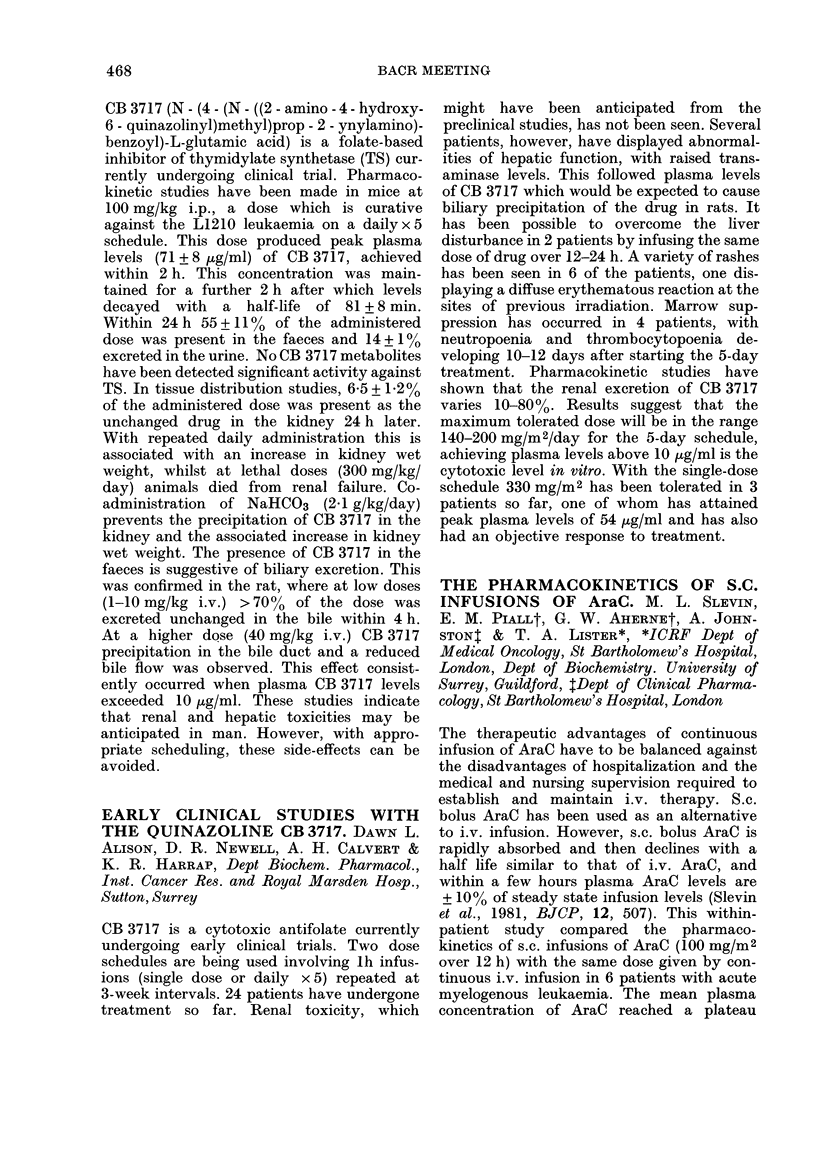

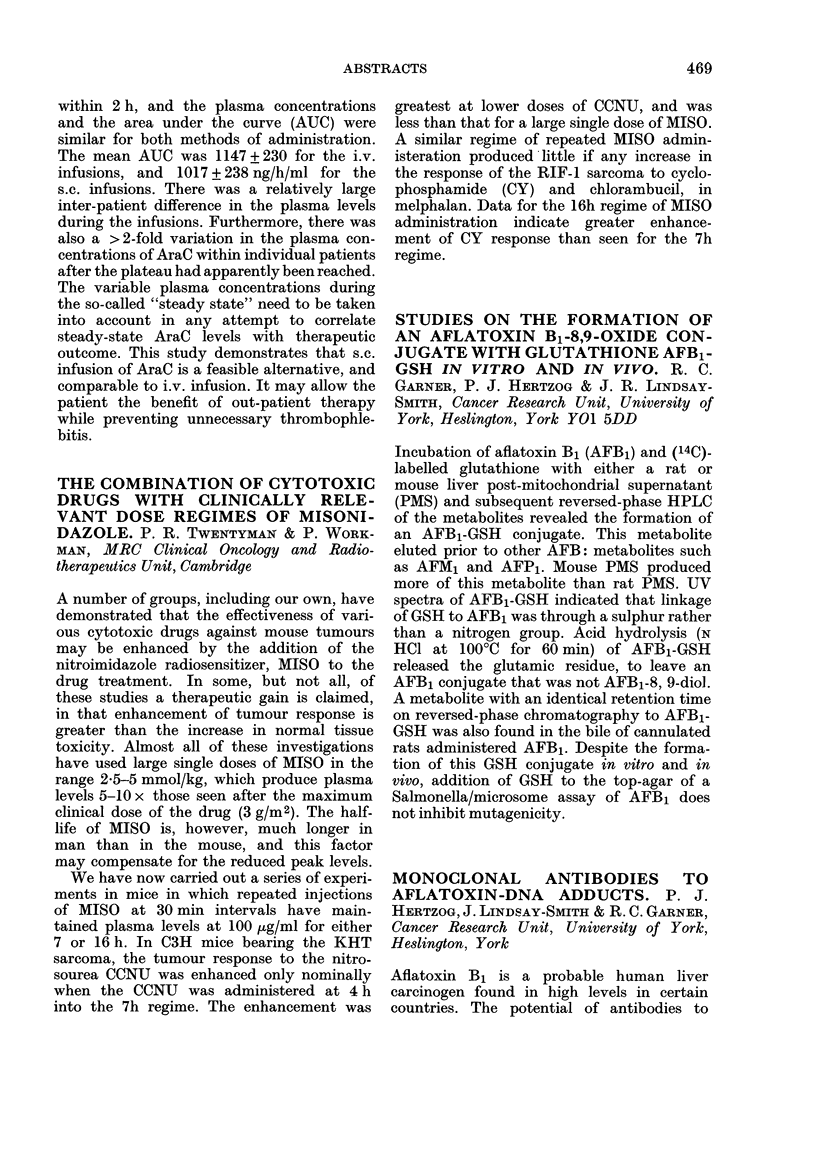

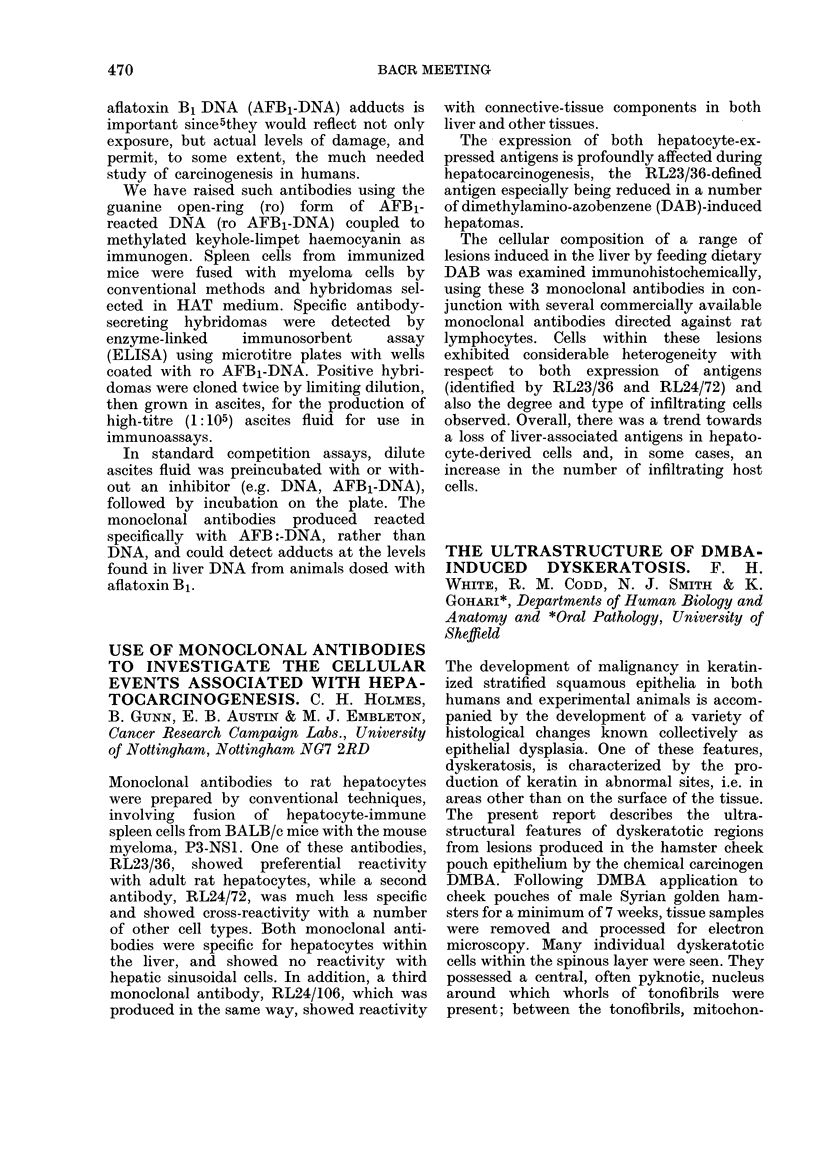

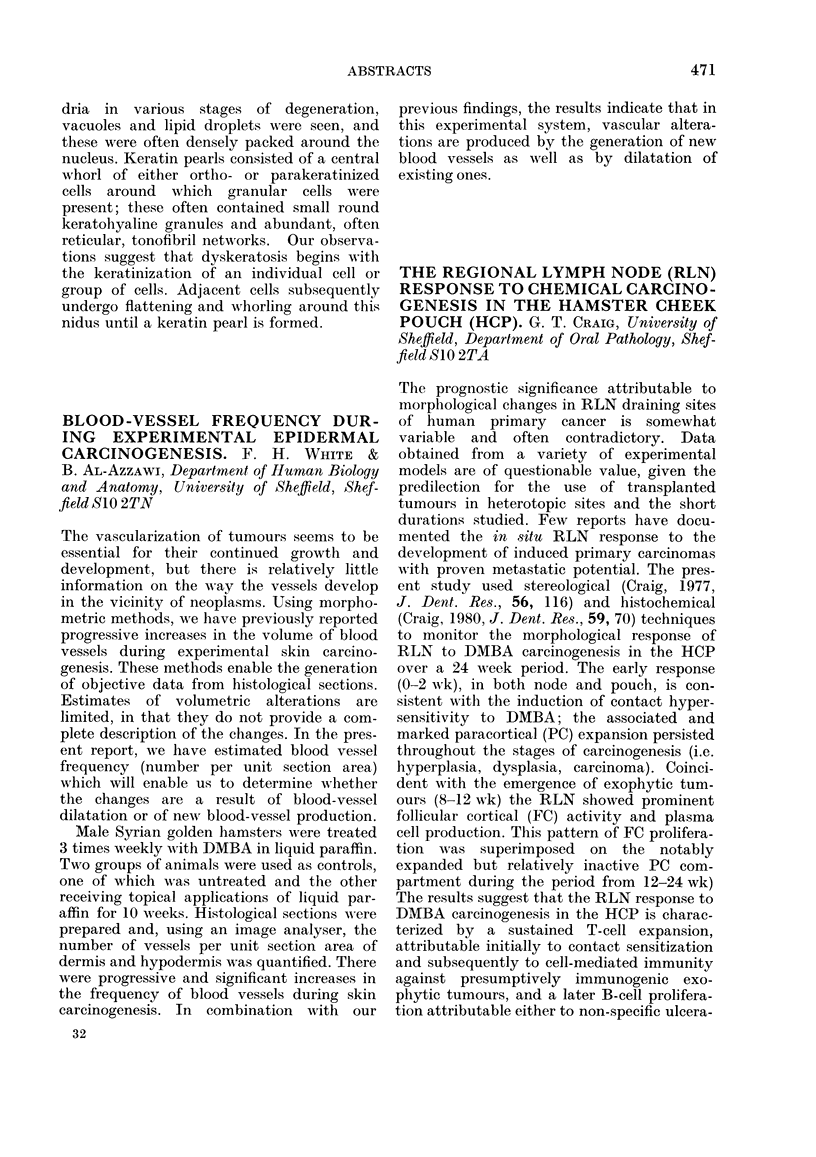

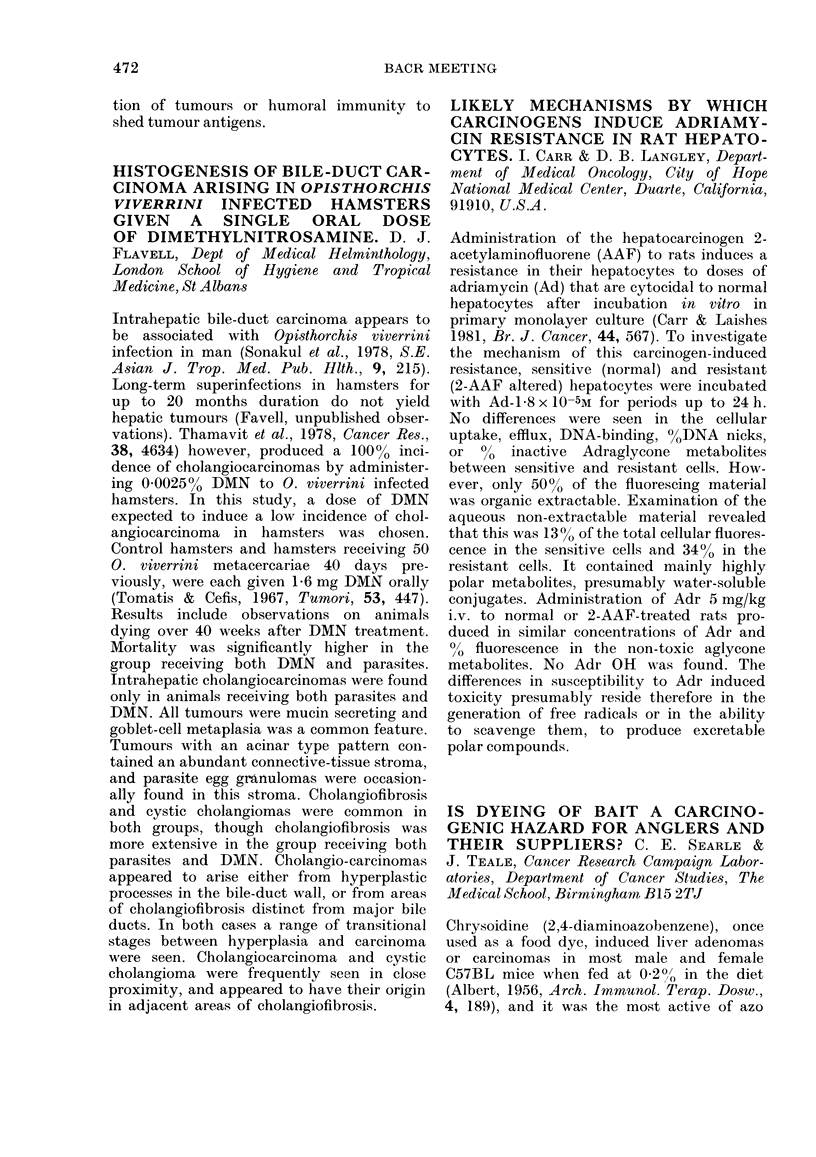

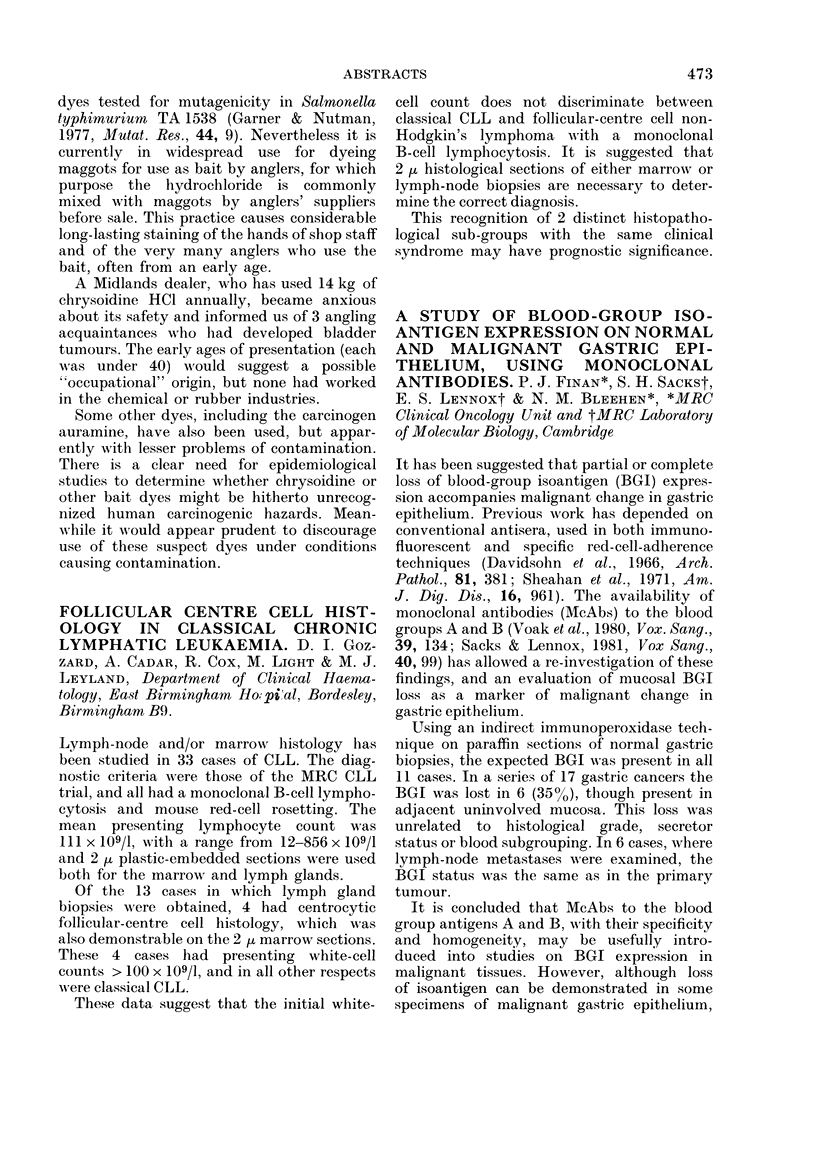

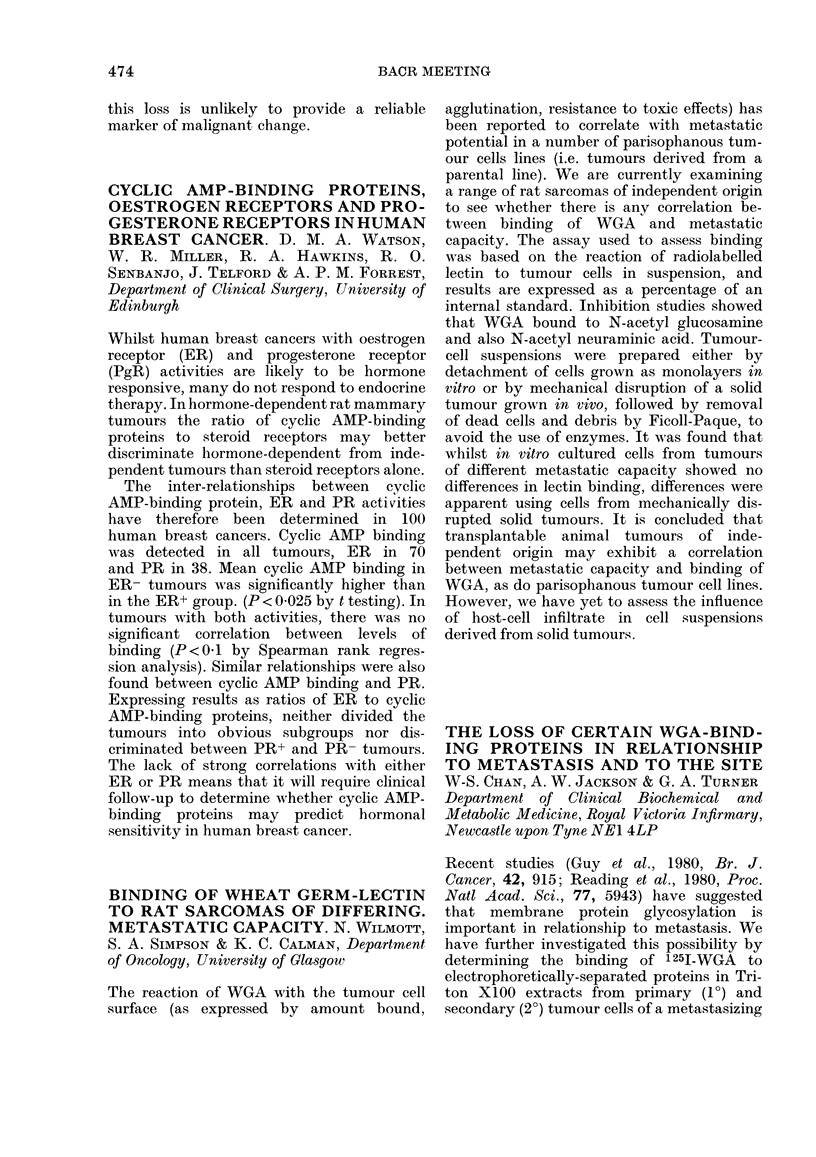

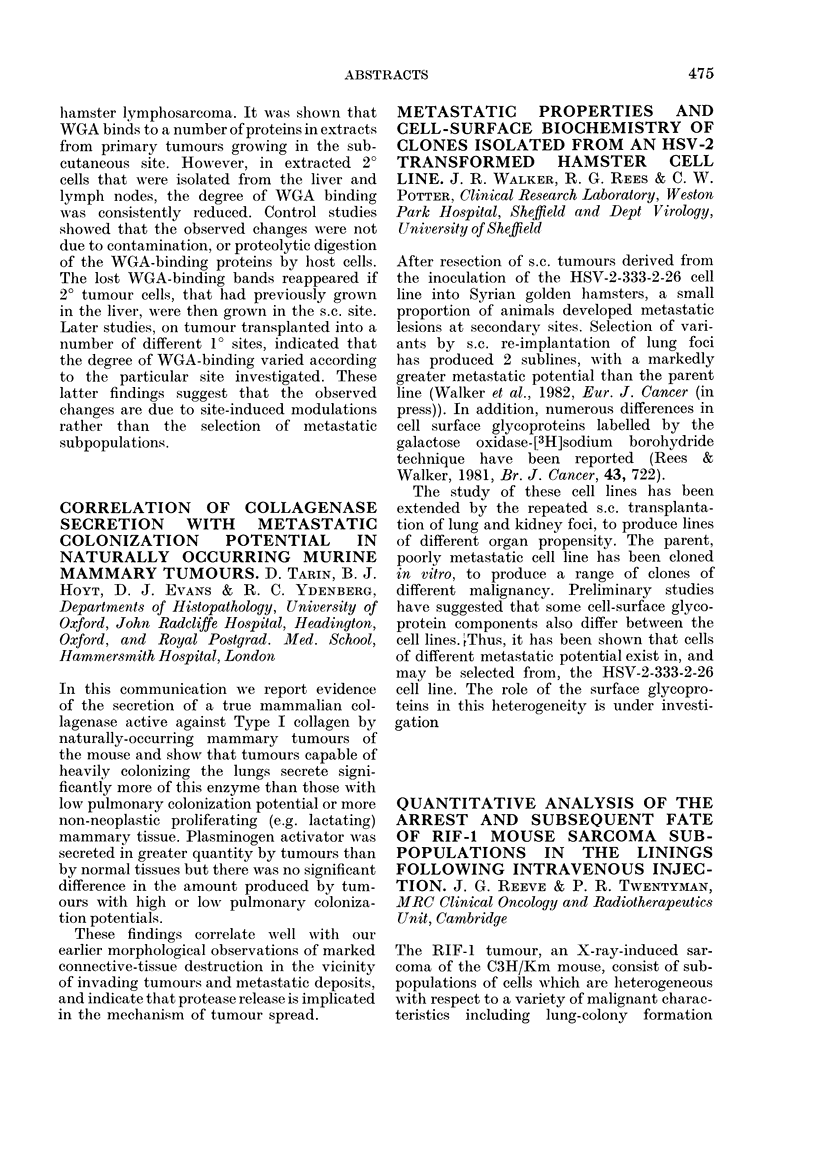

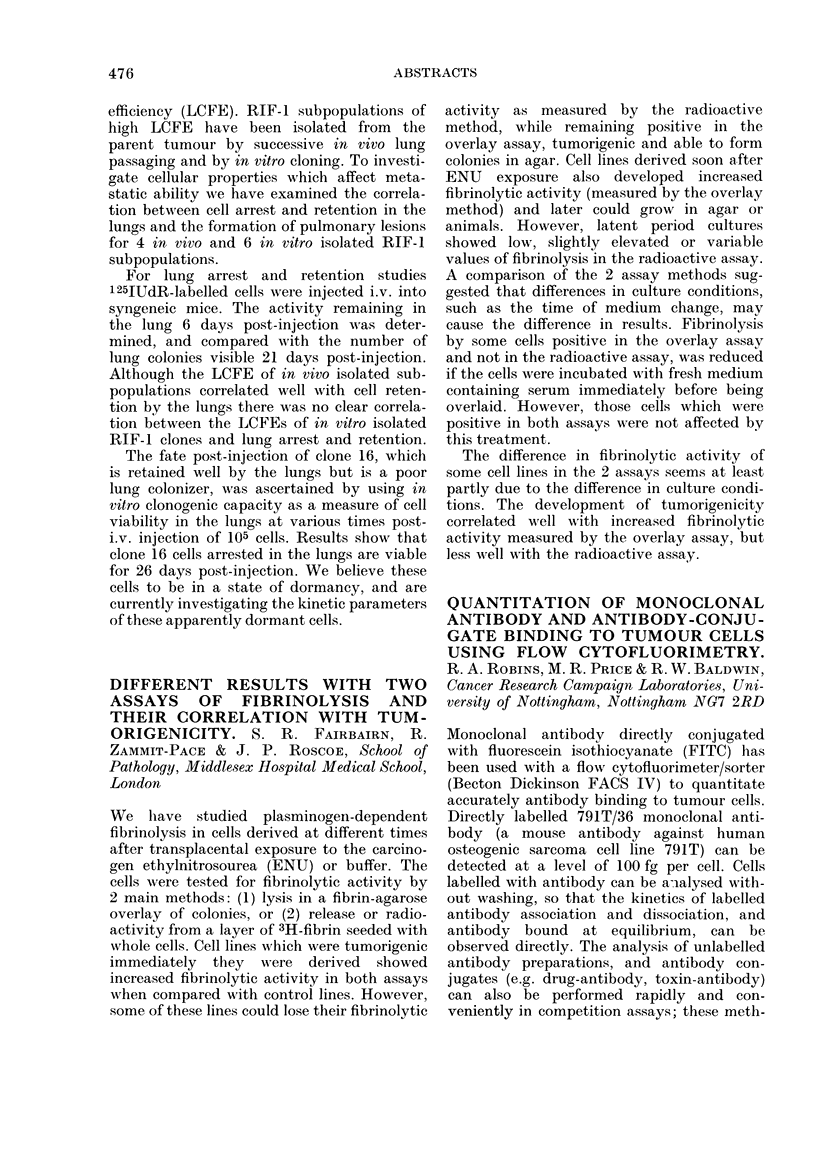

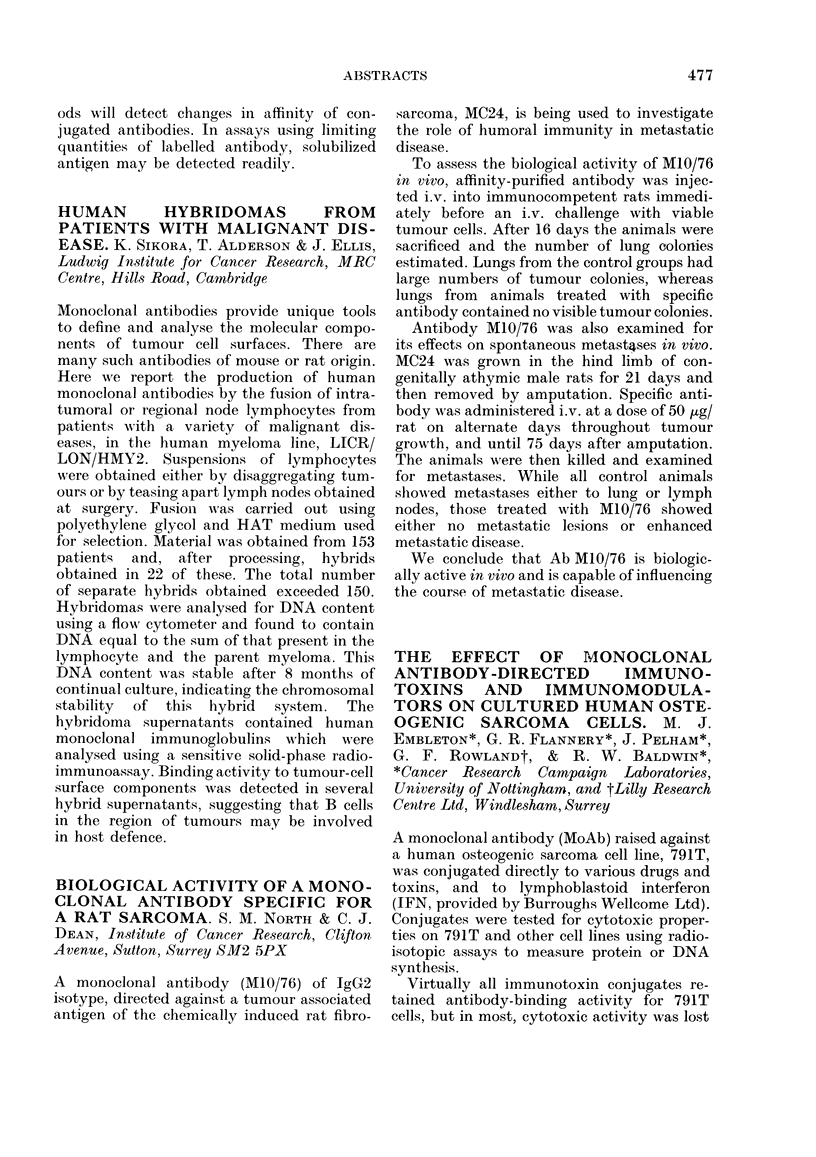

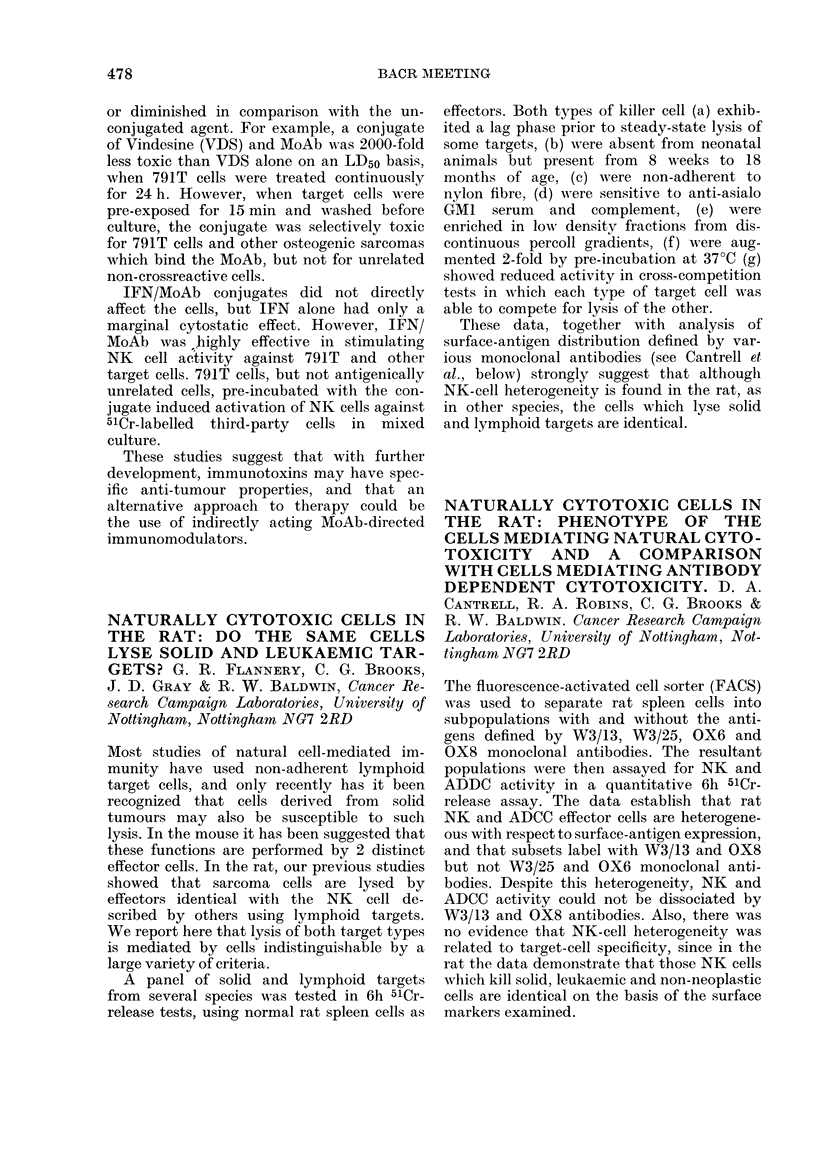

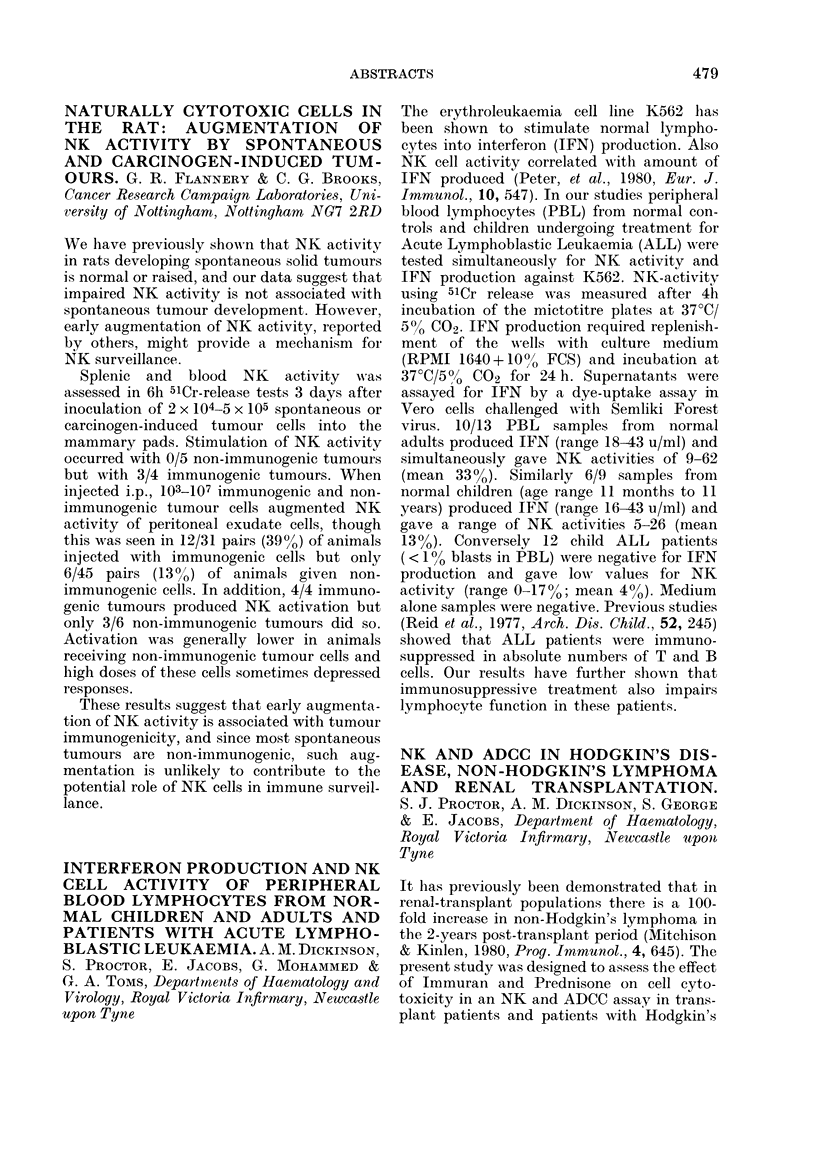

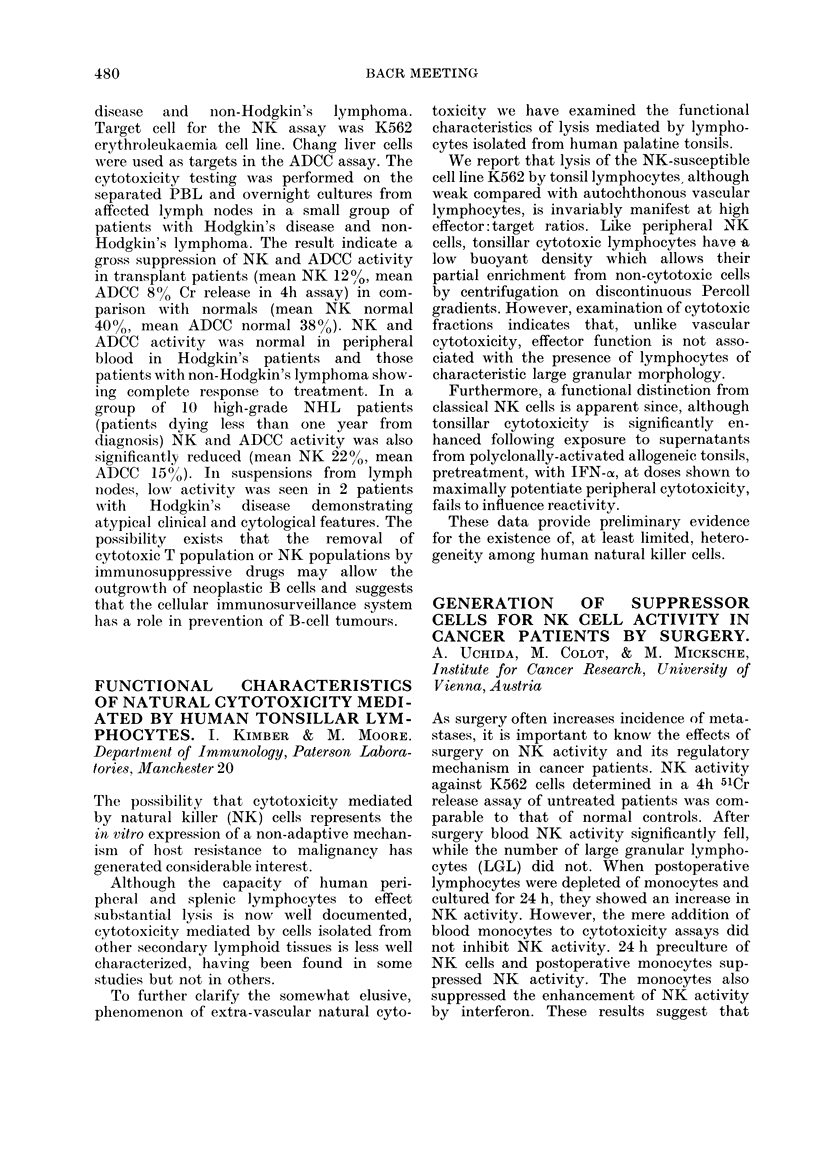

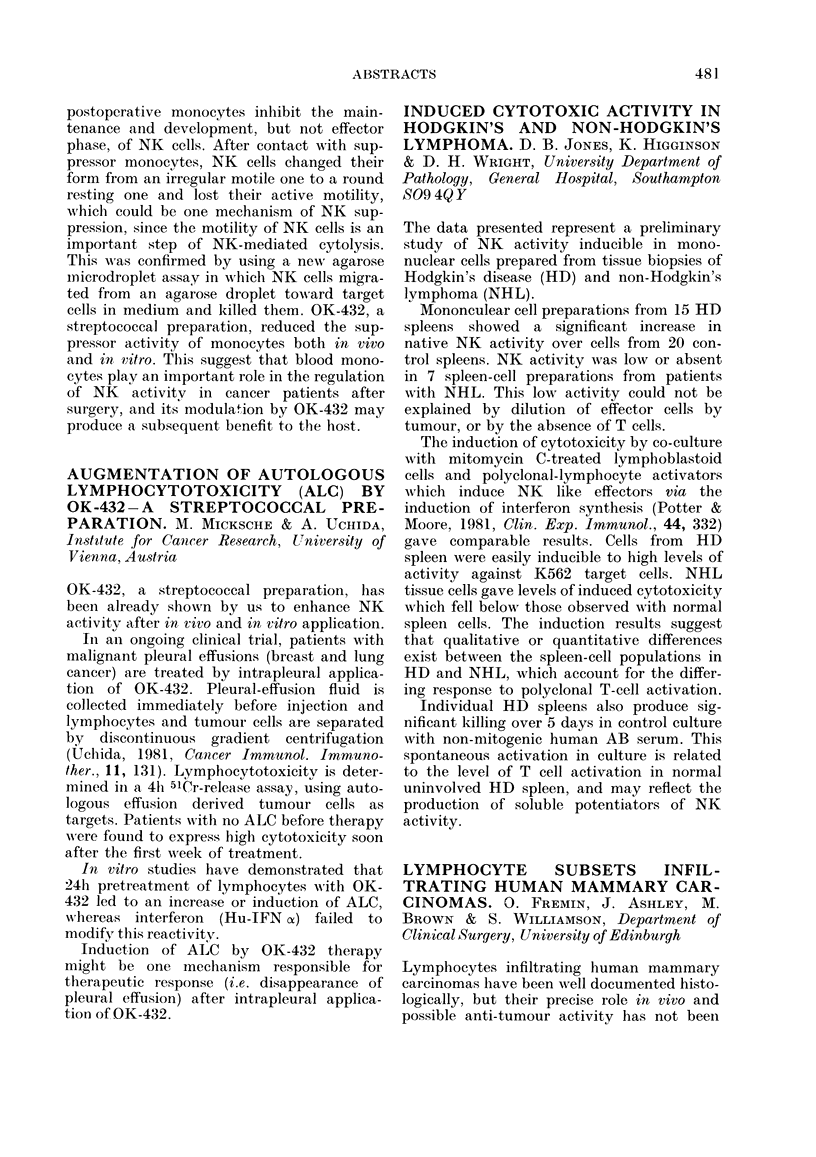

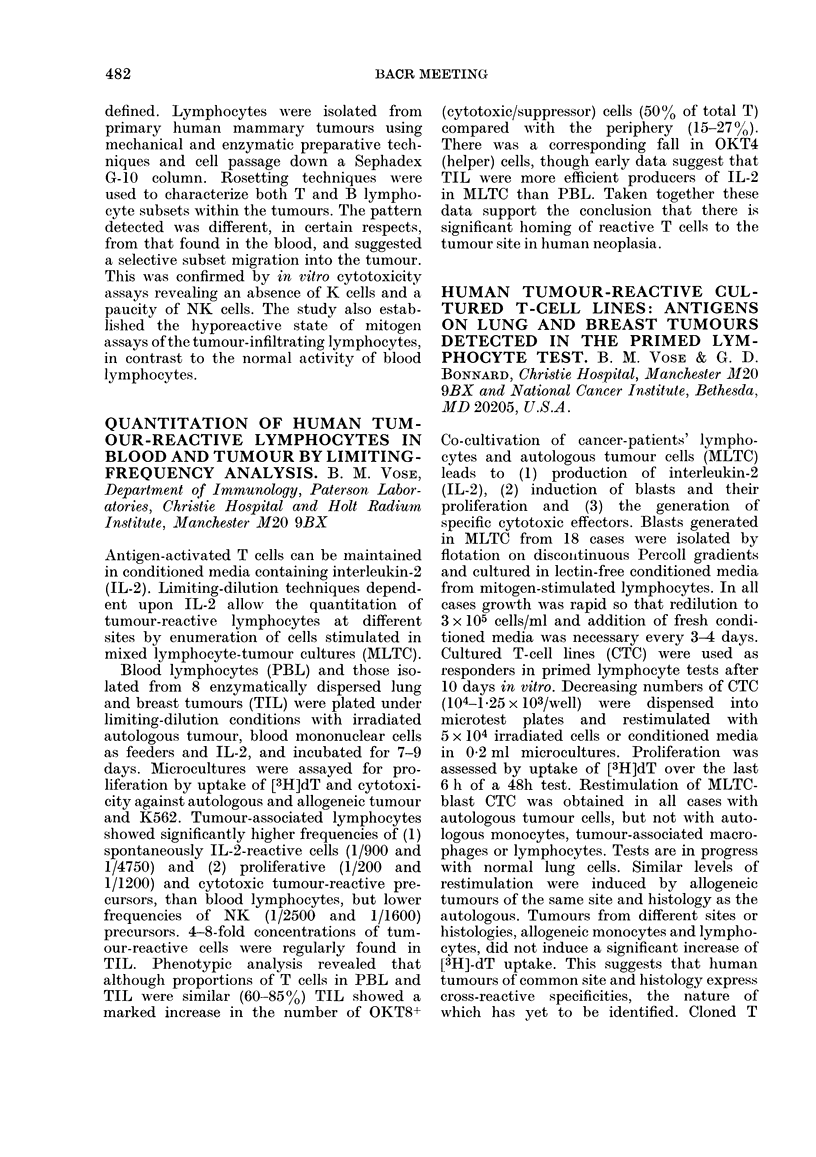

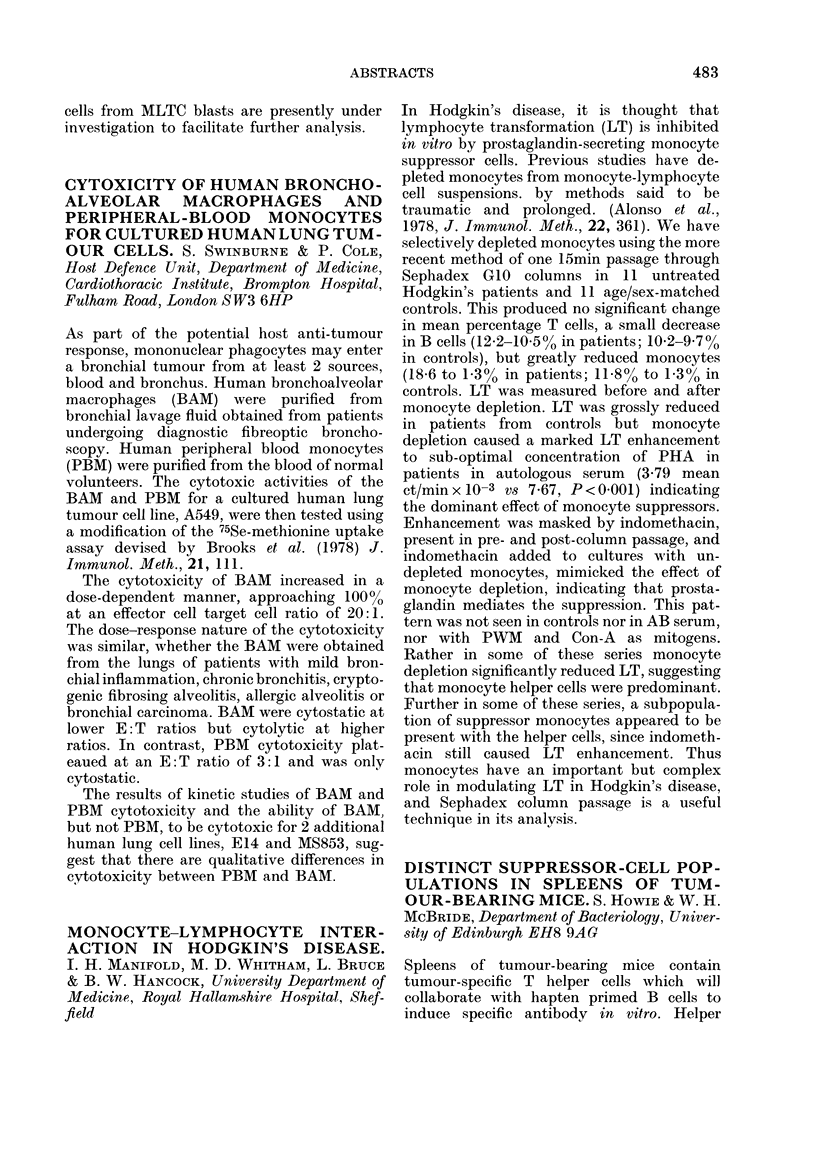

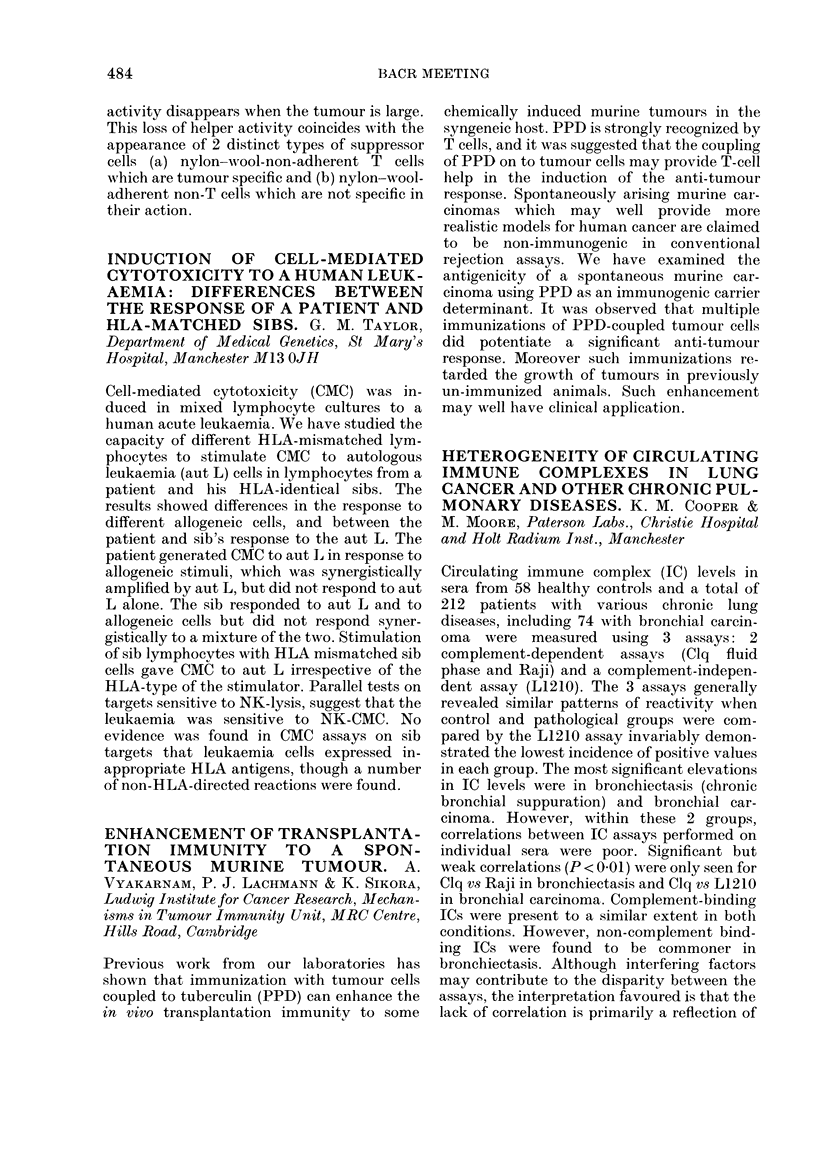

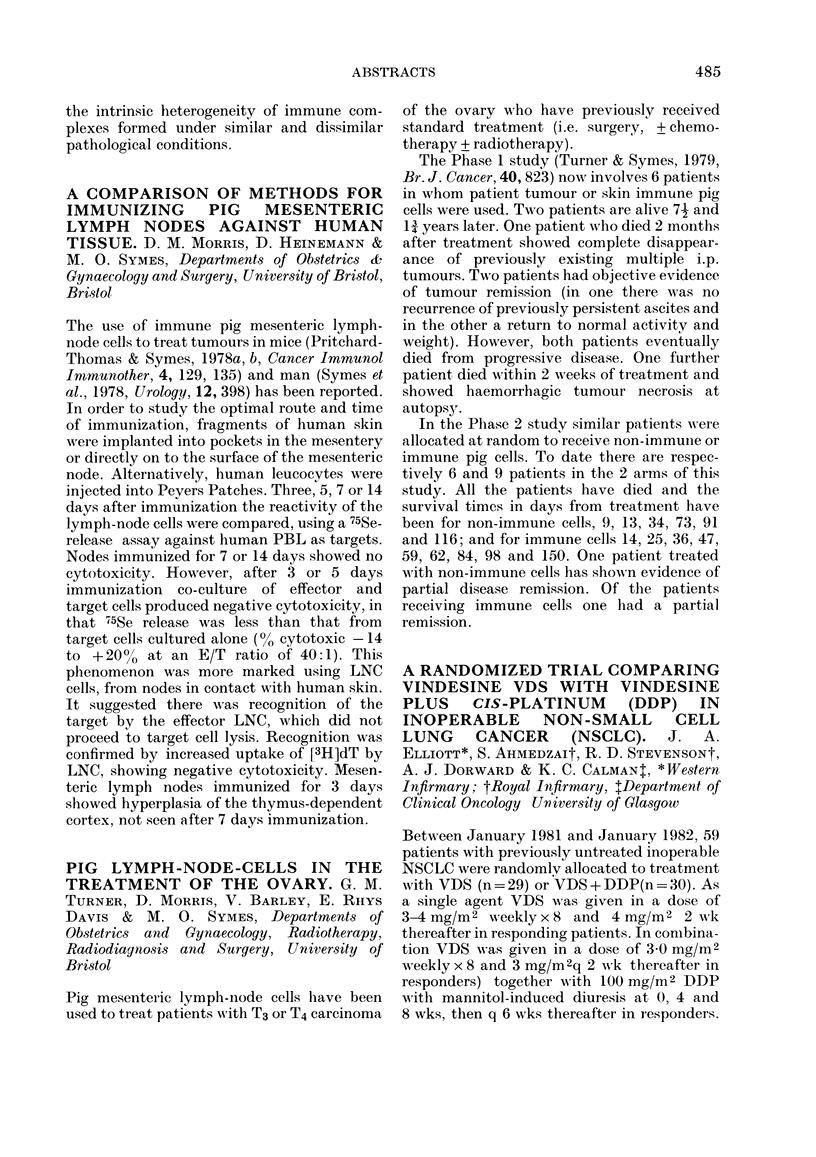

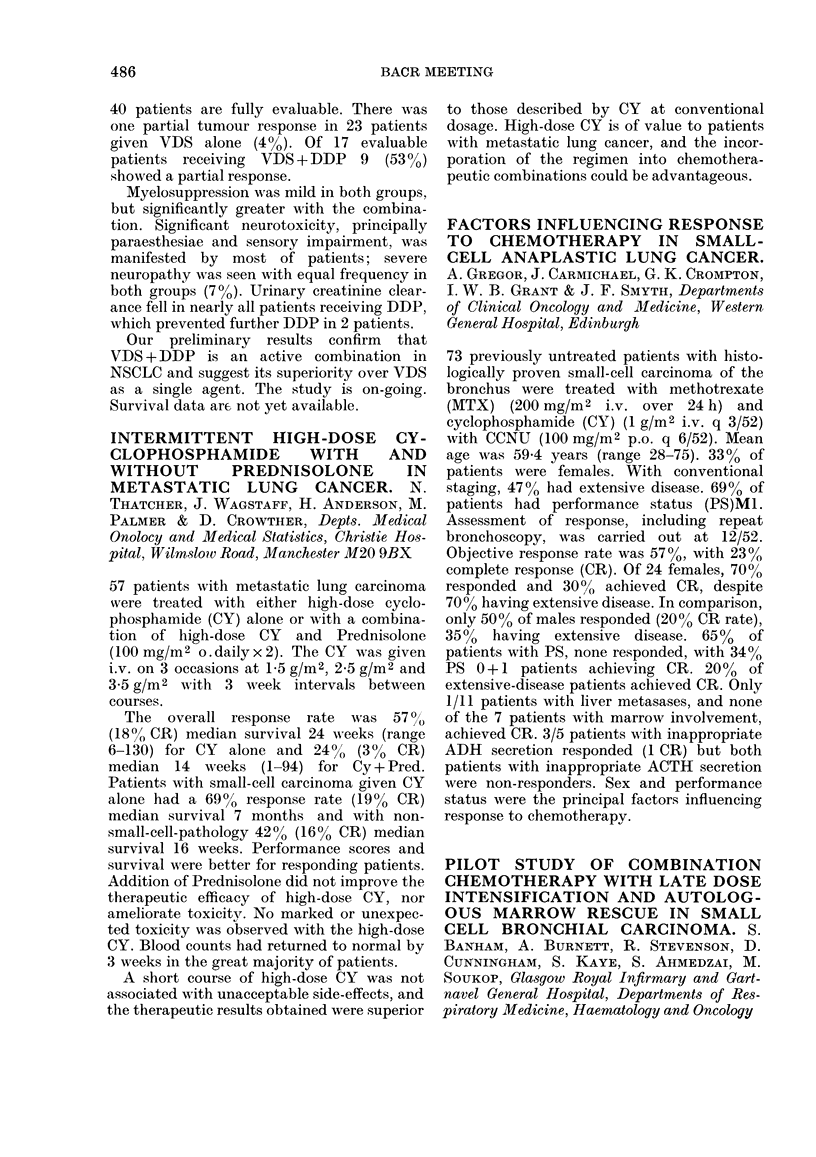

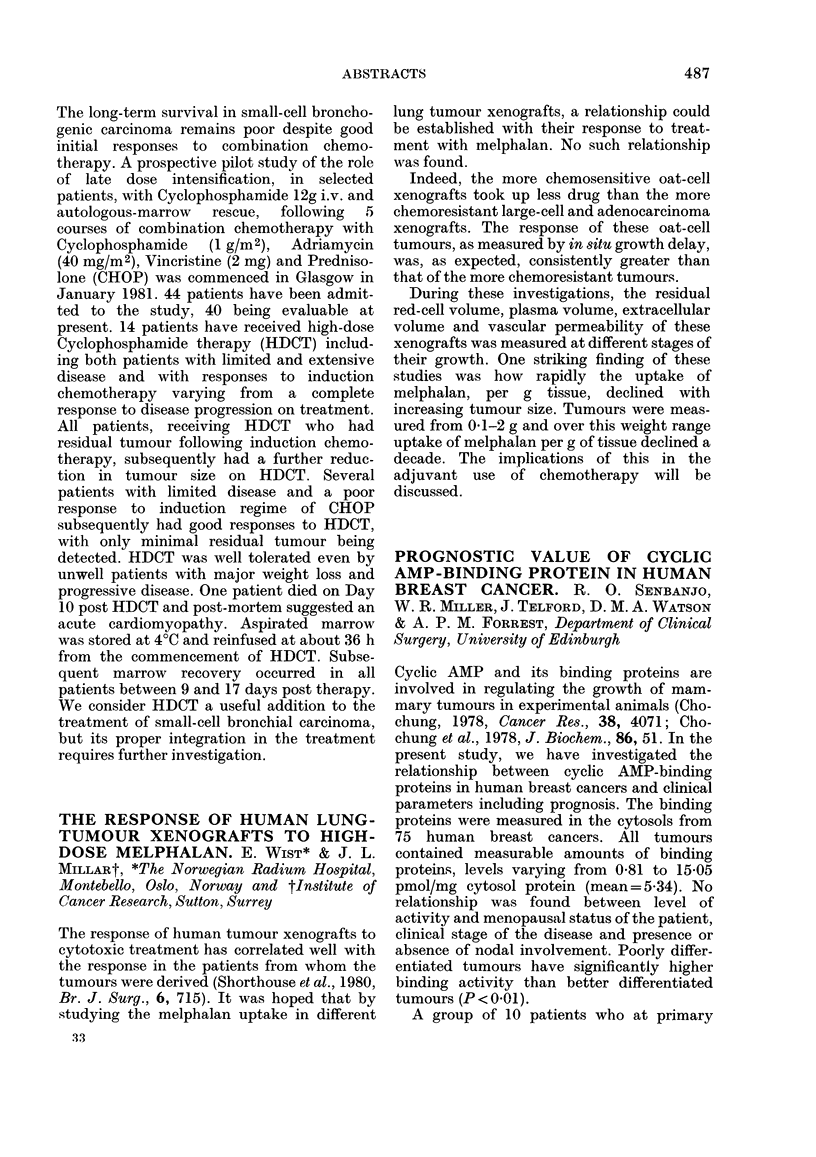

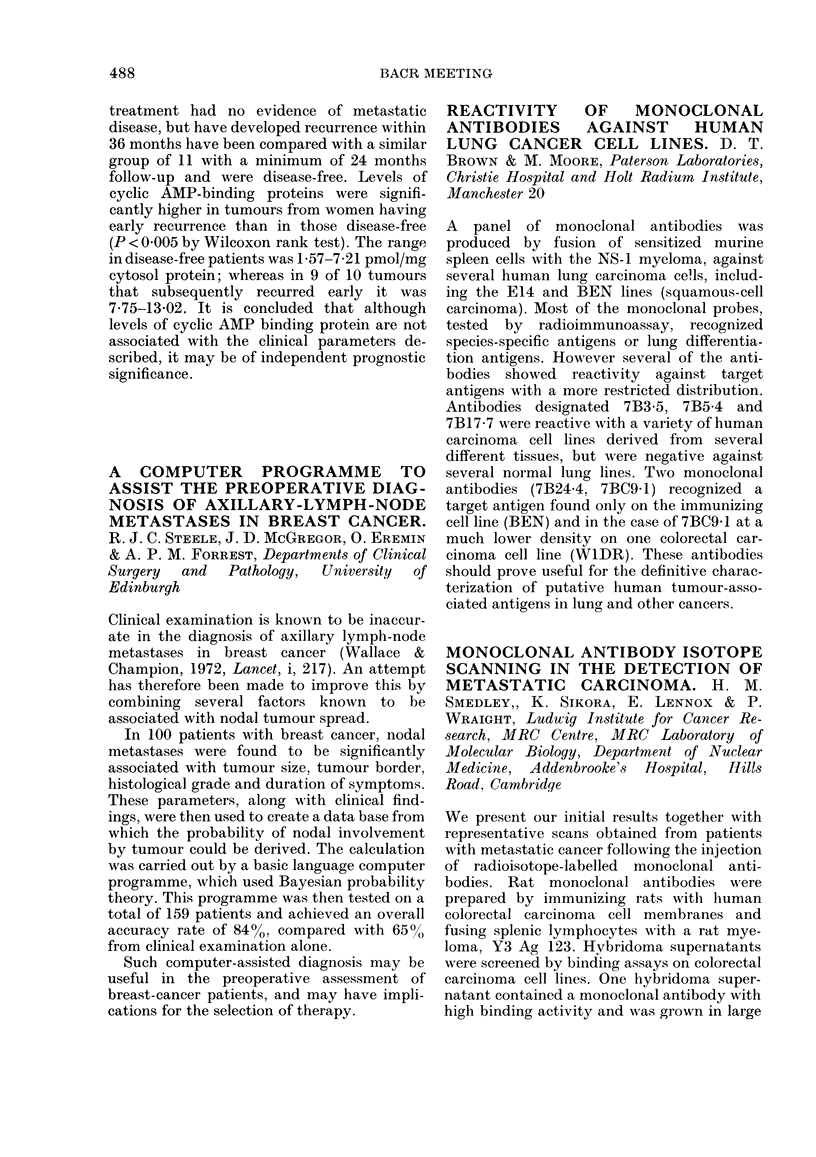

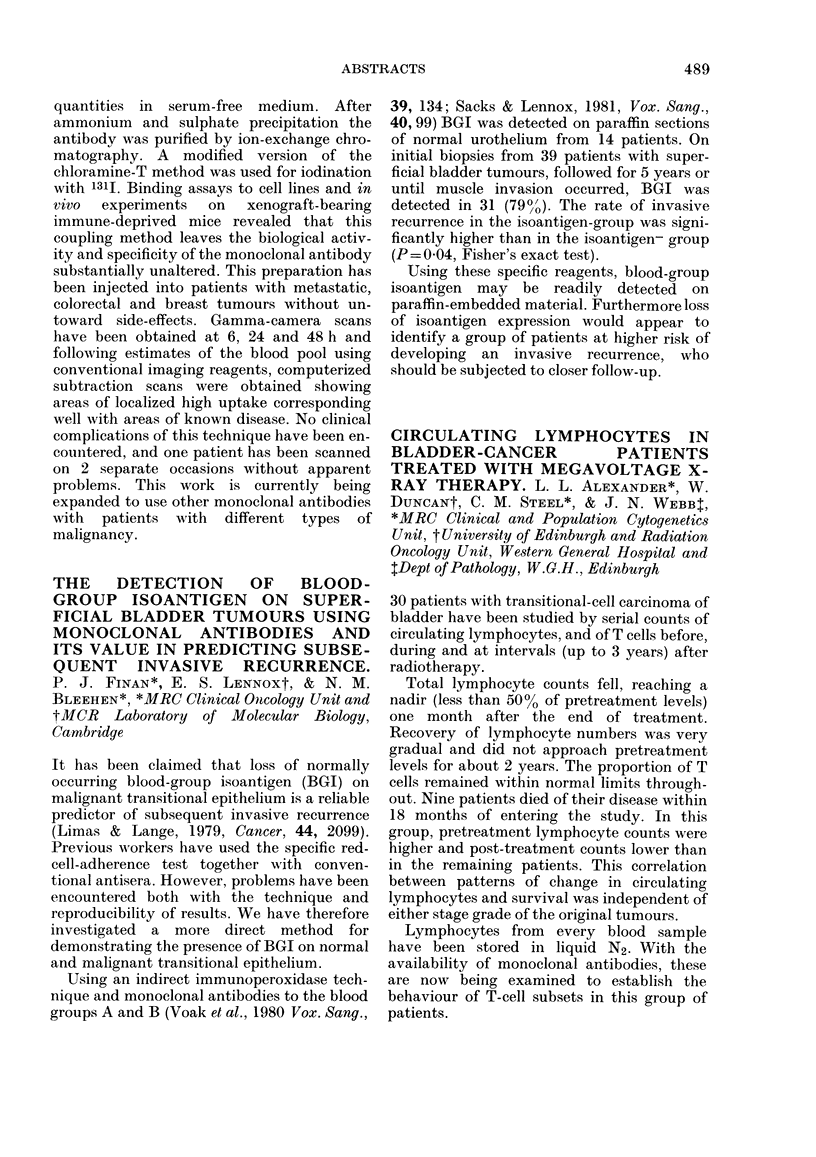

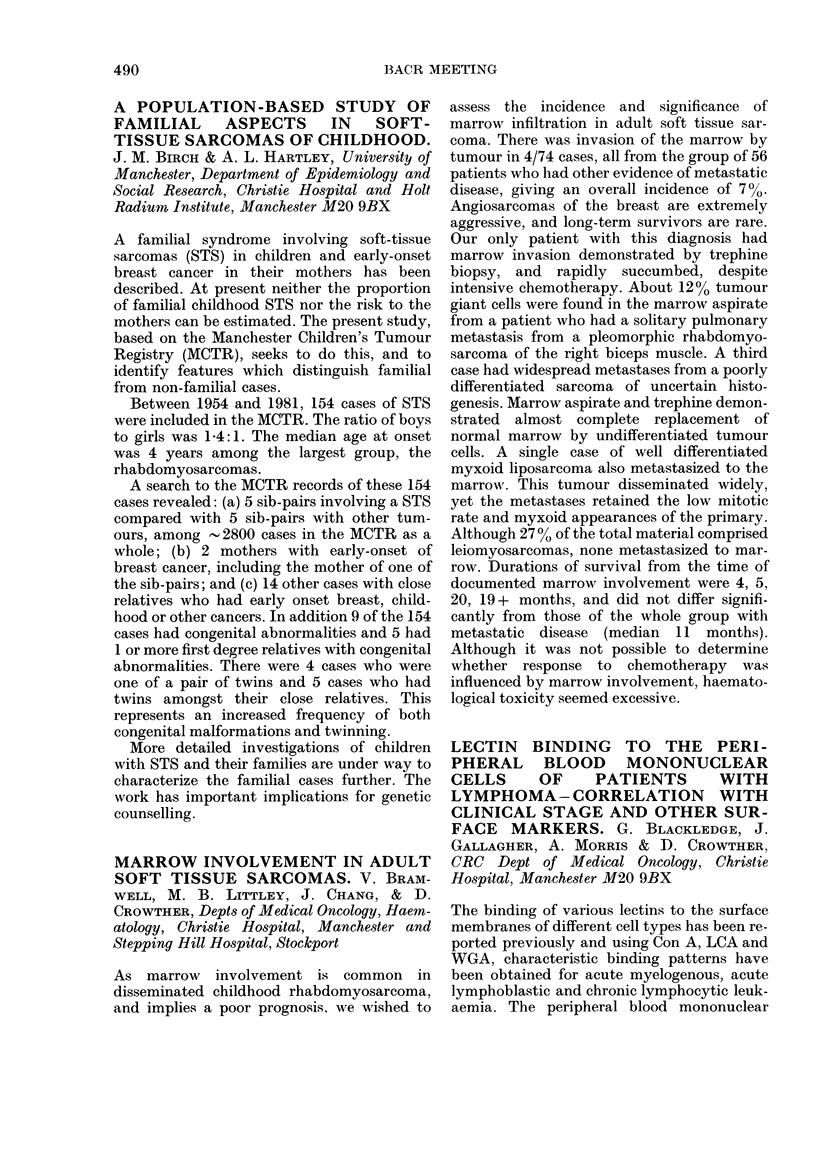

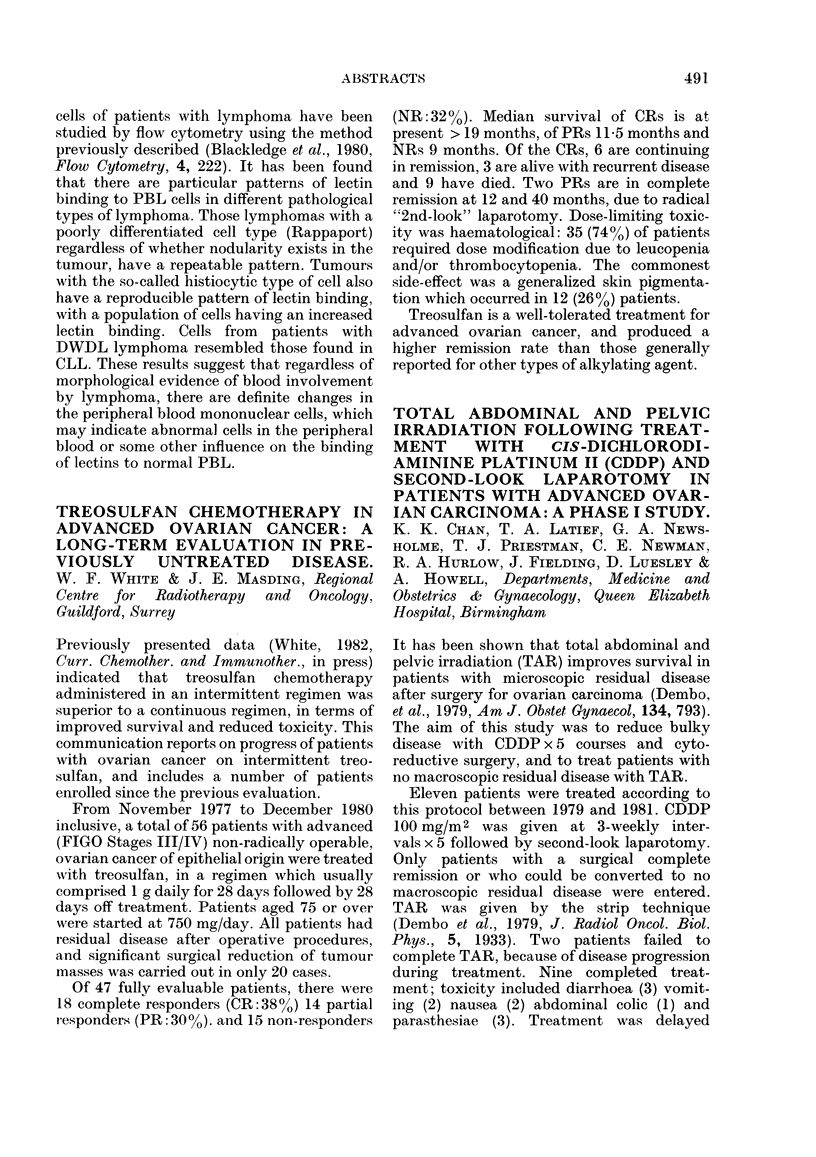

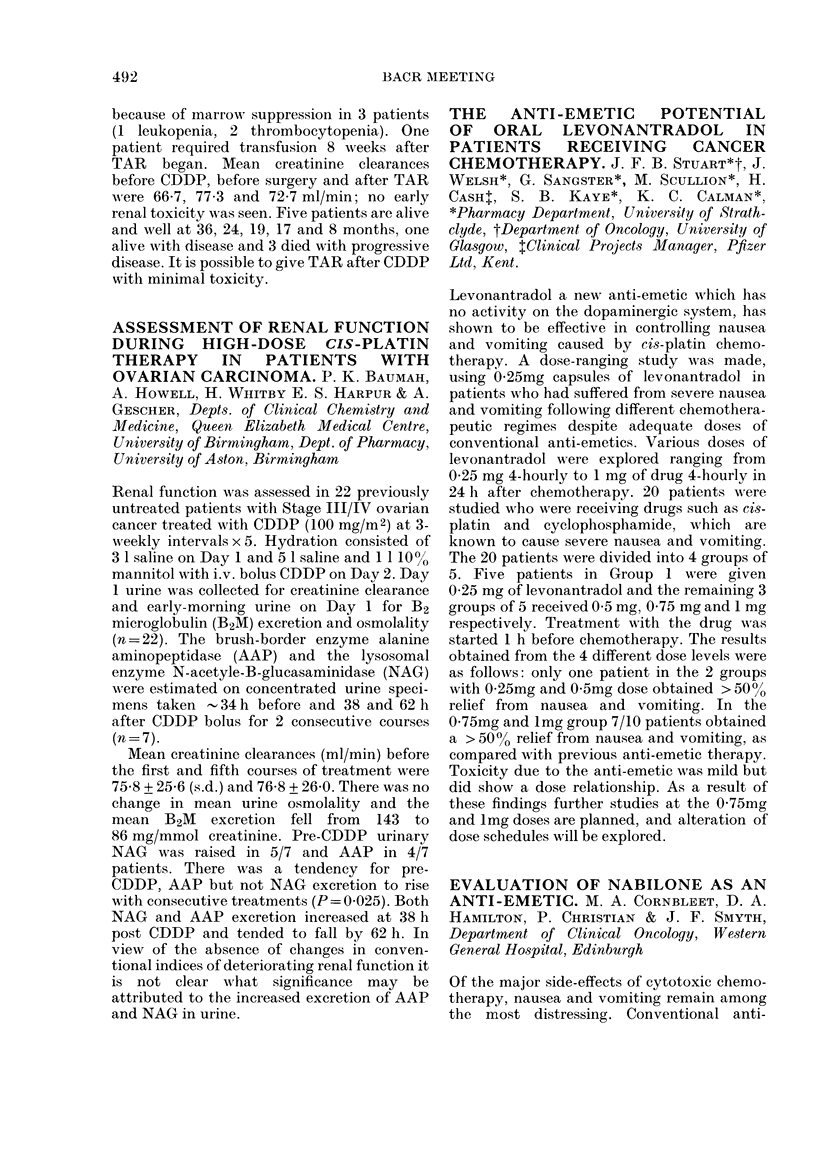

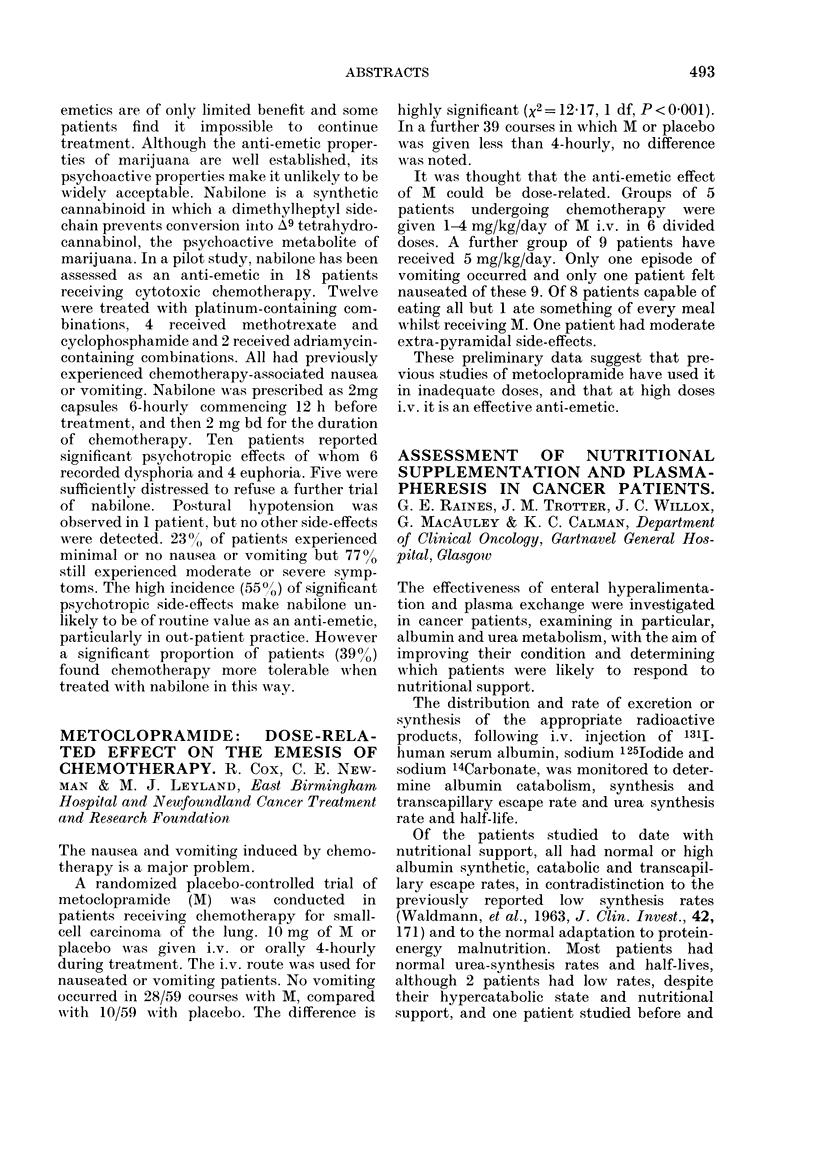

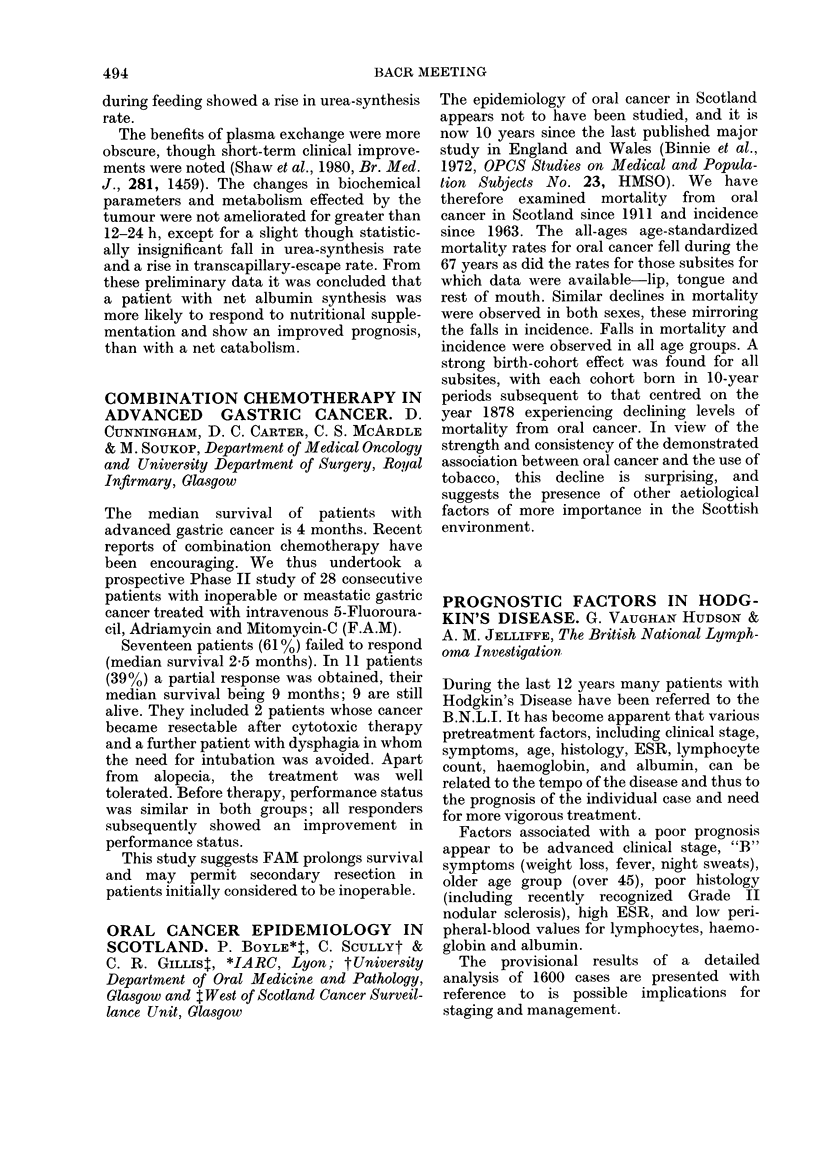

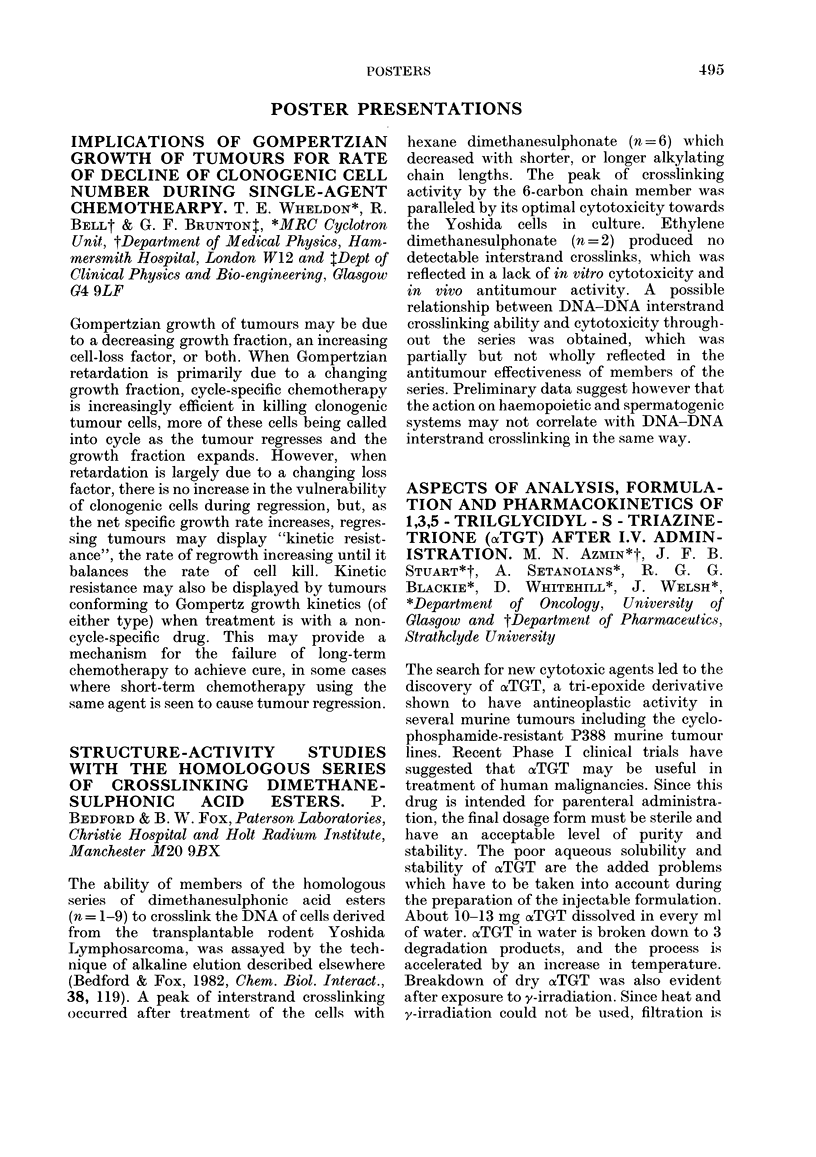

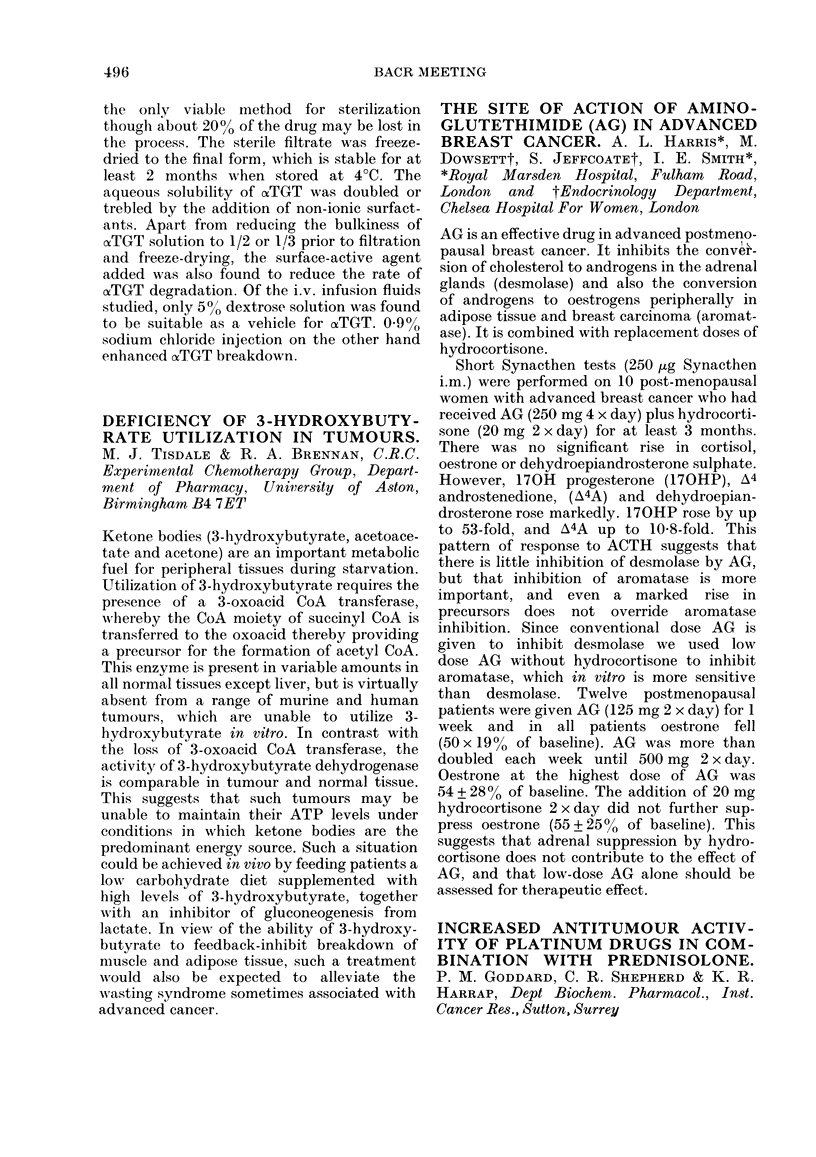

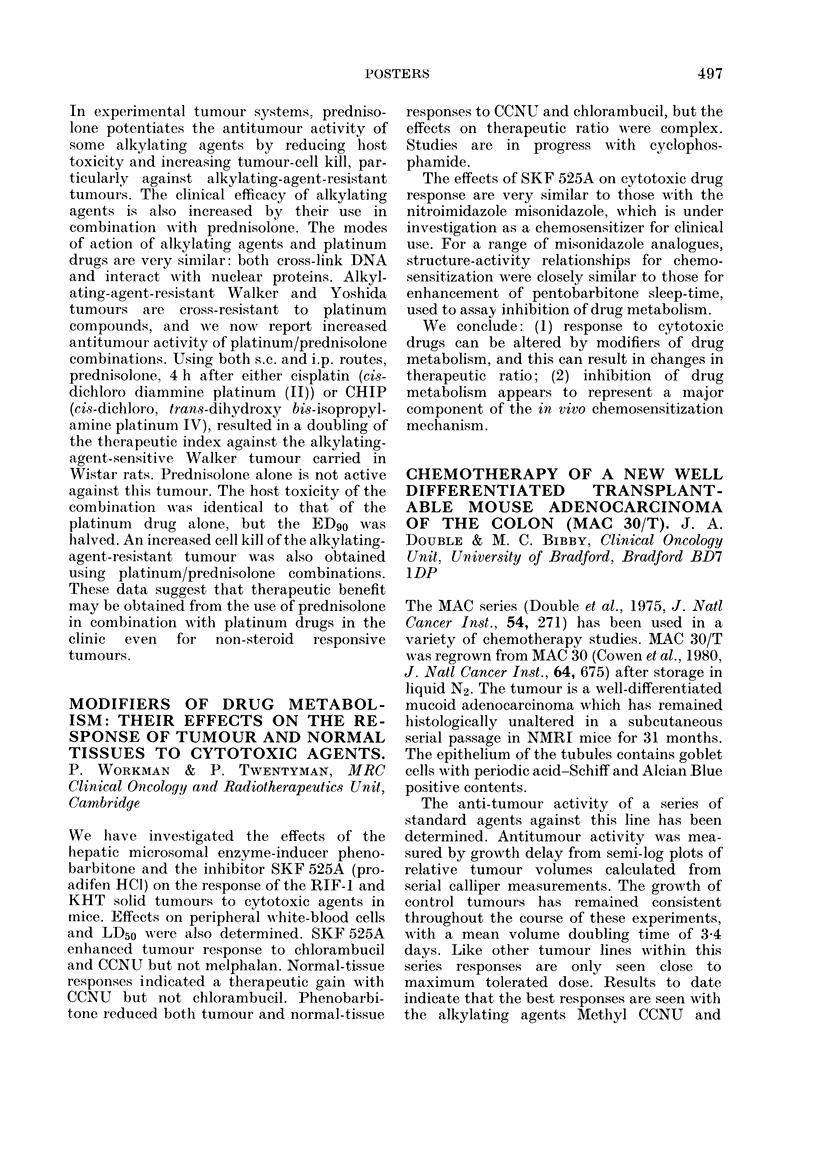

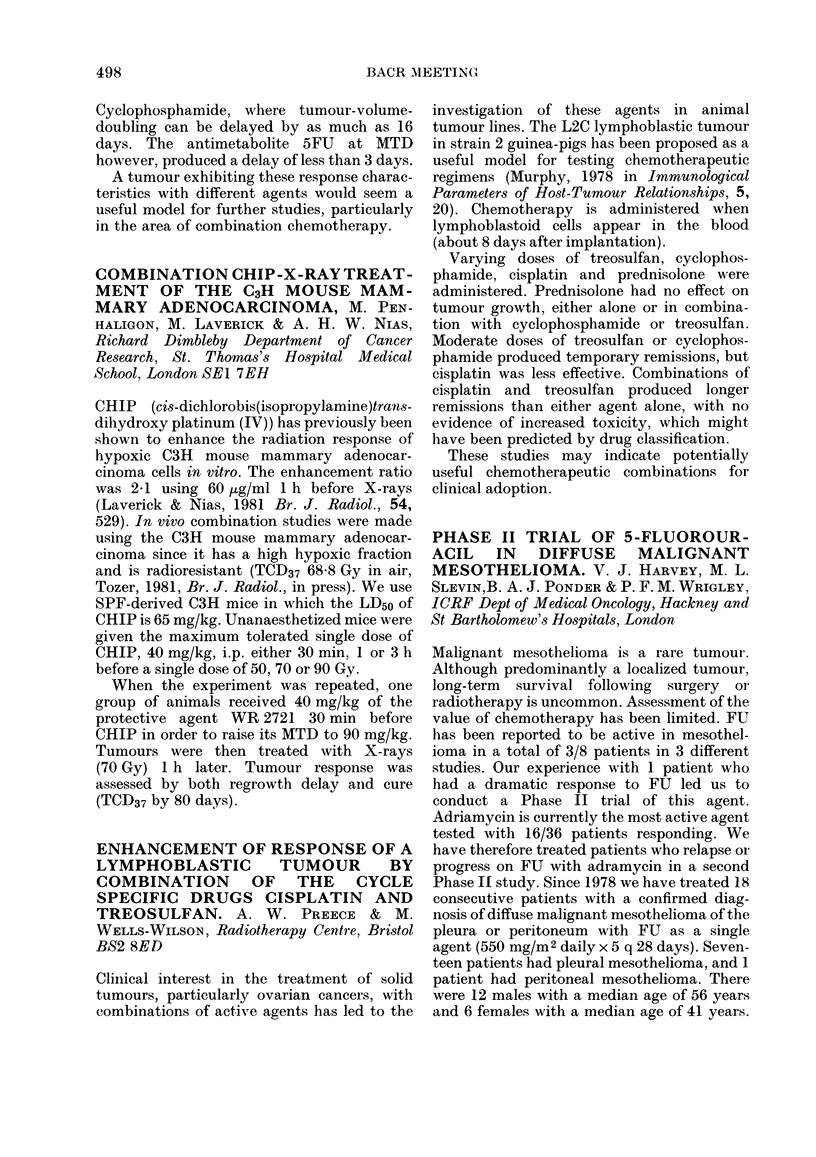

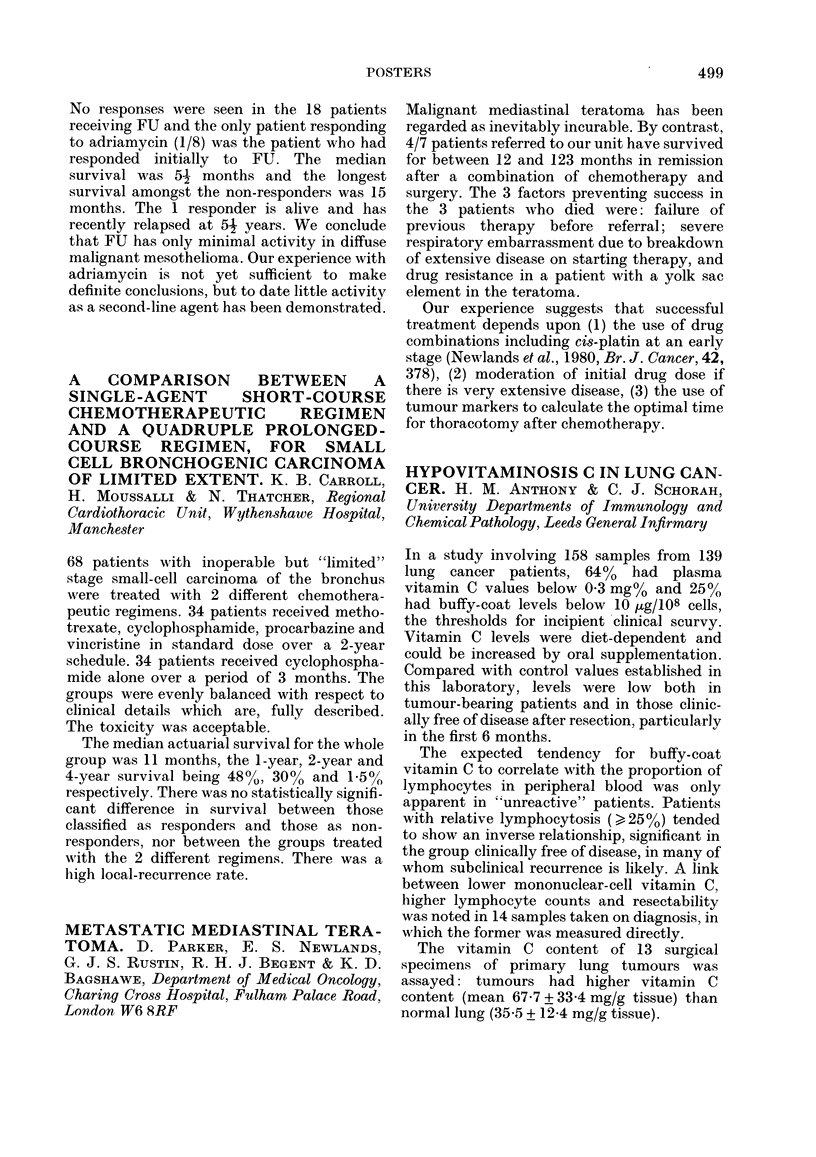

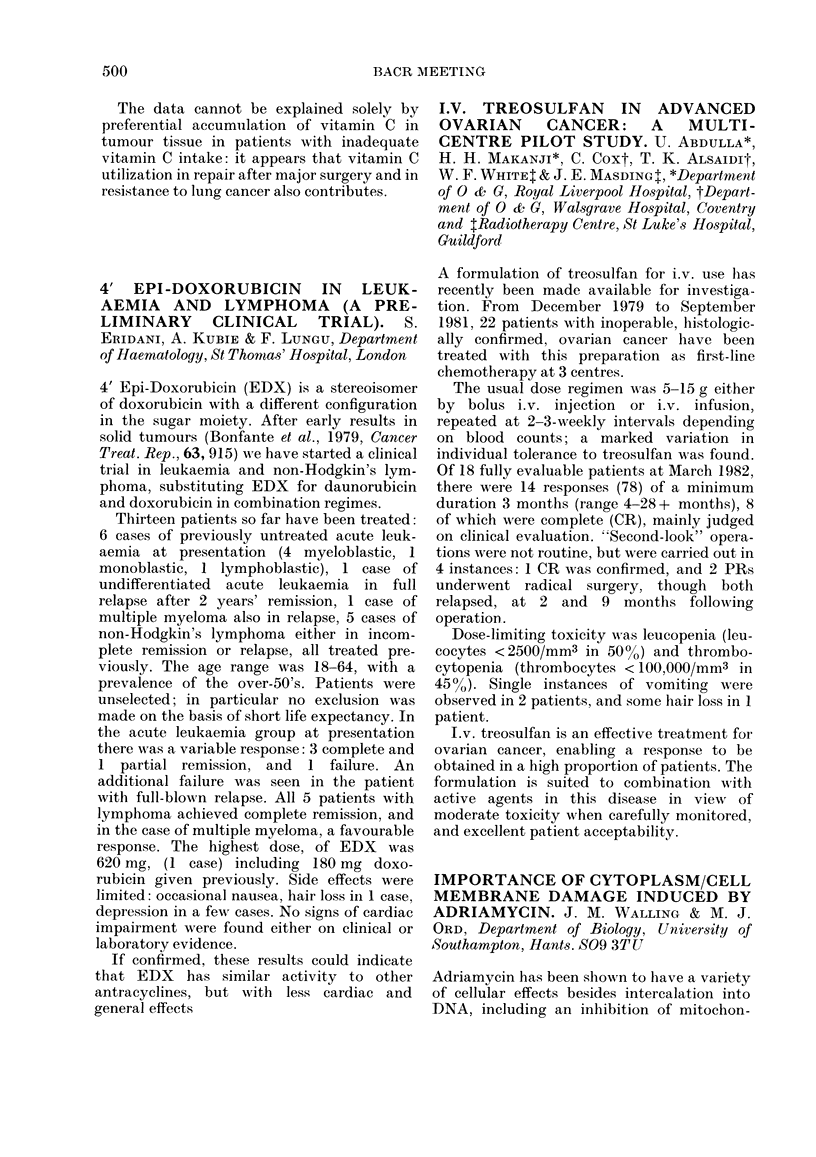

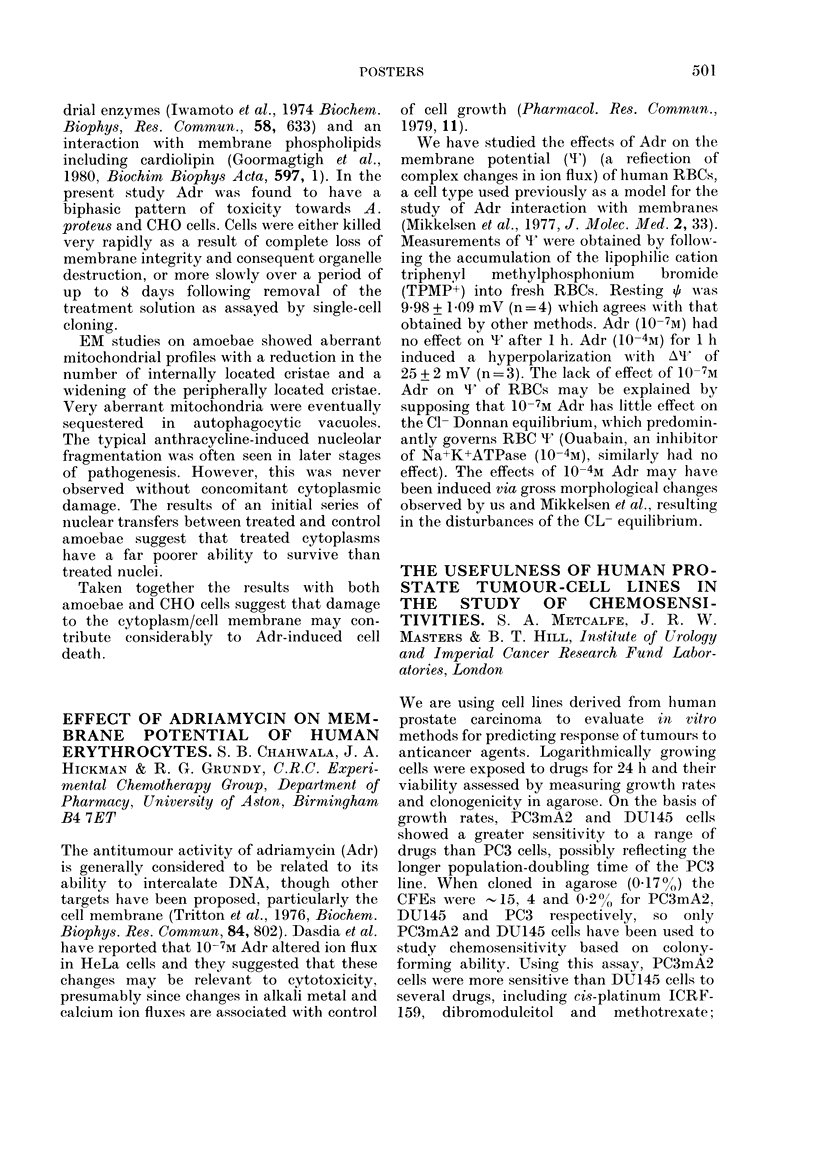

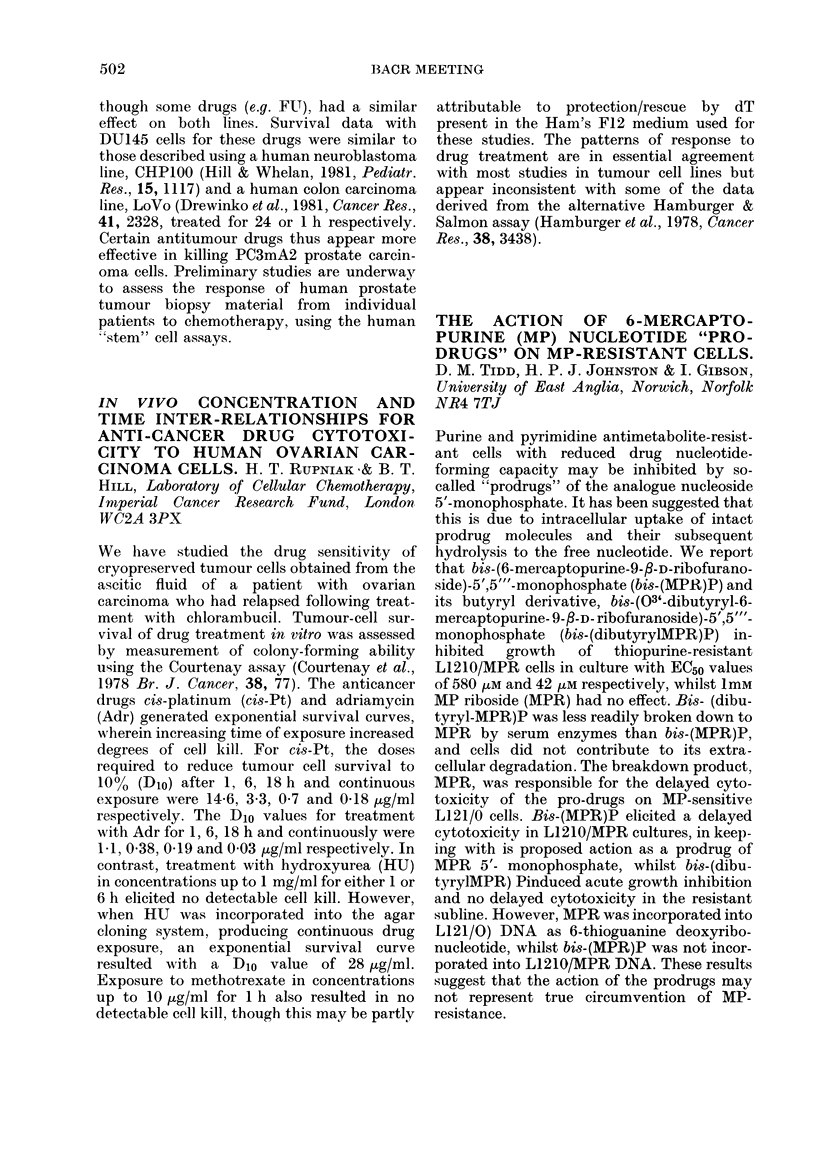

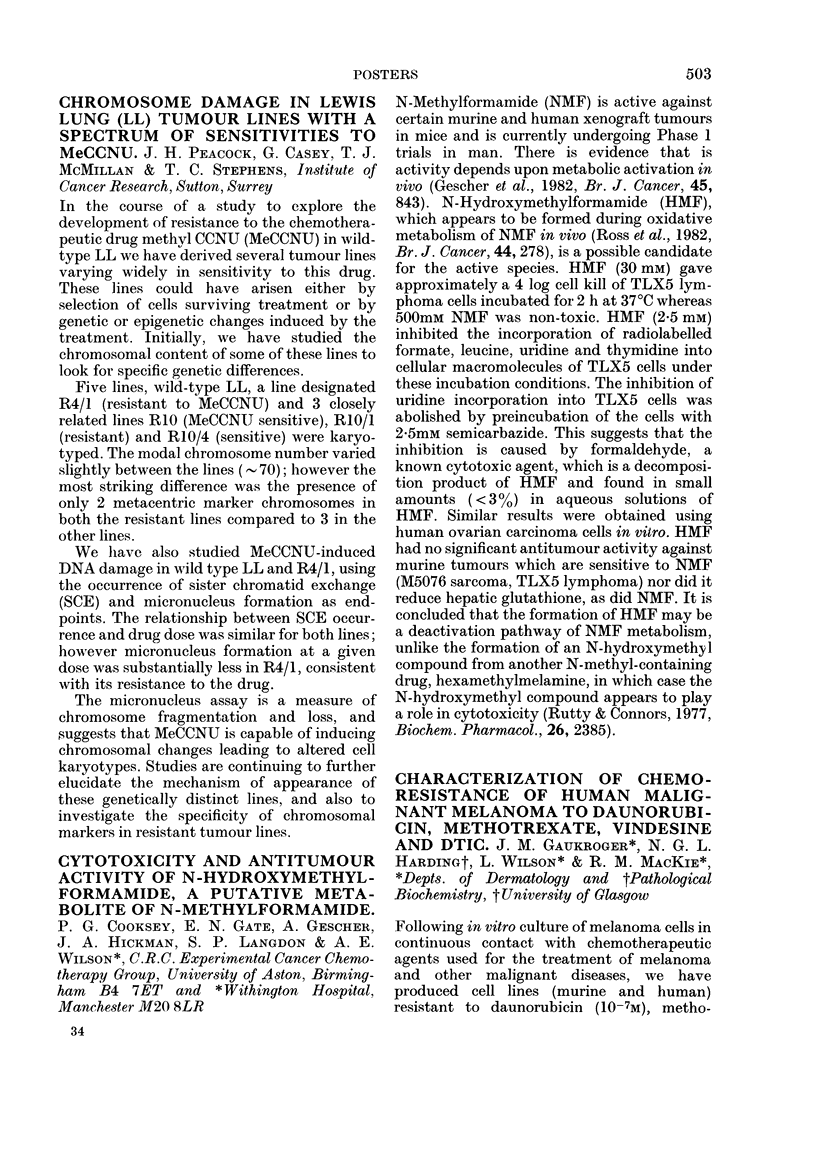

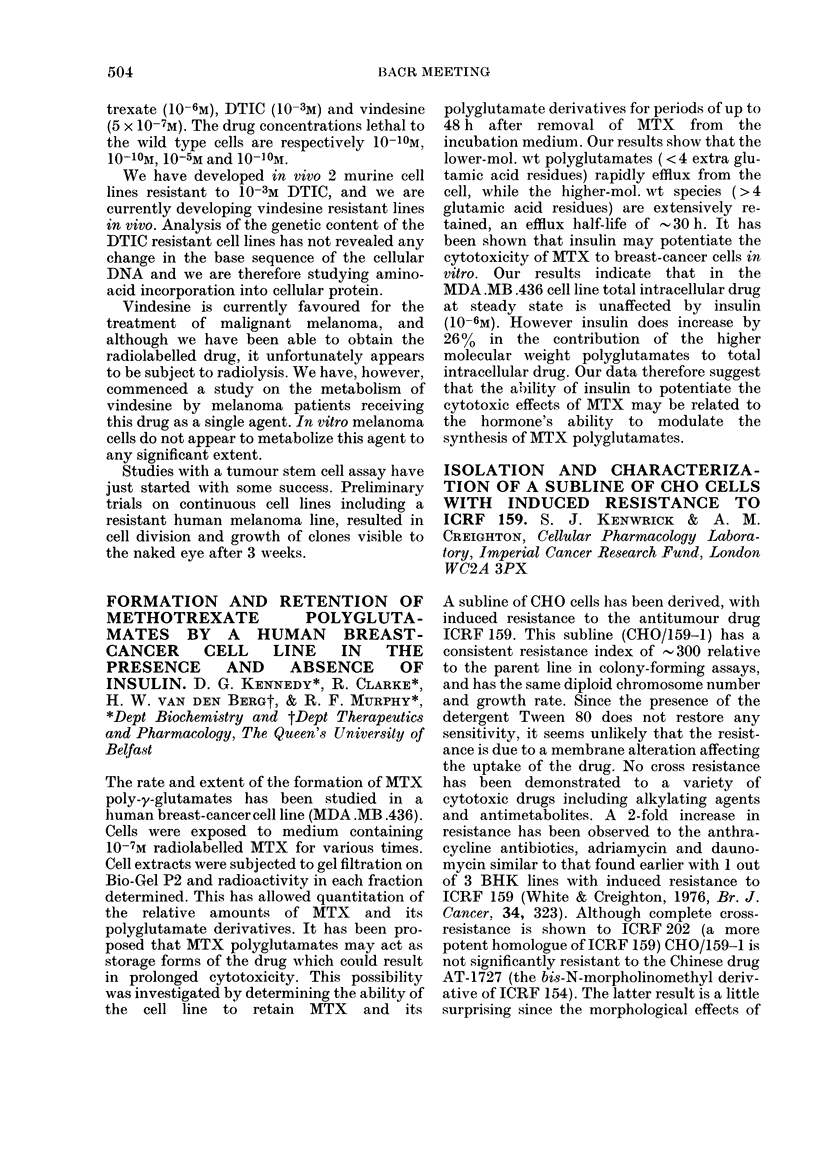

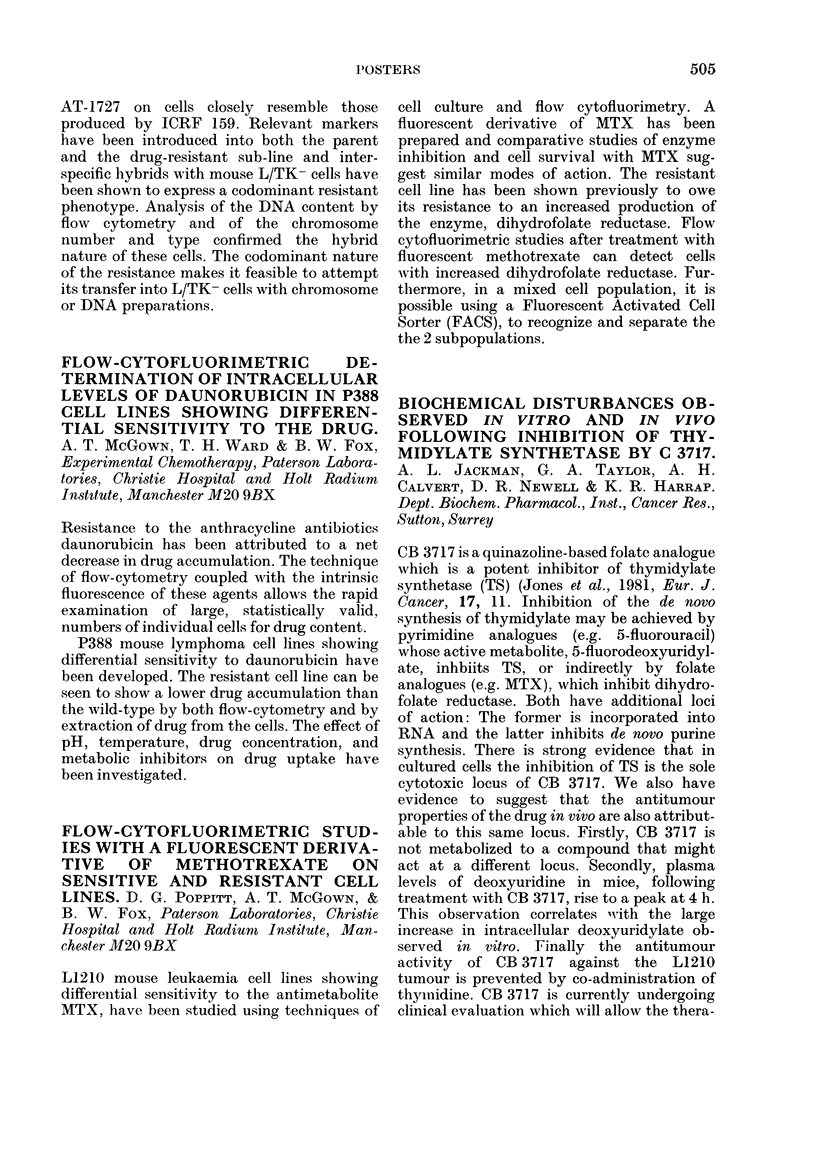

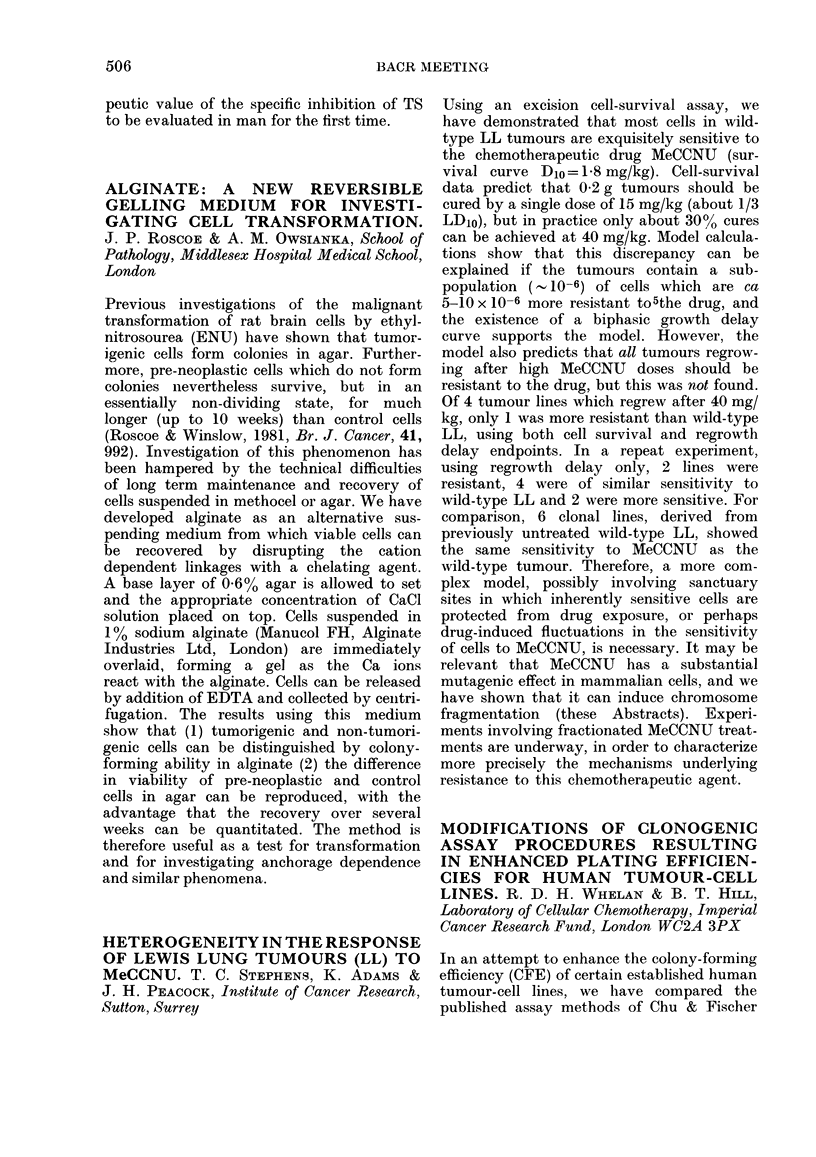

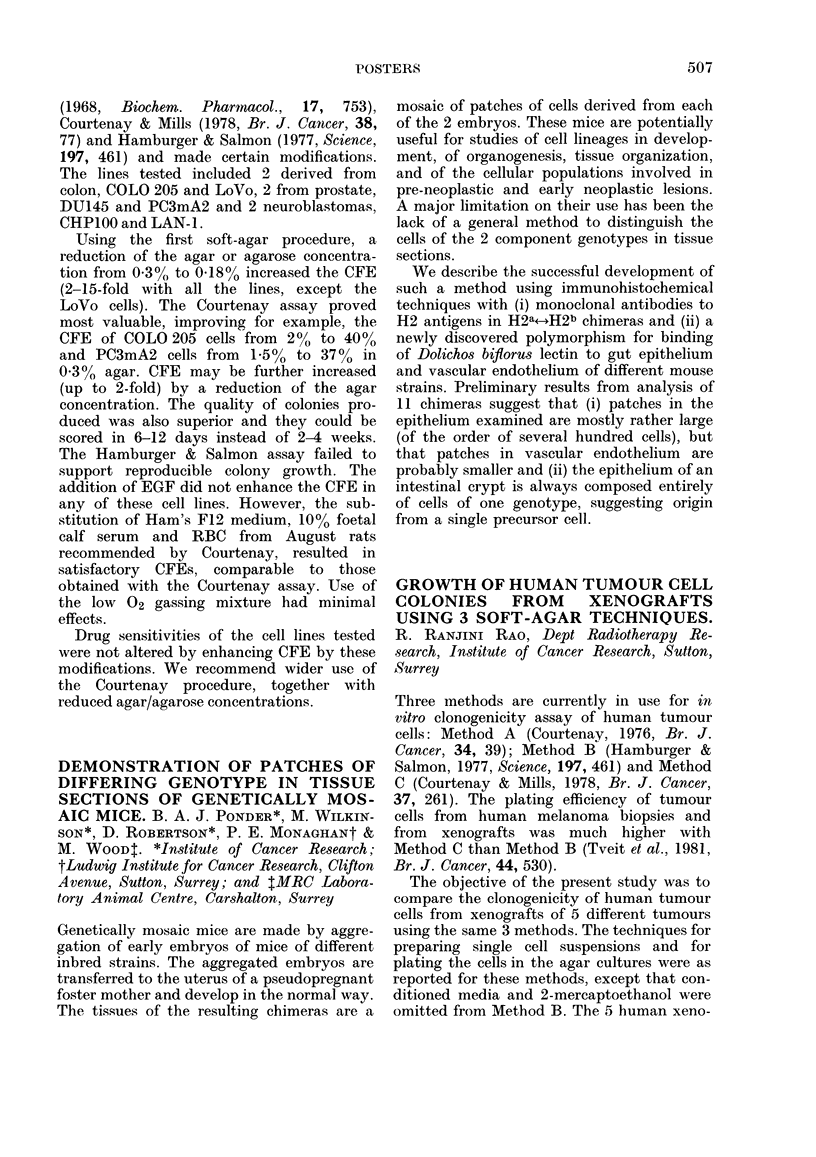

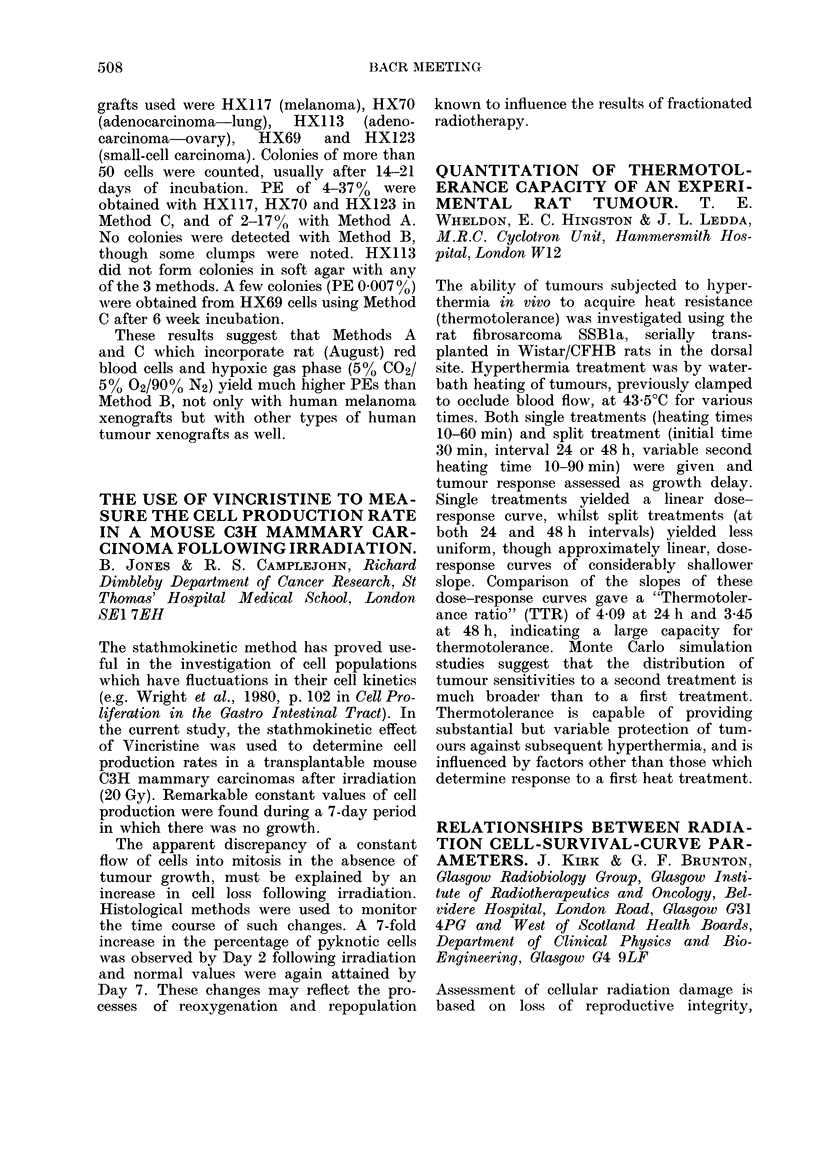

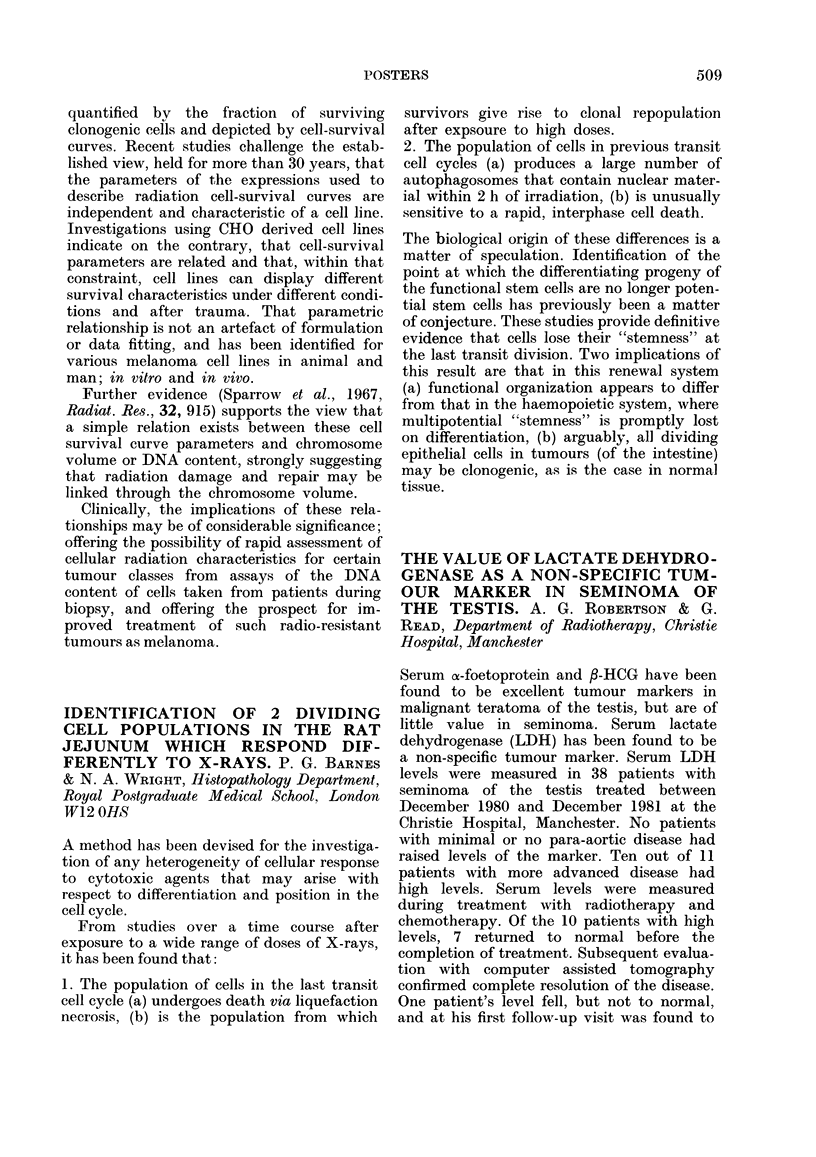

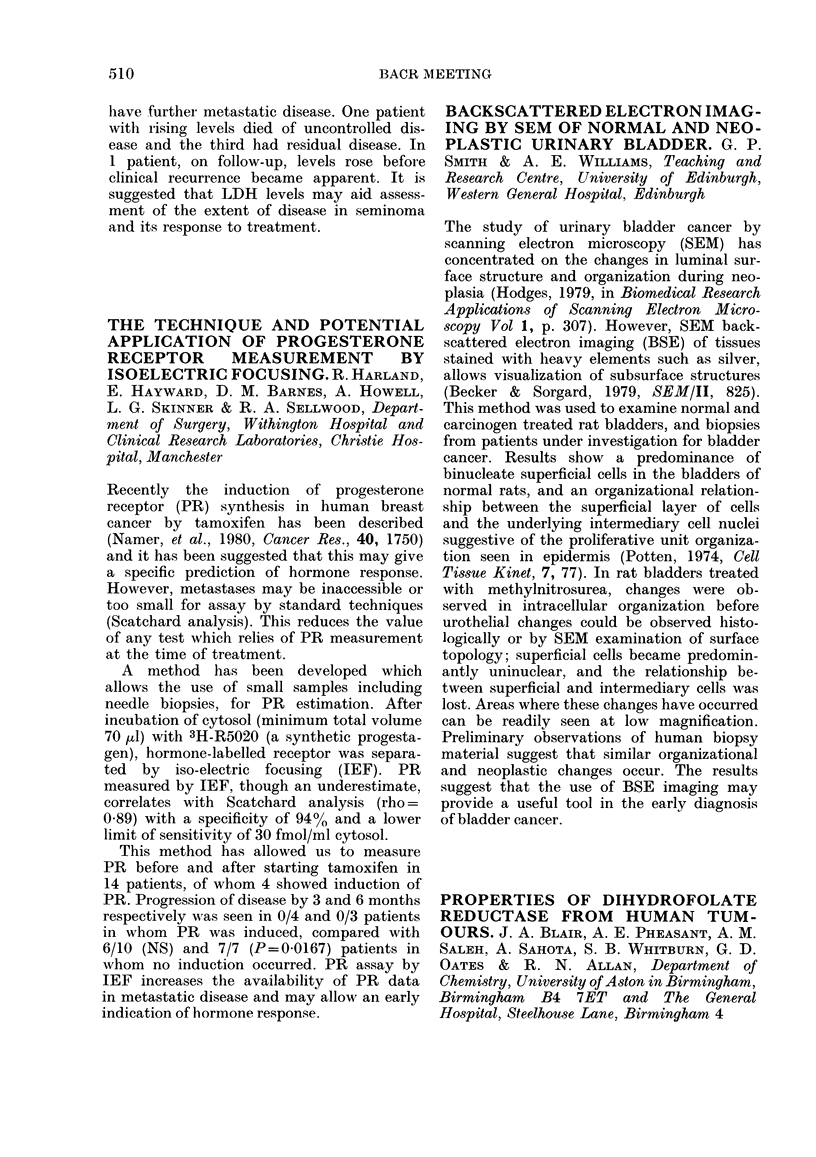

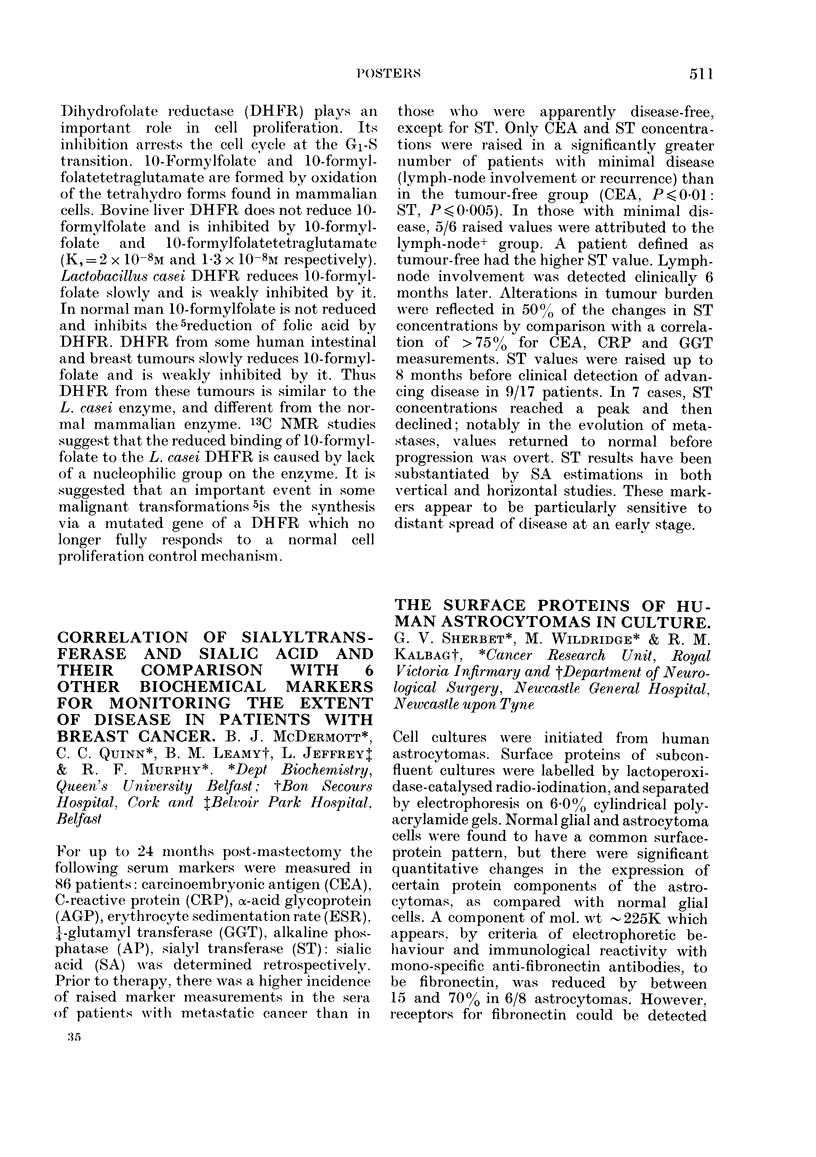

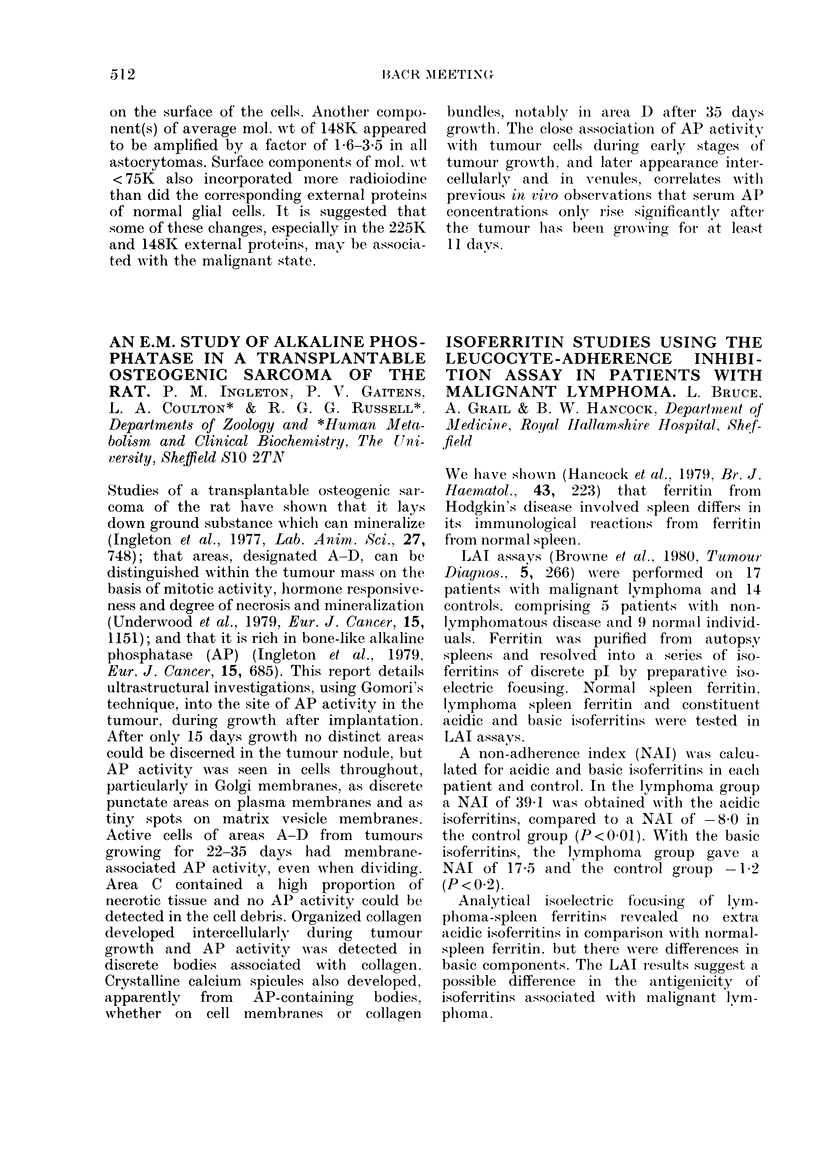

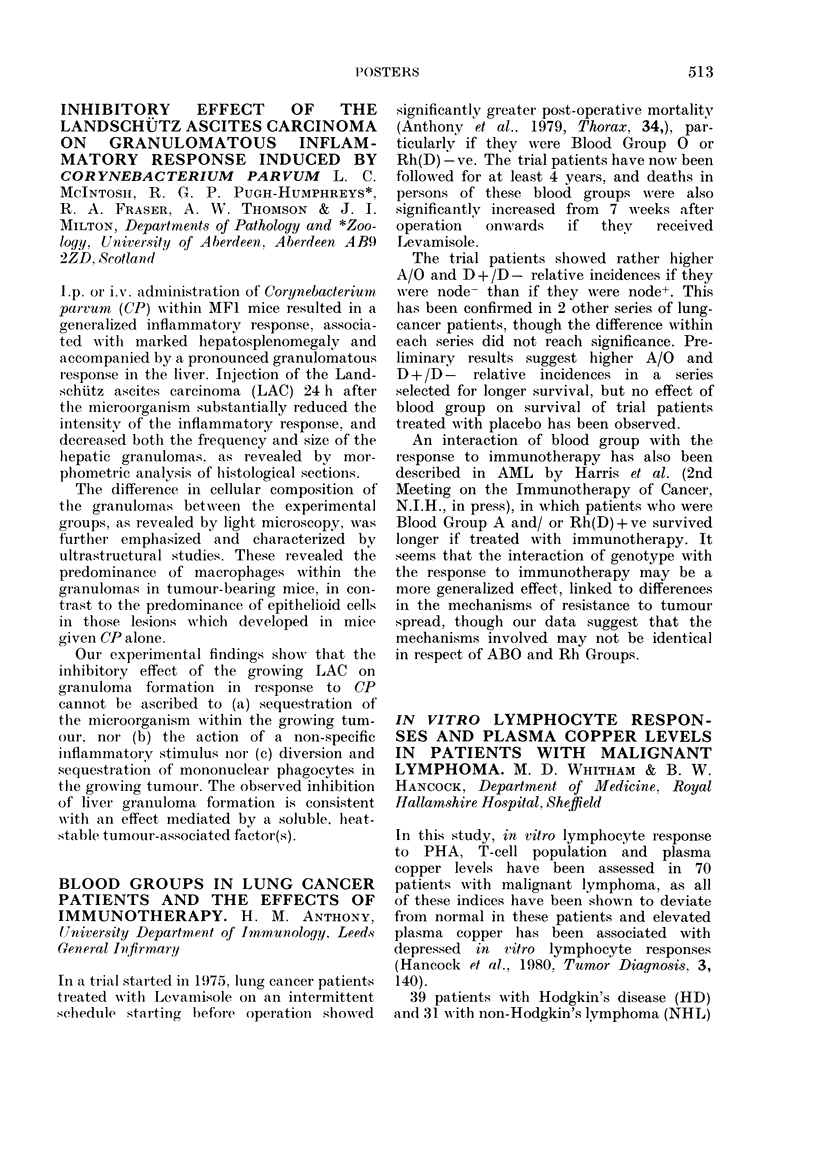

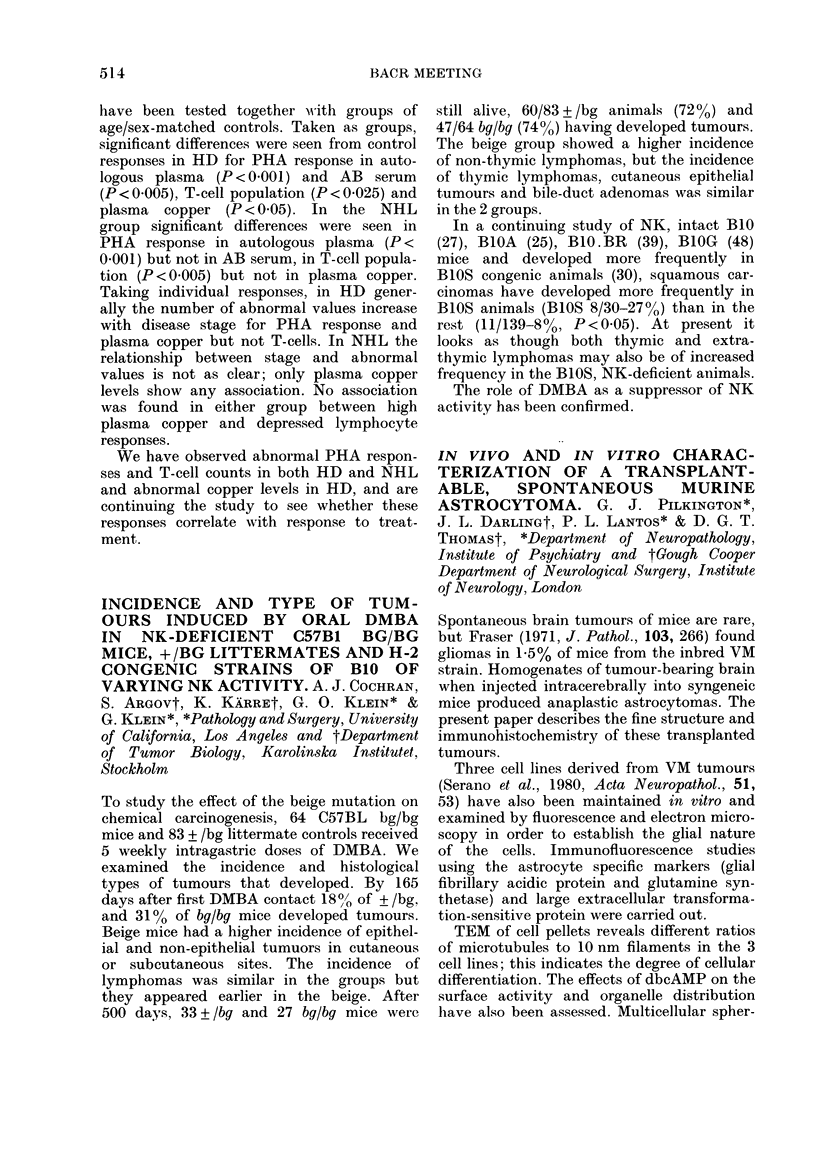

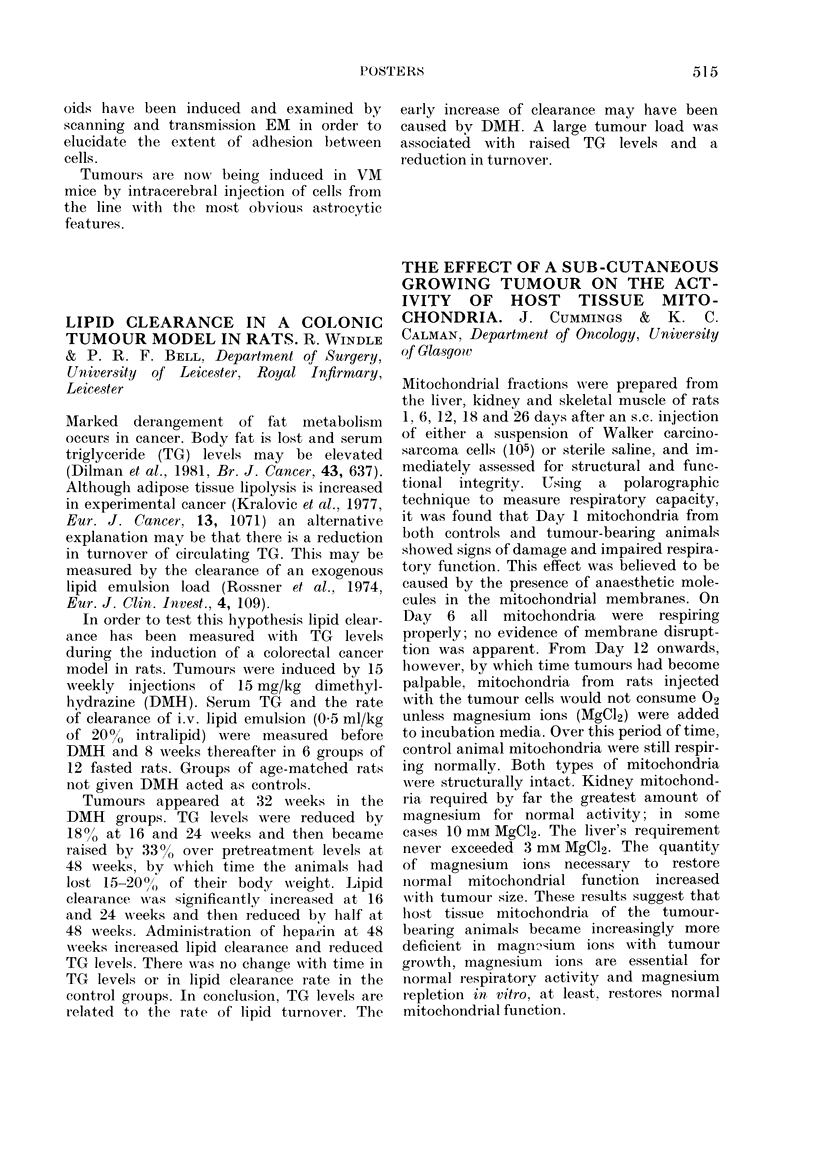

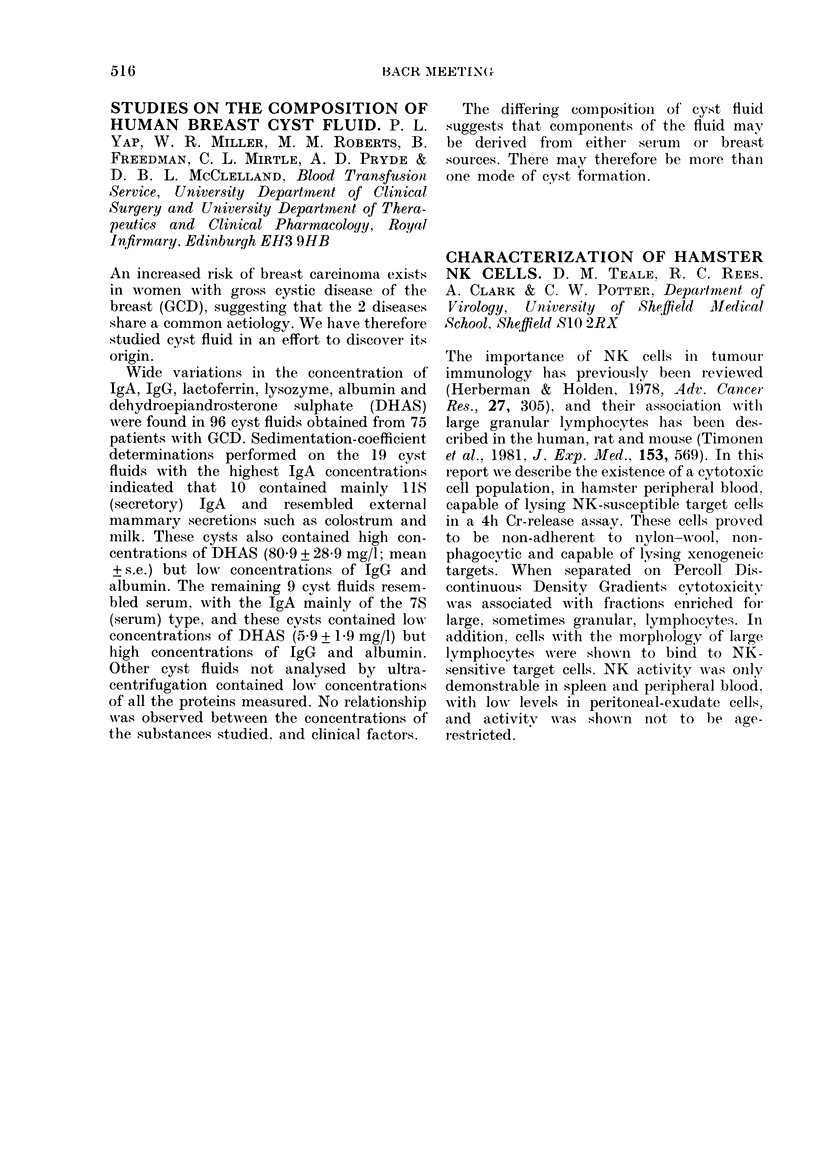


## References

[OCR_00575] Barranco S. C., Romsdahl M. M., Humphrey R. M. (1971). The radiation response of human malignant melanoma cells grown in vitro.. Cancer Res.

[OCR_04520] Bedford P., Fox B. W. (1981). The role of formaldehyde in methylene dimethanesulphonate-induced DNA cross-links and its relevance to cytotoxicity.. Chem Biol Interact.

[OCR_04857] Bonfante V., Bonadonna G., Villani F., Di Fronzo G., Martini A., Casazza A. M. (1979). Preliminary phase I study of 4'-epi-adriamycin.. Cancer Treat Rep.

[OCR_01545] Carr B. I., Laishes B. A. (1981). Resistance to the cytocidal effects of adriamycin is an early phenotypic change induced during hepatocarcinogenesis.. Br J Cancer.

[OCR_05722] Courtenay V. D., Mills J. (1978). An in vitro colony assay for human tumours grown in immune-suppressed mice and treated in vivo with cytotoxic agents.. Br J Cancer.

[OCR_00595] Courtenay V. D., Mills J. (1978). An in vitro colony assay for human tumours grown in immune-suppressed mice and treated in vivo with cytotoxic agents.. Br J Cancer.

[OCR_00522] Courtenay V. D., Mills J. (1978). An in vitro colony assay for human tumours grown in immune-suppressed mice and treated in vivo with cytotoxic agents.. Br J Cancer.

[OCR_04575] Cowen D. M., Double J. A., Cowen P. N. (1980). Some biological characteristics of transplantable lines of mouse adenocarcinomas of the colon.. J Natl Cancer Inst.

[OCR_03837] Dembo A. J., Van Dyk J., Japp B., Bean H. A., Beale F. A., Pringle J. F., Bush R. S. (1979). Whole abdominal irradiation by a moving-strip technique for patients with ovarian cancer.. Int J Radiat Oncol Biol Phys.

[OCR_04529] Double J. A., Ball C. R., Cowen P. N. (1975). Transplantation of adenocarcinomas of the colon in mice.. J Natl Cancer Inst.

[OCR_05061] Drewinko B., Patchen M., Yang L. Y., Barlogie B. (1981). Differential killing efficacy of twenty antitumor drugs on proliferating and nonproliferating human tumor cells.. Cancer Res.

[OCR_01109] Feit P. W., Rastrup-Andersen N., Matagne R. (1970). Studies on epoxide formation from (2S,3S)-threitol 1,4-bismethanesulfonate. The preparation and biological activity of (2S,3S)-1,2-epoxy-3,4-butanediol 4-methanesulfonate.. J Med Chem.

[OCR_06531] Fraser H. (1971). Astrocytomas in an inbred mouse strain.. J Pathol.

[OCR_05153] Gescher A., Gibson N. W., Hickman J. A., Langdon S. P., Ross D., Atassi G. (1982). N-methylformamide: antitumour activity and metabolism in mice.. Br J Cancer.

[OCR_05143] Hamburger A. W., Salmon S. E., Kim M. B., Trent J. M., Soehnlen B. J., Alberts D. S., Schmidt H. J. (1978). Direct cloning of human ovarian carcinoma cells in agar.. Cancer Res.

[OCR_00341] Hamburger A. W., Salmon S. E. (1977). Primary bioassay of human tumor stem cells.. Science.

[OCR_05709] Hamburger A. W., Salmon S. E. (1977). Primary bioassay of human tumor stem cells.. Science.

[OCR_06278] Hancock B. W., Bruce L., May K., Richmond J. (1979). Ferritin, a sensitizing substance in the leucocyte migration inhibition test in patients with malignant lymphoma.. Br J Haematol.

[OCR_06730] Herberman R. B., Holden H. T. (1978). Natural cell-mediated immunity.. Adv Cancer Res.

[OCR_05040] Hill B. T., Whelan R. D. (1981). Assessments of the sensitivities of cultured human neuroblastoma cells to anti-tumour drugs.. Pediatr Res.

[OCR_06269] Ingleton P. M., Coulton L. A., Preston C. J., Martin T. J. (1979). Alkaline phosphatase in serum and tumour of rats bearing a hormone-responsive transplantable osteogenic sarcoma.. Eur J Cancer.

[OCR_06229] Ingleton P. M., Underwood J. C., Hunt N. H., Atkins D., Giles B., Coulton L. A., Martin T. J. (1977). Radiation induced osteogenic sarcoma in the rat as a model of hormone-responsive differentiated cancer.. Lab Anim Sci.

[OCR_04918] Iwamoto Y., Hansen I. L., Porter T. H., Folkers K. (1974). Inhibition of coenzyme Q10-enzymes, succinoxidase and NADH-oxidase, by adriamycin and other quinones having antitumor activity.. Biochem Biophys Res Commun.

[OCR_06583] Kralovic R. C., Zepp F. A., Cenedella R. J. (1977). Studies of the mechanism of carcass fat depletion in experimental cancer.. Eur J Cancer.

[OCR_04585] Laverick M., Nias A. H. (1981). Potentiation of the radiation response of hypoxic mammalian cells by cis-dichlorobis(isopropylamine)trans-dihydroxy platinum IV (CHIP).. Br J Radiol.

[OCR_03532] Limas C., Lange P., Fraley E. E., Vessella R. L. (1979). A, B, H antigens in transitional cell tumors of the urinary bladder: correlation with the clinical course.. Cancer.

[OCR_00459] MACPHERSON I., MONTAGNIER L. (1964). AGAR SUSPENSION CULTURE FOR THE SELECTIVE ASSAY OF CELLS TRANSFORMED BY POLYOMA VIRUS.. Virology.

[OCR_06033] Namer M., Lalanne C., Baulieu E. E. (1980). Increase of progesterone receptor by tamoxifen as a hormonal challenge test in breast cancer.. Cancer Res.

[OCR_04806] Newlands E. S., Begent R. H., Kaye S. B., Rustin G. J., Bagshawe K. D. (1980). Chemotherapy of advanced malignant teratomas.. Br J Cancer.

[OCR_02376] Peter H. H., Dallügge H., Zawatzky R., Euler S., Leibold W., Kirchner H. (1980). Human peripheral null lymphocytes. II. Producers of type-1 interferon upon stimulation with tumor cells, Herpes simplex virus and Corynebacterium parvum.. Eur J Immunol.

[OCR_01889] Reading C. L., Brunson K. W., Torrianni M., Nicolson G. L. (1980). Malignancies of metastatic murine lymphosarcoma cell lines and clones correlate with decreased cell surface display of RNA tumor virus envelope glycoprotein gp70.. Proc Natl Acad Sci U S A.

[OCR_02395] Reid M. M., Craft A. W., Todd J. A. (1977). Serial studies of numbers of circulating T and B lymphocytes in children with acute lymphoblastic leukaemia.. Arch Dis Child.

[OCR_05615] Roscoe J. P., Winslow D. P. (1980). Increased ability of ethylnitrosourea-exposed brain cells to survive suspension in agar.. Br J Cancer.

[OCR_00811] Rutty C. J., Connors T. A. (1977). In vitro studies with hexamethylmelamine.. Biochem Pharmacol.

[OCR_05373] Rutty C. J., Connors T. A. (1977). In vitro studies with hexamethylmelamine.. Biochem Pharmacol.

[OCR_06721] Rössner S., Boberg J., Carlson L. A., Freyschuss U., Lassers B. W. (1974). Comparison between fractional turnover rate of endogenous plasma triglycerides and of intralipid (intravenous fat tolerance test) in man.. Eur J Clin Invest.

[OCR_00412] Salmon S. E., Hamburger A. W., Soehnlen B., Durie B. G., Alberts D. S., Moon T. E. (1978). Quantitation of differential sensitivity of human-tumor stem cells to anticancer drugs.. N Engl J Med.

[OCR_04141] Shaw D., Trotter J. M., Calman K. C. (1980). Plasma exchange to control sweats and pruritus in malignant disease.. Br Med J.

[OCR_01617] Sheahan D. G., Horowitz S. A., Zamcheck N. (1971). Deletion of epithelial ABH isoantigens in primary gastric neoplasms and in metastatic cancer.. Am J Dig Dis.

[OCR_03335] Shorthouse A. J., Smyth J. F., Steel G. G., Ellison M., Mills J., Peckham M. J. (1980). The human tumour xenograft--a valid model in experimental chemotherapy?. Br J Surg.

[OCR_01490] Sonakul D., Koompirochana C., Chinda K., Stitnimakarn T. (1978). Hepatic carcinoma with opisthorchiasis.. Southeast Asian J Trop Med Public Health.

[OCR_05949] Sparrow A. H., Underbrink A. G., Sparrow R. C. (1967). Chromosomes and cellular radiosensitivity. I. The relationship of D0 to chromosome volume and complexity in seventy-nine different organisms.. Radiat Res.

[OCR_01495] Thamavit W., Bhamarapravati N., Sahaphong S., Vajrasthira S., Angsubhakorn S. (1978). Effects of dimethylnitrosamine on induction of cholangiocarcinoma in Opisthorchis viverrini-infected Syrian golden hamsters.. Cancer Res.

[OCR_01536] Tomatis L., Cefis F. (1967). The effects of multiple and single administration of dimethylnitrosamine to hamsters.. Tumori.

[OCR_04967] Tritton T. R., Murphree S. A., Sartorelli A. C. (1978). Adriamycin: a proposal on the specificity of drug action.. Biochem Biophys Res Commun.

[OCR_06234] Underwood J. C., Melick R. A., Loomes R. S., Dangerfield V. M., Crawford A., Coulton L., Ingleton P. M., Martin T. J. (1979). Structural and functional correlations in parathyroid hormone responsive transplantable osteogenic sarcomas.. Eur J Cancer.

[OCR_01620] Voak D., Sacks S., Alderson T., Takei F., Lennox E., Jarvis J., Milstein C., Darnborough J. (1980). Monoclonal anti-A from a hybrid-myeloma: evaluating as a blood grouping reagent.. Vox Sang.

[OCR_03543] Voak D., Sacks S., Alderson T., Takei F., Lennox E., Jarvis J., Milstein C., Darnborough J. (1980). Monoclonal anti-A from a hybrid-myeloma: evaluating as a blood grouping reagent.. Vox Sang.

[OCR_04082] WALDMANN T., TRIER J., FALLON H. (1963). Albumin metabolism in patients with lymphoma.. J Clin Invest.

